# Electrochemical (Bio)Sensors Based on Nanotechnologies for the Detection of Important Biomolecules in Plants and Plant-Related Samples: The Future of Smart and Precision Agriculture

**DOI:** 10.3390/bios16020107

**Published:** 2026-02-06

**Authors:** Ioana Silvia Hosu, Radu-Claudiu Fierăscu, Irina Fierăscu

**Affiliations:** 1 National Institute for Research & Development in Chemistry and Petrochemistry-ICECHIM Bucharest, 202 Spl. Independentei, 6th District, 060021 Bucharest, Romania; fierascu.radu@icechim.ro (R.-C.F.); irina.fierascu@icechim.ro (I.F.); 2 Faculty of Chemical Engineering and Biotechnologies, University “Politehnica” of Bucharest, 313 Splaiul Independentei Str., 060042 Bucharest, Romania; 3 Academy of Romanian Scientists, 3 Ilfov, 050044 Bucharest, Romania; 4 Faculty of Horticulture, University of Agronomic Sciences and Veterinary Medicine of Bucharest, 59 Marasti Blvd., 011464 Bucharest, Romania

**Keywords:** plant, electrochemistry, biosensors, nanotechnology, wearable sensors, crop loss, smart and precision agriculture, plant health

## Abstract

Considering the present environmental concerns, nanomaterial-based methods should be applied to achieve the bioeconomic sustainability initiatives and climate change mitigation. Plants and plant extracts are one of the most underused biomass and bioactive ingredients resources. Moreover, nowadays crop loss is one of the main problems that the world faces, together with the depletion of natural resources, increasing population and limited arable land, leading to increased food scarcity and demand. To correctly attribute/use plant-based bioresources or to rapidly decide which farming operations should be performed before crop loss, we should be able to properly characterize plants or plant-based resources by the desired useful characteristics, such as (bio)chemical characteristics, rather than simply observing physical traits of plants (because, when these traits become visible, it may be too late for crop loss mitigation). Plant crops could be optimized, for example, using electrochemical methods that assess the nutrient uptake and nutrient use efficiency (NUE) or the oxidative stress burst encountered before crop loss, in order to improve crop yields and crop quality. Other different important analytes (such as hormones, pathogens, metabolites, etc.) or plant characteristics (such as genus, species, phylogenetic analysis, etc.) can be evaluated with these electrochemical sensors and methods. In the present review, we focus on the application of nanomaterials/nanotechnologies for the development of fast, accurate, accessible, cost-effective, sensitive and selective analytical electrochemical methods for the detection of different relevant biomolecules in plants or plant-related samples (plant extracts, plant cells, plant tissues, and/or plant-derived natural drinks/foods, as well as entire plants/plant parts), both *in vivo* vs. *ex vivo* and *in situ* vs. *ex situ.* This review systematically presents and critically discusses the outcomes of current electrochemical methods (both applied in the lab or as wearable/implantable sensors) and the future perspectives of these nanotechnology-based sensors, with an accent on wearable sensors for smart and precision agriculture, as real-world sensing technologies with significant practical impact. The novelty of this article is the abundance of electrochemical analytical parameters gathered and discussed, for such a large number of analyte categories.

## 1. Introduction

According to the FAO (Food and Agriculture Organization of United Nations), food demand will increase by 50% by 2050, of which 80% is plant-based [1]. In recent years, the growing world-wide population and depletion of natural resources has led to an unsustainable food system that cannot feed the entire global population in an eco-friendly manner, mitigating climate change and the damage to biodiversity and entire ecosystems. Up to 40% of the entire world-wide crops is lost nowadays due to plant pests and diseases. Crop loss appears as a consequence of the poor quality of crops, as well as from drastic growing conditions involving both biotic and abiotic stress (drought, erratic rainfall, temperature oscillations, greenhouse gases, pollution, pest/bacteria/virus related diseases, overuse or misuse of agrochemicals that leads to further soil and water contamination, etc.). The traditional agricultural methods cannot keep up with the increasing food demand, as agriculture provides the biggest part of food supply. The needs to improve crop quality and to increase crop yields, to decrease resource consumption (water, plant treatments, energy, etc.), or to decrease the cost of farming are identified in different stakeholders such as small or large farmers, agrochemical producers, phenotyping or monitoring devices producers, etc. Crop productivity, as well as postharvest storage, is very important and could be improved using different analytical methods for diseases diagnosis [2], screening agrochemical products (such as biostimulants, pesticides, fertilizers and other plant treatments), or monitoring hazardous substances and plant health in real-time.

Farmers face growing demands for more and better crops, facing, at the same time, limitations regarding the quantitative analysis of crop performance, and lack of real-time data to rapidly optimize farming operations (as they operate in a time-sensitive environment with quick and mostly unpredictable changes). These problems persist because of the scarce technologies for precision and smart agriculture (like chemically modified wearable or implantable sensors for real-time monitoring of plants that can gather data remotely from large surfaces). A sustainable food system could be achieved using smart and precision farming, even for small farmers. Smart and precision farming refers to implementation of new technologies for maximization of crop yields and crop quality, with data mining and integration in large networks, such as the Internet of Things (IoT) [3].

The lack of technologies based on electrochemical (bio)sensors that can give information about the chemical, biochemical, and biological traits of plants, not only physical, morphological, or morphometric traits, such as plant imaging (e.g., most phenotyping methods) is the most important drawback in the development of smart and precision agriculture. Classical chemical and biochemical analysis consist of labor-intensive, time-consuming, high-cost, destructive, difficult to use and complex methodologies [4] not suitable for field measurements. The World Economic Forum selected wearable plant sensors in 2023 as one of the Top 10 Emerging Technologies, considering that these smart analytical tools will be relevant in the next generation of AgriFood and AgriTech practices [5].

Diagnosis of diseases can be performed using the signal molecules released during pathogenic attacks [2]. An infected plant will produce significantly different amounts of VOCs (volatile organic compounds) when compared to healthy plants [6]. The signature of different chemicals released during pathogenic infections contains different pieces of information that can be further used as biomarkers in the diagnosis of disease in crops. For example, methyl salicylate is an important marker for biotic stress in plants and glucose is an important marker for abiotic stress in plants. All of these molecules have already been detected in the literature in plant-related samples. Choosing the right materials for this kind of sensor is important as they need to be biocompatible, non-cytotoxic, eco-friendly, and biodegradable/obtained from a natural source (if possible), to encourage a circular bio-economy system and respect a sustainable use of natural resources. Decreasing the cost of the technologies, to make them affordable and appliable for field measurements, is also very important. Field measurements imply a scaling-down of complex devices to make them portable and easy to handle. Even though some materials were already demonstrated to be efficient in detecting the same target molecule in other biological systems, they might not be sensitive, selective, or accurate enough for plant-related samples. Sometimes, the materials need to be flexible and stretchable (to be attached on non-flat surfaces of plants). Other times, the materials need to be rigid and have high mechanical resistance to penetrate the upper layer of the plants to reach the core of the plant part (such as sap or xylem). Non-invasive and non-destructive materials/technologies/methods are needed to create efficient electrochemical sensors for plants. Interconnectivity of different domains is also required: electrochemistry, biosensing, bioelectronics (or at least electronics), hardware and software engineering, chemistry, plant biology, etc.

Even though electrochemical analysis has become a new method for plant analysis in recent years, most of the technologies applied in sensors are not yet applied to entire living plants or parts of plant samples. This review aims to present these technologies, in order for scientists or different interested stakeholders to better understand the state-of-the art for scientific development of electrochemical (bio)sensors applied to plants or plant-related samples. All internal activities taking place in plants (plant physiology: nutrient uptake, stress responses, signaling pathways, etc.) that have been studied in the last 14 years with electrochemical sensors are considered in this review.

Most of the review consists of a comprehensive section of different categories/important molecules detected in plants and plant-related samples, with a description of the materials used for those categories. This section presents most of the approaches used for plant-related samples, including studies of plants (e.g., phylogenetic and species, through electrochemical fingerprinting). Following this, three different shorter sections are presented, dedicated to direct *ex vivo* or *in vivo* measurements on plants or plant parts: wearable sensors, commercialization of electrochemical sensors for plants, and future perspectives and conclusions.

The novelty of this review consists in offering a broader picture of the most important biomolecules in plants, focusing not only on a few examples, but offering many examples and comparing their analytical parameters in an extensive study presented as a table in the Supplementary Information.

## 2. Fundamentals of Electrochemical Sensors: Why Electrochemistry?

Traditional analytical methods (gold standards, such as mass spectrometry or chromatography) used to study different aspects of plants are usually expensive, time consuming, complex, require expert operation, require sample treatments, are usually performed in laboratory conditions, produce high amounts of organic solvent waste, and cannot be adapted for real-time *in situ* and *in vivo* monitoring. Even though spectroscopic methods such as FTIR (Fourier Transformed Infrared Spectroscopy) or Raman spectroscopy can provide comprehensive information about chemical and physical composition, they also often require complex equipment and sample preparation [7], and are often incompatible with in-field measurements, even though there are some emerging Raman nanosensors compatible with plant health monitoring [8]. In fact, making nanosensors compatible with Raman (or another technique) while also having electrochemical compatibility only enhances the potency of the (bio)sensors, by gathering complementary information at the same time, and adding value to the multimodal sensors developed. For example, plant biostimulants (PBs) would need to have both strong antioxidant and antibacterial properties and these properties could be better assessed using fast and cheap electrochemical methods, compared to other expensive analytical methods.

Electrochemical analytical systems usually comprise a working electrode (WE), a counter electrode (CE), and a reference electrode (RE), that measure the redox (reduction-oxidation) exchange of electrons transferred between different entities (molecules, ions, electrodes, etc.), in the presence of an electrically conducting system and electronic devices that transduce the electrical signal. The system reports the electrochemical response (both from transforming electrical energy into chemical energy or vice versa) obtained from electrical resistance, current, or potential changes at the solid electrodes–liquid electrolyte interfaces, but is not necessarily limited to this aggregation state. This electrochemical response results from a reaction with the targeted analytes and allows quantitative detection by controlling applied potentials or currents. An enhanced electrochemical response depends on a different step-by-step process, often based on the electron transfer at the surface of the electrode, mass transport and possible chemical reactions. Correct interpretation of obtained experimental data depends on these steps, and helps distinguish kinetics from transport limitations.

Different electrochemical methods already exist. Some of them have been applied for the developed sensors presented in this review. A brief description of these methods is necessary for the readers to be able to understand the advantages and limitations of these methods. All of the electrochemical methods are governed by the previously described fundamental principles, but different methods have different particularities. Voltammetry is the electrochemical category where current is measured, while potential is varied. The applied potential sweep profile defines the type of voltammetric technique used, including cyclic voltammetry (CV), linear sweep voltammetry (LSV), differential pulse voltammetry (DPV) or square wave voltammetry (SWV). CV sweeps the potential forward and backward and the resulting current–potential plot usually provides information about the mechanistic insight of the redox systems. This way, CV reveals electrochemical redox potentials and kinetics (reversible, quasi-reversible and irreversible behaviors of the redox systems), or shows adsorption, catalysis and coupled chemical reactions, but limits sensitivity and detection limits and usually has high capacitive (non-faradaic) currents, not suitable for trace analytes. Enhanced EC signals are obtained with more complicated applied potential changes. In LSV, potential changes linearly in one direction, making the experiment faster and easy to interpretate, especially when the reverse scan might negatively influence the electrode surface, but still suffers from capacitive current involvement. In DPV, short potential impulses are superimposed on the base sweep of potential, to increase sensitivity of the method and significantly decrease the capacitive currents (because the current is measured before and after the impulse and presented as a difference of these two currents, eliminating the noise that results from non-faradaic current). This way, DPV becomes much more suitable for trace analytes, with better redox peak resolution, but does not offer mechanistic information (as CV and LSV), and requires more optimization of the working parameters. The SWV represents a combination of DPV (a stair case potential ramp of the potential) with superimposed square-wave potential. SWV is the fastest method from the voltametric techniques, has the highest sensitivity, and is suitable for both reversible and irreversible systems, with excellent capacity to eliminate capacitive current, due to the manner in which the electrical potential is applied (one forward pulse and one reversed pulse, of equal magnitude, with plotting of the current difference obtained from the forward and reversed impulses). Fast electron transfer processes should be examined with SWV.

On the other hand, there are the methods that measure currents as a function of time, at a fixed applied potential. This applied potential is chosen based on its ability to oxidize and reduce the targeted analyte under diffusion-controlled conditions. When compared to voltammetry, these methods offer several advantages (such as real-time monitoring, signal simplicity and better sensor development), but also several limitations (unknown redox behaviors, low–moderate selectivity, prior knowledge of the redox potential, possible fouling at the surface of the electrode, and background drift over time).

A special electrochemical method is Electrochemical Impedance Spectroscopy (EIS), in which one applies small alternating perturbations (originating from alternating current—AC, as opposed to the other methods that use direct current—DC) and measures frequency-dependent response. When compared with voltammetry and amperometry, impedance offers excellent mechanistic information, has minimal perturbance on the system (being non-destructive and measuring reaction rates without consuming the analyte), offers indirect quantification with very high sensitivity, and makes continuous monitoring possible. Even though it has several advantages, this method has a model-based interpretation and needs equivalent circuit selection, being time consuming and less intuitive.

In electrochemical sensor development, both voltammetry and amperometry should be combined for correct optimization of the detection parameters. Impedance can used for finding out why and where resistance occurs, but it needs special attention for interpretation. The chosen method should also be suitable for the final outcomes and benefits of the developed sensor, in accordance with their application [9].

Electrochemistry is convenient because it is suitable for *in vivo* monitoring, can reach high sensitivity, and is cost attractive, and it is easy to miniaturize and integrate sensors in portable devices for real-time *in situ* monitoring [10], along with microfluidic devices [11]. Monitoring of the electrochemical signal can be fast, allowing field measurements, in contrast with classical complex, expensive methods and equipment. Electrodes used in electrochemistry can be easily produced in large quantities for large-scale use (scale-up phase). Multiplexing is also a desirable characteristic and can be performed with electrochemical (bio)sensors to obtain complementary information regarding plant health. Even though electrochemical sensors are one of the most important categories of (bio)sensors, the scientific studies related to their application in agriculture and biological sciences are scarce, but this technique has the potential to achieve the necessary demands and goals.

## 3. What to Expect from This Review

After applying the PICO strategy to survey the literature for this review (Table S1), we have identified different categories of important molecules to be detected in plants. Most of the subsections of the review are structured based on a specific category of analytes identified in plants. For a better understanding, the first subsection describes some basic processes in plants (more as examples of plant physiology).

Table S2 (Supporting Information) presents an extensive survey of the electrochemical analytical methods used for detection of different categories of biomolecules important in plants (such as hormones, enzymes, heavy metals, proteins, phytochemicals, primary and secondary metabolites, ions, pesticides, herbicide, pollutants, alkaloids, pathogens, reactive oxygen/nitrogen species, antioxidants, etc.), other important plant characteristics (study of taste, plant phylogenetics, or other plant studies), or for the evaluation of different curative potentials of plants (such as anti-HIV [12], anti-cancer, or anti-diabetic properties). This table also describes the identified analytical performance of the methods and the plants on which they were implemented. Some of the electrochemical analytical methods presented in this extensive table are described in the following subsections, presented according to their category of analytes.

Even though there are many emerging nanoparticles biosynthesized using plant extracts for metal reduction and metal nanomaterial synthesis (for example, phytosynthesis of CuO nanoparticles using the extract of *Fortunella japonica* fruits as a reducing and stabilizing agent [13]) and followed by the further use of the CuO nanoparticles in electrochemistry), if the application does not include analyzing plant-based samples, the topic is not covered in this review. Another emerging category is the plant substrates used for production of sensors from reusable biomass (such as creating graphene from phenolic resin-based substrates such as leaves [14]), but again this is not the purpose of the current review, and this subject will not be covered. Soil-related sensing, wastewater treatment plants, waste waters, water used for crop irrigation or other agricultural processes, or any kind of water-related sensing, are not included as sample matrix.

Most reviews available in the literature do not describe a wide range of plant biomarkers and contaminants, but simply present classical biosensors and laboratory-based strategies [8], creating a literature review gap. This review covers this literature gap, describing both laboratory-based and real-application wearable electrochemical sensors, with discussions about their real environmental monitoring scenarios.

## 4. Important Aspects of Plant Physiology

Plant physiology is directly related to plants growth conditions. All of the mechanisms in plant physiology are still not understood. One of the most encountered stresses is the one determined by sodium chloride that can induce many consequences within a plant: a change in chlorophyl concentration, increase in osmotic regulators (such as proline) because of osmotic stress, and ionic stress, which will eventually lead to increased levels of reactive oxygen/nitrogen species (oxidative stress) [15]. The exact mechanisms of reactive oxygen species (ROS) in signaling pathways, signal transduction, or cell damage/death are not completely studied and understood. Even though hydrogen peroxide is one of the most stable forms of ROS which participates in electron transfer during photosynthesis, there are other species that are more damaging, with lower stability, impacting steady state concentrations in physiological media (such as peroxynitrite).

Primary metabolites (such as amino acids, carbohydrates, lipids, polyamines, glycine betaine, glucose, sucrose, etc.) are essential for the proper growth and development of plants and microorganisms. One category of primary metabolites includes osmolytes and osmoprotectants. Secondary metabolites (phenolics, terpenoids, and nitrogen-containing compounds—alkaloids) have no direct role in the growth and development of plants, but have an important role in the defense mechanisms against biotic stress and enhance resistance against abiotic stress [16]. The study of metabolites (plant metabolomics) is usually performed with GC-MS, LC-MS and NMR, but these methods, as stated above, are inappropriate for smart and precise agricultural real-time monitoring.

One category that is particularly studied with (bio)sensors is represented by the hormones and phytohormones. Hormones such as salicylic acid (SA) play important roles in the modulation of specific gene regulation and induction of various defense mechanisms of plants, and cooperate with other elicitors to boost synthesis of secondary metabolites.

The need to study plant physiological traits and understand different pathways/mechanisms of action, makes the development of electrochemical sensors even more necessary. The following subsections present the molecules that play important roles in plant physiology and are considered as targets for electrochemical detection.

## 5. Important Molecules in Plants and Their Laboratory-Based Electrochemical Detection

### 5.1. Markers for Biotic Stress

Methyl salicylate has been identified as one of the most important volatile organic compounds (VOCs) released by plants during a biotic stress event, such as fungal pathogen infection [17]. Methyl salicylate (MeSA) is also produced by wintergreen’s enzymes when the wintergreen plant is stressed (pathogen/pest attack). MeSA is also involved in Systemic Acquired Resistance (SAR) and long-distance plant–plant communication. A simulation of stress can be produced by maceration of wintergreen leaves in warm water, when methyl salicylate (MeSA) is produced, mainly through wintergreen oil (the real sample for this study). A bi-enzyme electrode was prepared using alcohol oxidase and horseradish peroxidase (as biorecognition elements) through a molecular tethering approach, on a multi-walled carbon nanotube (CNT) support, on a rotating disk electrode (RDE) [6]. The MWCNTs act as immobilization supports, as they have a large surface area to volume ratio, excellent conductivity, and can create non-covalent linkage with the pyrenebutanoic acid succinimidyl ester (PBSE), a method called molecular tethering (through π-π stacking). A similar approach was used for detection of MeSA using two other enzymes, salicylate hydroxylase and tyrosinase enzymes, for near-practical conditions (simulated for synthetic uninfected and soybean-aphid infected plants), using screen-printed carbon electrodes instead of RDE, and the same tethering method, with RSDs of 8.8% compared to 6.6% for the previously described study [17]. Nevertheless, the bi-enzyme electrochemical sensors were only tested in the liquid aqueous phase, under controlled laboratory conditions, and are not yet field-deployable, but they are compatible with near-real-time monitoring and early stress detection. Sugars were also detected in apple juice, using an enzyme-free sensor that demonstrated relevant *in planta* monitoring in real plant sap, but measurements still require sap extraction and no long-term continuous monitoring is presented [18]. The graphene–MWCNT–AuNP of the enzyme-free sensors improves long-term stability. Electronic noses have already been described as being able to detect VOCs in plants, but they are limited to sensor drift due to humidity and temperature, need frequent calibrations, and have limited chemical specificity [19]. Electronic noses are also mentioned for the detection of pathogens (e.g., *Pectobacterium carotovorum*, [20]), and the same paper incorporates integration of sensor nodes and sensors for smart farming, with power management, data transmission and modular sensors, and with IoT compatibility and outdoor deployable readiness of the system, but with no specific biomolecular specificity and without direct *in planta* studies, being a conceptual paper. Green leaf volatiles were also detected in reference [21]. The materials for the sensors of these markers are described in their subcategory of analytes.

### 5.2. Markers for Abiotic Stress

Different molecules can be considered markers for abiotic stress, and some of them have their own section in this review (such as reactive oxygen species, ROS). A particular bio-electrochemical sensing technique is presented for the detection of phytochelatin (cysteine-rich peptides), cysteine, and glutathione [22]. Cysteine and glutathione are peptides produced in plants when they are under heavy metal stress [22]. For this purpose, the determination of cysteine-rich peptide (CRp) generated by *Nicotiana tabacum* sample cells exposed to cytotoxic levels of Cd was performed using Cu electrodes modified with bimetallic nanoparticles. Other sensors that detect phytochelatin and cysteine are also described [23]. Quinoline [24] and sugars were also detected in apple juice [18]. Other approaches and biomarkers were also described in reference [25]. The materials used for developing the sensors for these markers are described in their subcategory of analytes.

### 5.3. Hormones/Phytohormones

The regulation of phytohormones in plants is really complicated and involves transport, perception, signaling, and biosynthesis [26]. A thorough study of hormones in plants could help us enhance tolerance to both abiotic or biotic stress consequences. The cooperation of phytohormones in plants is one of the key factors in the regulation and detection of different hormones, making them of essential interest.

IAA (indole-3-acetic acid) and SA (salicylic acid) were detected using paper-based analytical devices in plant samples (different parts of pea seedlings, sampled as disks) [26]. The electrodes were disposable double-sided conductive carbon tape on indium tin oxide-coated glass (ITO) and modified with multi-walled carbon nanotubes (MWCNTs). As an electrolyte, PBS was used between filter papers, and samples were still excised from plants. Nevertheless, no continuous or *in vivo* monitoring was performed, but the sensor is suitable for disposable field tests.

Ethylene is a phytohormone that appears as a gas when fruits and plants ripen. This gas is usually detected with IR, GC, and electrochemistry. The change in resistivity of a copper complex (as copper is a co-factor at the ethylene receptors of the fruit) with the binding to ethylene can be assessed using chemoresistive sensors [27]. The sensors were tested under industrial and semi-field conditions, with a focus on postharvest fruits and supply chains. The sensors help make decisions in ripening and spoilage control, which has high agronomic value, but use a respiration chamber in a closed gas circuit (not exactly field compatible).

**Figure 1:**
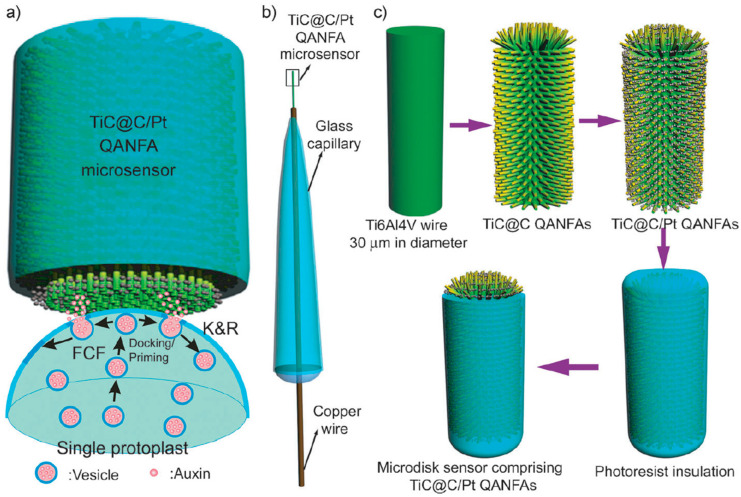
(**a**) Amperometric monitoring of auxin efflux from single protoplasts by vesicular exocytosis; (**b**) the TiC@C/Pt-QANFAs microdisk sensor; (**c**) the main processes for the fabrication of this sensor. Reproduced from Liu, J.T.; Hu, L.S.; Liu, Y.L.; Chen, R.S.; Cheng, Z.; Chen, S.J.; Amatore, C.; Huang, W.H.; Huo, K.F. Real-time monitoring of auxin vesicular exocytotic efflux from single plant protoplasts by amperometry at microelectrodes decorated with nanowires. Angew. Chem. Int. Ed. 2014, 53, 2643–2647 [28] with kind permission from WILEY. Copyright © 2014 WILEY-VCH Verlag GmbH & Co. KGaA, Weinheim. All rights reserved, including rights for text and data mining and training of artificial intelligence technologies or similar technologies.

Indole-3-acetic acid (IAA) was detected in single plant cells (protoplasts), in real-time, using *in situ*-grown core–shell titanium carbide–carbon QANFAs (TiC@C-QANFAs) on biomedical Ti6Al4V foils and wires to create microelectrodes, as depicted in Figure 1 [28]. Even though the sensor measures real-time vesicular release, which is a fundamental physiological insight, no environmental context was described. The LOD of the modified microelectrode was 1 nM and the linear dynamic range was 16 nM–1.0 µM. As it can be observed in Table S2, this is among the lowest LODs for hormones. The lowest LOD for hormones is 17.4 aM, detected with carboxylated graphene oxide–carboxylated multi-walled carbon nanotubes–Fc and PDANPs–antibody on root tips of soybean seedlings [29]. The analyte was, in the latter case, gibberellin (GA3) and plant extracts were used, but with limited stability as it is an antibody-based recognition system.

Other methods to detect electrochemically plant hormones are described in the literature and presented in Table S3: salicylic acid (SA) was detected using self-supporting N-doped graphene microelectrodes, with possible field compatibility and portability [30]; methyl jasmonate was detected using an electrochemical immunosensor in plant extracts (having stress signaling relevance, but uncertain stability because of the antibody usage [31]) or using FeS_2_ on cellulose paper [32]; methyl salicylate (MeSa) was detected using a tri-enzyme sensor [33].

### 5.4. Ions/Heavy Metals

Nitrate is one of the primary nitrogen sources for most crops, and a central regulator of plant growth. After the uptake from the soil, it is transported through the xylem and reduced, to become a building block for the synthesis of amino acids, proteins, nucleic acids, and chlorophyll. Its concentration reflects both soil nitrogen availability and plant uptake efficiency. Nitrite is not normally found in high concentrations in healthy plants. Distinguishing between the two is very important, as one is a fertilizer in optimal concentrations, and the other one is a toxic component. Both are signaling molecules in plants. Nitrate is not normally electrochemically active, as opposite to nitrite. In this sense, nitrate is usually detected using organic electrochemical transistors (OECTs), rather than classical electrochemical methods described in most of the cases along this review [34].

Previous methods to detect ions transport in plants are destructive or use radiolabeling methods [4]. Nitrite was detected using PdNi/PdCo nanoparticle sensors, in buffered solutions of pickled cabbage and bamboo shoot sour juice [35], but with low–moderate field relevance, as no *in planta* studies were performed, and with susceptibility to interference from plant sap components.

Various gases (H_2_, H_2_S, NH_3_, NO_2_, and VOCs) were detected using noble metal (palladium, platinum, and gold) ion-chelated DNA/single-walled carbon nanotubes (SWCNTs) with chemoresistive gas sensors [36,37], but the sensors have no proven field-deployable relevance.

Even though potassium ions (K^+^) were described as being detected using wearable sensors on plant leaves, the extraction of ions is necessary, and it is performed in liquid buffers contained in a tube, in which the leaf is inserted, not being suitable for long-term monitoring [38]. The electrochemical sensors composed of a K^+^ selective membrane and other PVP and PEDOT:PSS membranes are a valid tool.

The usual perception of heavy metals is that they are toxic to living organisms, but some of them (such as Fe, Zn, Cu, Mn, and Ni) are micronutrients for plants and are essential for plant growth, photosynthesis, respiration, etc. The toxic ones (Cd, Pb, Hg, As, and Sb), interfere with nutrient uptake, damage membranes, proteins and DNA, induce oxidative stress, and suppress photosynthesis and root development. Electrochemical sensors can contribute to the correlation of stress biomarkers with heavy metal exposure levels, early stress detection, and data-driven decisions for soil remediation and crop management. Different heavy metals were also investigated: copper and its mechanism in embolism removal in xylem vessels is an interesting microfluidic study [39]. Cadmium, copper and lead were detected using MIPs (imprinted nanowire-modified PGE) in biological extracts of plant edible products [40,41], but no *in planta* or real-time deployment approach is presented; however, the approach could be used for soil–plant contamination screening. Cadmium was also detected using a 3D graphene sensor for the uptake in rice plants (bacterial cell and rice tissues), but is still a laboratory-based study [41]. Antimony (III) was detected in edible plants [42], without real-time or *in vivo* monitoring, and this is more likely useful for regulatory compliance, not crop management.

### 5.5. Pathogens

Plant disease assessment can also be performed using electrochemical methods, through the identification of pathogens (one of the most studied categories of important molecules in plant health). Pathogens (such as viruses, bacteria, fungi, and oomycetes) are among the most critical threats to global agriculture, with significant crop yield losses, reduced crop quality, and economic damage. Early detection of pathogens is an important milestone for sustainable crop production, food security, and, in the end, smart and precise agriculture.

**Figure 2:**
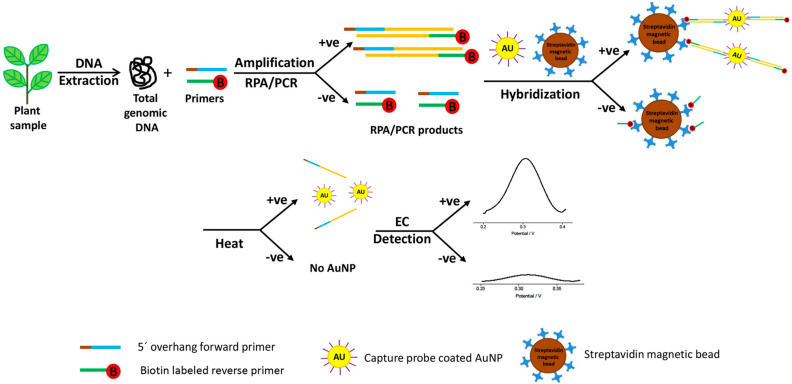
The process of electrochemical bioassay for plant pathogen DNA detection as presented in [43]. Reprinted with kind permission from reference [43]; licensed under CC BY 4.0. No changes were made. Copyright © 2017, the author(s), (http://creativecommons.org/licenses/by/4.0/).

Lau et al. described a reversed biotin-based primer for a target pathogen DNA sequence (*Pseudomonas syringae*) amplified with recombinase polymerase amplification (RPA) that was selectively bound to streptavidin-modified magnetic beads. The capture probe consisted in a thiol functional group (-SH) oligonucleotide that bound selectively to colloidal gold nanoparticles (AuNPs, which enrich the screen-printed carbon electrodes, SPCEs) [43]. The streptavidin magnetic beads were separated with a magnetic plate, washed and heated to release any bound AuNPs into solution; the solution was further analyzed with differential pulse voltammetry (DPV). The control samples (healthy plants, *Arabidopsis thaliana*) did not contain any AuNPs after the heating, and did not exhibit enriched electrochemical signals, as opposed to the infected plants (Figure 2). The work supports rapid molecular diagnostics, with low-energy consumption and early-stage detection before visual symptoms, but not in a plant-wearable approach or realistic field-deployable manner, as plant extracts are performed in the initial steps for the DNA extraction of the pathogen.

P-ethylguaiacol is one of the fungal infection markers and a fingerprint compound of the volatile signature (VOC) of plants infected with fungus. Nanoparticles of TiO_2_ or SnO_2_ on screen-printed (SP) carbon electrodes were used as nanomaterials for the detection of p-ethylguaiacol (VOC) [44]. Even though this one of the most field-ready stress/pathogen indicators existing, it still needs potassium hydrogen phthalate electrolyte added in a closed, oxygen-free electrochemical cell and the authors only tested the sensors using a simulated sample study using typical chemicals released during *P. cactorum* infection of plants.

Other pathogens were detected in plant-related samples: *Agrobacterium tumefaciens* in biological models, but not in plants [45], *Watermelon mosaic virus* for pre-symptomatic detection, with laboratory-based DNA amplification [46], *Aristolochia* and *Asarum sieboldii sap* extracted from cotton leaves, with the help of robust CNT–CuNP nanomaterials, but with laboratory DNA amplification [46,47], cauliflower mosaic virus 35S gene, with self-doped polyaniline-DNA hybrid, but without direct tests related to plants [48], nucleic acid of *Citrus tristeza virus* (plant virus), with a screen-printed carbon electrode (SPCE) + electrodeposited gold nanoparticles (AuNPs), immobilizing thiolated ssDNA probes, without amplification, but with pathogen spikes in citrus plant extracts [49], *Ganoderma boninense*, with DNA-free methods, based on quinoline biomarker detection [50], and *Listeria monocytogenes* in wild blueberries samples, with gold-modified electrodes, but without proof of efficient in-field plant monitoring [51]. Other pathogens were detected using similar approaches: *Agrobacterium tumefaciens* [52], volatile organic compounds for *Botrytis cinerea* [53], *Ralstonia solanacearum* [54,55], agroviruses [47], *Bean pod mottle virus* (BPMV) [56], tobamoviral [57], *Pseudomonas aeruginosa* infection [58], aflatoxin AFB1 [59] and mycotoxin [60].

### 5.6. Reactive Oxygen/Nitrogen Species (ROS/RNS)

Different abiotic stresses (drought, salt, heat, gamma irradiation, heavy metals, etc.), as well as biotic stress (pathogens, viruses, bacteria, pests, etc.) can inhibit different metabolic processes in plants, and as a consequence, lead to accumulation of reactive oxygen species (ROS) in plants [15]. ROS can act as redox signaling molecules but, in some cases, could also involve oxidative damage during the oxidative burst (due to various extreme conditions, both abiotic and biotic stressors). ROS molecules include the following: superoxide (O_2_^−^), hydrogen peroxide (H_2_O_2_), hydroxyl radical (·OH), singlet oxygen (O_2_^−^), peroxynitrite (ONOO^−^), nitric oxide (NO), nitrogen dioxide (NO_2_), nitrite ion (NO_2_^−^), and hyponitric acid (HNO), with some of them also being reactive nitrogen species (RNS). Their concentrations and spatiotemporal dynamics provide some of the earliest and most sensitive indicators of plant health status.

ROS/RNS have a dual role in plants: signaling vs. damage. In low concentrations, they regulate as signaling molecules for hormone synthesis, stomatal closure, defense gene activation, etc. At high or sustained levels, they exert lipid peroxidation, protein and DNA damage, growth inhibition and cell death. Adaptative response and pathological stress can be determined by monitoring ROS/RNS concentrations. There is no better option for detecting early stress in plants than directly detecting ROS/RNS in plants.

**Figure 3:**
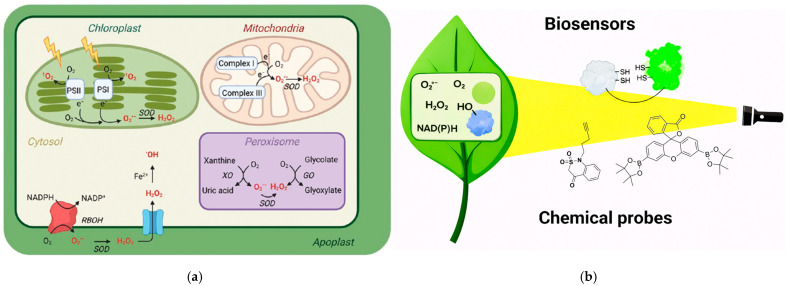
(**a**) Schematic of ROS pathways in plant cells. (**b**) Graphical representation of the chemical fluorescent probes used to detect ROS. Reproduced from reference [62], https://pubs.rsc.org/en/content/articlelanding/2021/cb/d1cb00071c, with permission from the Royal Society of Chemistry and © 2021 The Author(s). Published by the Royal Society of Chemistry. Licensed under CC BY 3.0. No changes were made. (https://creativecommons.org/licenses/by/3.0/).

In general, for ROS detection in plants, electrochemical detection (quantitative assessment) is performed in comparison with fluorescent staining for qualitative and sometimes quantitative assessment. The fluorescent methods are not suitable for *in situ* and *in vivo* real-time monitoring, being highly specialized methods, with experts and expensive optical spectrometric equipment needed [61]. Most of the biosensors are also related to fluorescent biosensors, especially for H_2_O_2_ (Figure 3) [62]. However, most fluorescent methods are not suitable for portability, field measurements, or non-destructive evaluation of ROS content, and the specificity for specific ROS entities is quite scarce (as usually other ROS will also react with a fluorescent molecule).

Soil salinization is an important issue in agriculture, as 20% of irrigated soil world-wide is affected by this. A multi-walled carbon nanotube-titanium carbide–palladium composite (MWCNT-Ti_3_C_2_Tx-Pd) was used to detect hydrogen peroxide in *Arabidopsis thaliana*, using electroreduction at 0.0 V, with a limit of detection of 3.83 µM. The detection was performed on leaves grown under different salt stress [15]. The plants were grown under normal conditions for 27 days, and after, were treated with 100 mM NaCl for another 7 days. The leaves were added to 5 mL of 0.1 M PBS, pH 7.4 solution, and studied with DPV. The method was compared with fluorescent staining with DAB. Even though the work presents a direct plant-derived signal, the insertion of leaves in buffered solution is not a sustainable solution for real-time, long-term monitoring of plant health.

Glassy carbon electrodes were modified with hemoglobin–chitosan/graphene–hexadecyltrimethylammonium bromide (Hb–CS/GR–CTAB) for the detection of NO [63]. The inter and intra RSD values for the detection of NO at −0.7 V with the modified electrodes were 4.7% and 5.2%, at 0.50 µM NO. The detection was possible due to the electrocatalytic reduction of NO by hemoglobin, and the enlarged electrode area and more facile electron transfer due to graphene. The surfactant was used to effectively disperse graphene, and chitosan for the dispersion of hemoglobin in the gel, creating a stable composite and also providing the protection of the protein’s natural conformation. The nanomatrix presented pores that contributed to the high surface area of the catalyst. Even though the method offers temporal resolution for the NO detection, oilseed rape leaf homogenate samples were used to spike NO, and thus the measurement was not performed directly *in planta*.

**Figure 4:**
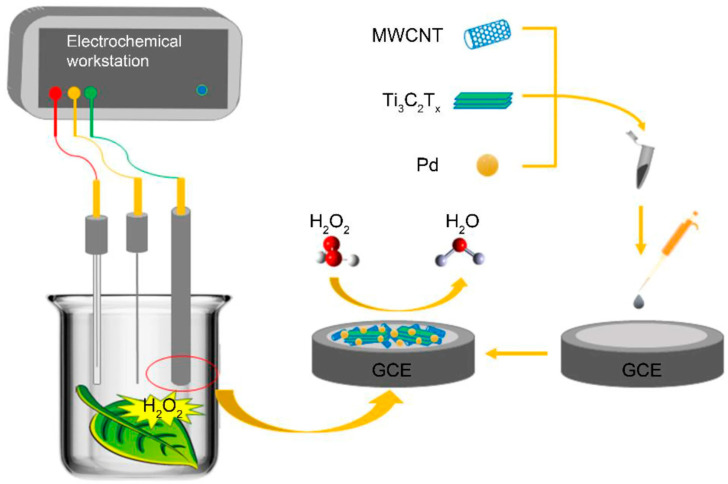
Schematic illustration of structure and working principle of the electrochemical sensor. Reprinted from reference [15] with kind permission from the authors. Reproduced with permission from authors. Copyright: © 2022 by the authors. Licensee MDPI, Basel, Switzerland. This article is an open access article distributed under the terms and conditions of the Creative Commons Attribution (CC BY) license (https://creativecommons.org/licenses/by/4.0/).

Other possible approaches include mesh nanoelectronics detection of ROS in the extracellular space, in response to stress, with milliseconds temporal resolution [8], multi-walled carbon nanotube-titanium carbide–palladium (MWCNT-Ti_3_C_2_Tx-Pd) for assessment of salt stress (Figure 4, [15]), paper-based sensors modified with AuNPs/MoS_2_ paper and PtNPs/MoS_2_ paper, but only in plant extract of aloes, which are able to secret H_2_O_2_ in extreme environments [64] or H_2_O_2_ production induced by root-inoculated endophytic bacteria in *Agave tequilana* leaves [65].

Detecting hydrogen peroxide seems to be the authors’ choice when it comes to ROS, but it needs to be established whether this choice is based on an easier methodological approach, or is based on its real biological importance. The field detection of ROS/RNS in plants still needs further exploration, together with establishing which ROS plays which role in the redox signaling of plants and when the ROS appear. Other ROS, such as peroxynitrite, could also be detected using electrochemical sensors based on cobalt phthalocyanine catalyst [66], but these methods need adaptation for plant-related samples. Multiplexing sensors to detect multiple ROS at the same time might be the next area of focus for future study.

### 5.7. Antioxidants

Antioxidants, as their name suggests, are the counterbalance central regulators in plants that combat ROS/RNS. Except for the mitigation of oxidative stress, they increase stress tolerance and adaptation, they act in the defense against pathogens and pests, and they influence growth, development and quality traits. Some antioxidants are also biomarkers for different biotic stress factors.

Chlorogenic acid (CGA) was detected using a modified GCE with a functional platform by grafting vinyltrimethoxysilane (VTMS) in multi-walled carbon nanotubes (MWCNTs) and covered by a molecularly imprinted siloxane (MIS) film prepared using the sol–gel process [67]. CNTs are well known to contribute to charge transfer enhancement, high electroactive surface area, low charge transfer resistance, and the possibility of functionalization. As a control, non-imprinted siloxane (NIS) was used, meaning CGA was not added in the molecularly imprinting process. The MWCNTs-VTMS was introduced to enhance the specific surface area of the further imprinted siloxane electrode. Similar to enzyme active binding cavities, functional monomers in the siloxane matrix are responsible for creating specific chemical functions in the binding cavities of MIS, so that the rebinding of the target molecule becomes favorable. Selective interactions between CGA and MIS (recognition element) increase the sensitivity and selectivity of the electrode, as the value of RSD is only 2.5%. Even though the sensors measure phenolic antioxidant accumulation, they were only validated in food matrices, required sample extraction and cannot work in a continuous system. They are best suited for precision phenotyping and quality assessment, not live field sensing.

Luteolin was detected in peanut hulls and Perilla using boron nitride nanosheets (BNNS) loaded with AuNPs deposited on a GCE [68]. BNNS have a high electrocatalytic activity towards the oxidation of luteolin. The loading of AuNPs on the BNNS platform, which exposes sufficient electrocatalytic sites for luteolin oxidation to take place, offers superior electrochemical performances, compared to other luteolin sensors described in the literature. The composite lowered the overpotential of luteolin oxidation (to approx. 0.2 V). Very good RSD values were obtained for multiple luteolin measurements (3.6%). This is valuable for laboratory-supported precision agriculture, but there was no demonstration of field deployment.

Gallic acid was detected in plant-based beverages (such as green tea, red wine, grape juice and pomegranate juice) [69] on a MWCNT/graphene/GCE platform, at the optimal pH value of 6, using DPV. Rutin was detected in orange juice, with extract base analysis, not really adaptable for the wearable approach [70], and in *Arrabidaea brachypoda* extract, also not field-deployable in its current form [71], using cyclodextrin-functionalized MWCNTs on GCE. Other electrochemical methods to detect antioxidants in plant-related samples have been developed, but with similar limitations: quercetin, with no portability demonstrated [72]; antioxidant activity of metabolites, but not *in vivo* [73]; gallic acid, with too complex of a method for the field [74] or in medicinal plants, for quality control more than for agriculture [75]; amygdalin, with destructive methods [76]; tert-butylhydroquinone, butylated hydroxyanisole and phenol, for post-harvest quality control [77]; luteolin, based on extraction methods [78]; antioxidant activity of metabolites, but not *in vivo* [73]; quercetin, without a field-deployable demonstration [72,79,80]; rutin for the detection of phenolics in wine [81], in natural vegetation [82] or in other real samples [83]—being better for ecological monitoring than agriculture; neuroprotective hibifolin [84]; salvianic acid A, with pharmaceutical application, not plant health monitoring [85]; sinapic acid and syringic acid, with more medicinal application than agricultural application [81]; and tannic acid, with an extract-based method [86].

### 5.8. Alkaloids

Alkaloids are an important class of plant biomolecules that could act as strong illegal drugs. Differentiation between them and their quantification could make a real difference from both scientific and legal points of view. THC level and total cannabinoid content in *Cannabis sativa* L. was determined using both a carbon black-modified screen-printed electrode (SPE-CB) and PEDOT-modified SPE [87], work that is valuable for the precision cultivation of such plants and offering quality control for the content of several crops, which may be illegal (if the content of the THC level is too high) or legal (if the THC content is low enough). Thus, this approach is more relevant for precision crop management (e.g., THC content in hemp cultivation remains in the imposed legal range, for cultivation of industrial hemp [88]) than for plant health sensing. The LODs were around 30–45 µM depending on the chosen alkaloid (Δ9-THCA or CBDA) and distinguishing between these two is very important from a legal and biologically induced symptoms point of view. Theobromine is the alkaloid in cacao plants that provides the bitter taste of chocolate, and has also been detected in green tea, chocolate and coffee extracts [89]. Theobromine was detected using carboxyl-functionalized multi-walled carbon nanotubes (fMWCNTs) and soluble carboxymethylcellulose (CMC) on a glassy carbon electrode (GCE) in the green tea, chocolate, and coffee extracts. Both studies are more applicable to quality control than to smart agriculture plant health monitoring, as no *in planta* or *in vivo* studies were performed.

### 5.9. Phytochemicals

**Figure 5:**
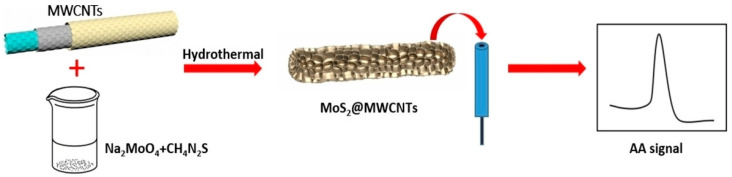
Synthetic routes of MoS_2_@MWCNT modified GCE. Reprinted from reference Wang, Y.; Mamat, X.; Li, Y.T.; Hu, X.; Wang, P.; Dong, Y.M.; Hu, G.Z. Glassy Carbon Electrode Modified via Molybdenum Disulfide Decorated Multiwalled Carbon Nanotubes for Sensitive Voltammetric Detection of Aristolochic Acids. Electroanalysis 2019, 31, 1390–1400 [91], with kind permission from WILEY. Copyright © 2019 WILEY-VCH Verlag GmbH & Co. KGaA, Weinheim. All rights reserved, including rights for text and data mining and training of artificial intelligence technologies or similar technologies.

Tryptophan (Trp) is essential in IAA (auxin) biosynthesis as well as regulating plant growth. Trp was detected in tomato fruits and juice using polydopamine/graphene/MnO_2_ glassy carbon-modified electrodes (PDA/RGO-MnO_2_/GCE), using CA at 0.75 V (vs. SCE), 0.1 M PBS, pH 4.0–7.0 [90]. The authors explain that the developed sensor is not suitable for the *in situ* monitoring of small plants and small tissues owing to its large size, but it has been successfully applied to tomato plants and tomato juice, through the insertion of the macroelectrodes into the tomato fruits, creating damage to the fruits. The sensors are promising for plant physiological monitoring, but are not yet field-ready; however, this work is one of the most important regarding the correlation of plant health and phytochemicals. Aristolochic acids were also studied in plants using similar materials based on MoS_2_ nanosheets grown on bowl-shaped hollow carbon spheres and MoS_2_ on a glassy carbon electrode (MoS_2_-GCE) and the synthetic routes are represented in Figure 5 [91,92]. Nevertheless, the studies performed for aristolochic acids are relevant for herbal medicine safety and were done using extracts of traditional herbs, but are not suitable for agriculture. Cynarin was also studied in plants [93] using electro-synthesized functionalized-polybithiophene/MWCNT/GNP, and this could be relevant for nutraceutical profiling, but not for real-time plant monitoring.

### 5.10. Pesticides/Herbicides/Fungicide/Insecticides/Pollutants

Most of the pesticides and other potentially hazardous plant treatments (that are created for plant health), need to be better monitored, as they can do more damage in the long term, than help plant growth, if misused or mis-dosed (as every substance taken in by an organism). Regulatory laws have started to forbid the use of some of these substances, as they can destroy biodiversity and pollute the environment. Chromatographic techniques are the most used methods, but they are not suitable for real-time monitoring *in situ* and *in vivo*.

Thiamethoxam monitoring in living plants was performed using sensitive chitosan-stabilized silver nanoparticle electrode (CHI-AgNPs/CPE) detection directly on *Zea mays* and *Phaseolus vulgaris* extracted tissues [94]. The sensors are important for this category, but plant extraction is still needed.

Other methods were described for the herbicide triclopyr, with extraction from tomatoes [95]; glyphosate from untreated rye juice [96]; mesotrione in extracts of corn [97]; imidacloprid (IDP) in extracts of brown rice redissolved in 0.1 M PBS or in extracts from thyme and guava leaves [98,99]; diazinon in food samples related to plants [100]; carbaryl [101]; cyprodinil in food samples related to plants [102]; pesticides in aerosol phase [103].

### 5.11. DNA/microRNA

The microRNA extracted from *Arabidopsis thaliana* was detected using AuNPS-coated Bi_2_S_3_ deposited on indium tin oxide (ITO) slides [104]. Hairpin DNA was used as an immobilization probe for the microRNA, and after the immobilization, streptavidin was added to obtain streptavidin/microRNA/probe/AuNPs/Bi_2_S_3_/ITO. The AuNPs were used to immobilize the hairpin DNA probe through Au-S specific bonding. Streptavidin was immobilized through biotin-specific interactions (the hairpin DNA was modified with biotin) and it acted as a photocurrent inhibition molecule. The bismuth sulfide was used due to its excellent photoelectric conversion material, and the decrease in photocurrent can be correlated with the concentrations of microRNA. Ascorbic acid, negatively charged at pH 7.4, was added as an electron donor during the detection process. The method was sensitive, with an impressive detection limit of 3.5 fM and a good RSD value (6.68%). Abscisic acid (ABA), which acts as a phytohormone, was used to up-regulate the formation of microRNA; when compared to untreated *Arabidopsis thaliana* seeds, this proves that important plant hormones for plant growth could be studied using the method. Unfortunately, this work does not present direct detection in plants, requires laboratory-performed RNA extraction and cannot be deployed to the field.

Another method that studies the effect of phytohormones (such as ABA) on microRNA expression detection consists of a similar strategy, but using mimic enzyme catalysis and a carboxylic graphene–hemin hybrid nanosheet. AuNPs were first deposited on the GCE, followed by single-stranded DNA S1 and mercaptopropionic acid, MPA (for removing non-specific interactions), followed by the target microRNA [105]. The ssDNA was hybridized with bio-barcoding ssDNA for the introduction of -NH_2_ groups on the surface of the electrode, which will further create an amide bond with the -COOH groups on the carboxylic graphene-hemin hybrid, to form the final graphene–hemin/barcode/microRNA/ssDNA/AuNPs/GCE immunosensor. The amount of target microRNA can be quantified by monitoring the microRNA hybridization event, which will control the quantification of the further electro-reduction hemin-based catalysis of the benzoquinone oxidation product obtained in the presence of H_2_O_2_. As in the previous work, the ABA has an up-regulation effect on the microRNA expression for the treated *Arabidopsis thaliana* seeds. The results were compared with qRT-PCR, and the method was found to be reliable enough to be applied in real samples, but the work is not performed *in planta* and it requires several steps to be performed in a laboratory. The detection of rye DNA was performed for DNA damage assessed post-exposure to glyphosate, but the complex workflows make the process not deployable to the field [96]. Other DNA detection methods are described in references [106,107], but the studies assess pharmacological potential for medicinal screening, rather than for agriculture. Phosphinothricin acetyltransferase gene sequence was also detected on a polyaniline-(mesoporous nanozirconia)/poly-tyrosine film [108].

### 5.12. Proteins/Nucleotides/Amino Acids

L-glutamate was detected in both cucumber juice and cucumber fruit. The sensor consisted in L-glutamate oxidase drop-casted over electrodeposited platinum nanoparticles, followed by electrodeposited poly-m-phenylenediamine on a graphite rod electrode (GluOx/PMPD/Pt/GRE). GluOx, in the presence of oxygen, transforms glutamate into α -ketoglutarate, ammonia and hydrogen peroxide (which is detected further using chronoamperometry at 0.4 V). Even though the electrode *in vivo* monitoring was performed by inserting the mini-type glutamate sensor into the cucumber fruit, the concentrations of L-Glu were spiked using injections of the cucumber at different concentrations [109]. So, even though these sensors could be a strong candidate for smart plant sensing, the authors still need to demonstrate that the sensor is able to detect the endogenous metabolite produced directly by the plants.

Carbon nanotubes and graphene-modified screen-printed carbon electrodes were used to detect phytochelatins (oligomers of glutathione) in *Hordeum vulgare* and Glycine max treated with Hg(II) or Cd(II) using HPLC and CA at 1.0 V vs. Ag/AgCl, but the device is not portable, cannot perform real-time operations and was only validated on extracted samples [23].

### 5.13. Study of Plants—Phylogenetic and Species Studies Through Elecrochemical Fingerprinting

Different plant extracts from the same family of flowering plants or different species of plants were already identified using electrochemical sensors. This was performed using plant tissues immobilized on the surface of the electrode and voltammetry of immobilized nanoparticles [110]. Electrochemical fingerprints are obtained by measuring entire electrochemical profiles, but the method is good for taxonomy, not for environmental monitoring.

Plant leaf tissues were studied using screen-printed carbon electrodes coated with a thin layer of polydopamine-functionalized graphene sheets, for studying the fingerprints between different species [111]. As genetic relationships determine biochemical and physiological characteristics in plants, the statistical analysis of the characteristic is performed, rather than the quantification. The electrochemical active compounds (such as flavonols, phenolic acids, procyanidins, alkaloids, and pigments) from the plant tissue will construct a profile, representing the fingerprint that helps you distinguish between different species through DPV scanning. Known molecular phylogenetics (which is the study that includes both the evolutionary history and relationships among or within groups of organisms using DNA sequencing) were compared with the information obtained from electrochemistry, using three different buffers, on 19 species of Amaryllidaceae. The authors concluded that pattern recognition modes, constructed from the electrochemical fingerprints of the different species, gave a persuasive taxonomical result in comparison with the molecular studies [111]. Phylogenetics can be assessed using this method, but as no dynamic physiological information is presented, it is not suitable for agriculture sensing.

Species identification in *Malvaceae* leaves was performed using extractions with water from 16 species, and further dilutions in 0.1 M PBS, for plant identification only, using glassy carbon electrodes as working electrodes [112]. A similar approach was used to identify 10 ornamental plants [113]. Stringent response [114], phylogenetic investigation [115] and electrochemical profiling of plants [116] were also studied using electrochemical biosensors. These kinds of studies have no obvious field relevance.

### 5.14. The Study of Taste

Robotic chemical sensors are widely discarded as analytical tools due to the encountered challenges. Identifying the solutions to present robotic chemical sensor bottlenecks requires wearable chemical sensor technology and flexible electronics, as presented by Ciui et al. [117]. The authors performed both liquid and solid analysis for food samples taste (sweetness, sourness, and spiciness), via direct electrochemical detection of glucose, ascorbic acid, and capsaicin. For the solid analysis, agarose hydrogel (0.5% agarose powder was added to 0.1 M PBS (pH 7.4) under specific conditions), was used to complete an electrochemical cell during assays of solid food samples (such as green pepper, black pepper, wheat flour, and curry powder). The powders were left to diffuse through the gel and analyzed after a specific time [117]. Even though this work has relevance for food robotics, it has no relevance for agriculture. Edible oils were also studied using MIPs [118].

## 6. Wearable and Implantable Electrochemical Sensors for Plants

A special section is presented in the current work, related to wearable and implantable electrochemical sensors, as these types of electrochemical (bio)sensors are essential for real-time monitoring and for rapid decision making related to farming operations, as required to mitigate crop loss, and they have potential for integration into precision and smart agriculture (and thus are field deployable). In 2023, the “World Economic Forum” placed wearable plant biosensors in the top 10 emerging technologies and the field of wearable sensors in the fifth place. These kinds of sensors could be integrated into the Internet of Things for agriculture (IoT, as for smart clothes or smart houses/buildings). The consumption of resources, such as water, energy or plant treatment, can be optimized in a sustainable agriculture system, whereas rapid data analysis of physical and chemical traits can be obtained remotely from portable small devices. The performance of the existing wearable and implantable electrochemical sensors for plants are presented in Table 1, and the materials of those sensors are described in the present section. As can be observed, this field of wearable and implantable electrochemical sensors applied to plants is an emerging field, as the first manuscripts were published in 2013 and most of the technologies were developed after 2020 (independent of the nature of the analyte). Common analytes or characteristics that were examined with wearable or implantable electrochemical sensors were mostly plant hormones (13 scientific articles), pesticides and fungicides (4 scientific articles), hydration and ionic strength (5 scientific articles), and reactive oxygen species—ROS (4 scientific articles), followed by only 1–2 scientific articles for saccharides, ions, synthetic plant hormones, heavy metals and VOCs. The plants to which they were applied were usually skins of vegetables (tomatoes, apples) or plant leaves.

Operational performance of the sensors in field conditions should consider, but not be limited to, the following indicators: temperature tolerance, pH tolerance, ionic strength tolerance, mechanical durability, anti-fouling behavior, possibility of functioning in a continuous *real-time* and *in situ* monitoring system, with early stress detection, etc. In terms of smart and precision agriculture and wearable/implantable electrochemical sensors for plants, long-term stability is more important than ultra-high sensitivity, and lab-level ultra-low LODs are less valuable than reliable detection within physiological concentration ranges. Sensors as field-deployable decision tools for precision agriculture must demonstrate robustness, selectivity and stability under these fluctuating conditions, storage stability, low calibration frequency, and low signal drift over the deployable period, as well as an appropriate operational lifetime. The real environmental factors important for wearable/implantable sensors are also underlined for both wearable and implantable sensors.

According to Table 1, the “Field-relevance” column categorizes the sensors in qualitative, but defensible levels (Low–Moderate/Moderate/Moderate–High/High). This differentiates between sensors that have been validated on plants, under abiotic or biotic stress/in realistic conditions and the ones that remain at the proof-of-concept level, but are still applied directly on plants. This classification takes into consideration in-field operation, real plant matrices and actionable plant health information (stress, nutrients, pathogens, and residues).

### 6.1. Wearable Electrochemical Sensors

Usually, wearable electrodes are flexible and have a semi-solid electrolyte to adhere to the irregular surface of the fruits or leaves (or other plant parts) and gather *in situ* and *in vivo* measurements (in this case, in field crops). Theoretically, some of the above strategies could be adapted to field-deployable devices, but the authors studied and expressed only limited characterization for real-time monitoring directly on plants (under realistic conditions) with their developed sensors; herein, we summarize the ones that were already tested, validated or implemented for this explicit purpose: improved strategies for plant heath monitoring, plant/plant treatment screenings, or other similar purposes.

Graphene-related materials (GRMs) are flexible, one-atom-thick single or multiple layers, and can increase electrochemical performance. GRMs are used in creating wearable sensors. One example is laser-induced graphene (LIG) transferred on polydimethylsiloxane (PDMS) and modified with Nafion and organophosphorus hydrolase (OPH), for detection of methyl parathion pesticide. The electrochemical current was enhanced with AuNPs modification. The wearable sensor transmits data to a smartphone device wirelessly, for real-time and *in situ* electrochemical analysis of pesticides on the surface of agricultural products [119]. The pre-sprayed leaves of spinach and apple fruits were studied with this technology, using a gelatin semi-solid electrolyte and the internal stickiness of the device. PDMS is a non-toxic, flexible and optically transparent material, often used in soft lithography, but it is non-biodegradable. As real environmental performance indicators for this laser-induced graphene technology, we should consider that the system operates on non-flat, contaminated plant surfaces, tolerates ambient humidity and surface waxes, can be used for detection under real agricultural residue conditions, has a response time <1 min, is non-destructive and removable, and does not require a liquid extraction method for the analytes, while also offering *in situ* pesticide residue monitoring directly on crops under realistic surface conditions. These real environmental indicators make this multimodal sensor highly relevant for field measurement, even though no direct biochemical sensing is performed.

A more eco-friendly and sustainable material for sensor printing is cellulose acetate (CA), which is biodegradable, but still flexible. Using commercial carbon ink (which is more sustainable than other inks), it was used to detect carbendazim and paraquat (pesticides) [120]. CA can be obtained from unused plant biomass or agricultural waste. The authors prove how flexible paper-based sensors can be created and how they can be successfully used, but the carbon electrode surfaces were not further functionalized to give more specificity and sensitivity to the sensor. Only a simple pretreatment was applied to remove non-conductive substances from the ink, but this might not be enough as the detection potentials are in the range where many other molecules will interfere (+0.55 V and −0.87 V). For this purpose or other analytes, other analytical methods presented in this review could be adapted. The authors added PBS (which is not as practical as using semi-solid electrolytes, as presented above) for the lettuce and tomato skin analysis (*ex situ,* spraying and drying solutions of pesticide on the vegetables). Nevertheless, the biodegradable CA sensors have similar limits of detection (tens of nanomolar) to the PDMS/LIG sensors for the same category of analytes (pesticides), operate in ambient environmental conditions, and are disposable, low cost and rapid (<60 s). Similarly, and also in the category of Moderate–High relevance for field deployment, are the screen-printed sensors made of PE (polyester) and PLA (lactic acid), tested on apple and cabbage skins [121]; these work under variable pH and humidity and are robust, but are short-term solutions (as they also use buffered solutions dropped on the surface of the sensors). In the category of Moderate relevance is the detection of carbendazim on the skin of apple and cabbage [122].

A wireless portable device has been used for the detection of α-naphthalene acetic acid, a synthetic auxin widely used as a plant growth regulator [123]. The sensor is composed of a phosphorene/Ti_3_C_2_-MXene nanohybrid with high ambient stability on laser-induced porous graphene as a nanozyme flexible electrode. MXene is a new type of 2D material, with high surface area, enhanced electron transfer and improved antifouling coatings. As a field-ready agrochemical monitoring sensor, it is one of the best plant-wearable sensors to date, not because it analyzes complex plant physiology, but because it targets controllable agrochemical input which directly supports the practices of smart and precision agriculture.

**Figure 6:**
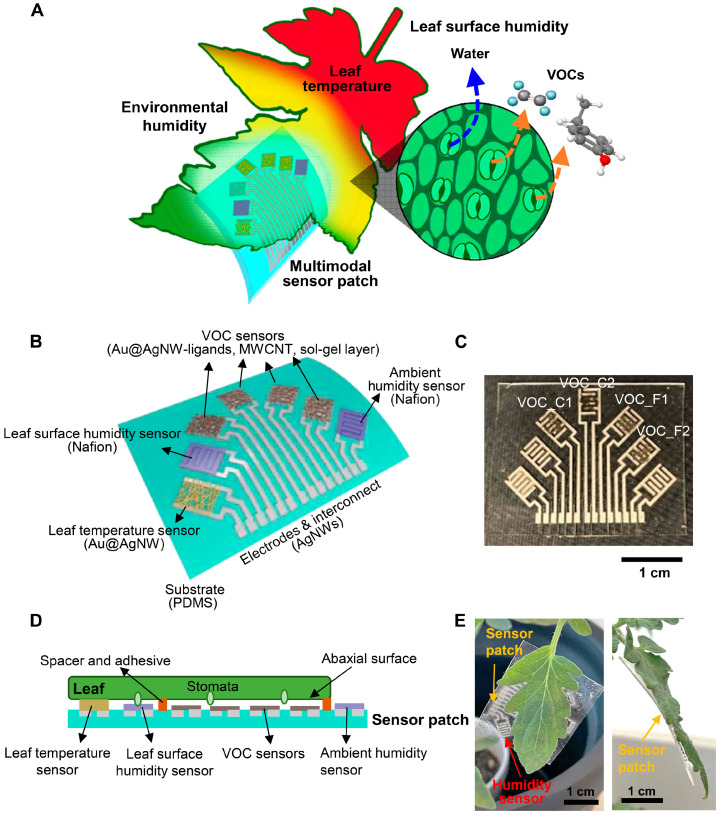
Multimodal wearable plant sensor. (**A**) Schematic illustration of the sensor attached to a plant leaf. Our multimodal sensor is attached to the abaxial leaf surface to simultaneously monitor various physiology data from the leaf. Blue and orange arrows represent emissions of water and VOCs through stomata, respectively. Different colors of the leaf represent the variation in leaf surface temperature. (**B**) Overview of the wearable sensor design, which consists of four VOC sensors, one leaf surface relative humidity sensor, one leaf temperature sensor, and one environmental humidity sensor. All seven individual sensors were integrated with AgNW interconnectors on a PDMS substrate. (**C**) Photograph of the actual sensor. VOC sensors with different sensing materials are labeled. (**D**) Side view of the wearable sensor patch. (**E**) Photographs of an actual sensor patch attached to the lower epidermis of the tomato leaf. The environmental humidity sensor (red arrow) is the only sensor mounted outside the leaf surface area in the air near the plant. Reproduced from reference [7] © The authors, some rights reserved; exclusive licensee AAAS. Distributed under a CC BY-NC 4.0 license http://creativecommons.org/licenses/by-nc/4.0/”. Reprinted with permission from AAAS.

A PDMS-based wearable sensor was also used by Lee et al. [7] for the detection of VOCs, and was constructed of Au@AgNWs interconnections and multi-walled carbon nanotubes (MWCNTs) embedded in a hydrophobic sol–gel layer made of methyltrimethoxysilane (MTMS) and tetramethyl orthosilicate (TMOS). The PDMS substrate integrates multiple sensors including ambient humidity, leaf humidity, leaf temperature sensors, together with the VOC sensors. The configuration of the electrodes is presented in Figure 6. The sensor has a High relevance for field measurements because it is directly attached to living plants, operates under real outdoor-like environmental conditions, measures actionable agronomic variables (water stress, microclimate), enables continuous, real-time data, not snapshot measurements, and does not require sampling, extraction, or external reagents. This technology is a complementary solution to all of the methods presented in this review, rather than a competitor, as it does not address any biochemical specificity. This paper also includes machine learning integration for quantitative early detection and prediction of the best sensor combination.

Glucose is a primary metabolite, a signaling molecule, a direct byproduct of photosynthesis, and an essential molecule in biological plant processes. A wearable sandwich barium ferrite magnetic sensor was used to detect glucose (GLC), through integration of reversed iontophoresis glucose extraction [124]. The two parts of the magnets are the cathode and the anode. When attached to leaves and after the application of an iontophoretic small field, the glucose molecules flow from the anode to the cathode, followed by their extraction. A screen-printed electrode is incorporated between agarose hydrogels (protection of the leaf), medical tape, and magnetic holder (of the cathode). The anode has an additional PVA hydrogel scaffold for GLC extraction and does not have a screen-printed electrode (SPE). Glucose oxidase (GOx) is used as enzyme to modify the surface of the SPE, for detection of glucose (through classical formation of hydrogen peroxide and its subsequent detection. The method is rapid (<10 min) and it was applied in three plant species for assessing light and temperature stresses on glucose production (photosynthesis), with quantification by chronoamperometry (CA at −0.15 V). Authors compare their work with other GLC electrochemical sensors for plants, and all of them are enzymatic, with similar analytical performances, but different features. The sensor is non-invasive, offers real-time stress monitoring, works across species and has wireless readout.

Perdomo et al. [125] also described similar sensor design for real-time, *in situ*, *in vivo*, and non-invasive monitoring of salicylic acid in avocado plant leaves. The same iontophoretic extraction (as described above) is used for the extraction mechanism of SA. The developed sensors show selectivity towards fructose, sucrose, citric acid, and oxalic acid. These sensors can be used for early stress detection, but are limited by hormone cross-talk complexity.

For the heavy metals, a High-relevance field measurement sensor was developed using a bismuth/Nafion-coated carbon working electrode transducer covered with a polyvinyl alcohol (PVA) membrane for the detection of atmospheric Pb^2+^ [126]. The electrolyte is built in (as PVA acts like an electrolyte-containing collector) and the sensor is a flexible, self-adhesive substrate that can be attached to plant leaves. The High-relevance relates to the self-adhesive screen-printed sensors attached to plant leaves for real-time airborne heavy metal exposure monitoring. Other papers identified in the literature are of Low–Moderate relevance [127], because they used plant extracts to detect heavy metals, as opposed to the High-relevance paper that also used a portable potentiostat to measure the signal and needed no separate sampling in the laboratory.

One of the most studied analyte categories is the phytohormones, as these could be interpreted as signs of early stress detection, but only High-relevance papers are discussed in this section. A nitrogen-doped carbon nanotubes/core-shell Au@Cu_2_O nanoparticles/carbon fiber electrochemical implantable microsensor detected indole-3-acetic acid (IAA) in a living cabbage stem [128]. Core-shell Au@Cu_2_O-graphene-polydopamine (PDA) interdigitated microelectrode array sensor was used to detect salicylic acid (SA) in cucumber leaves [129], in a non-destructive way, and spatially resolved early stress mapping, but with complicated fabrication complexity and scaling challenges.

### 6.2. Implantable Electrochemical Sensors

Implantable electrochemical sensors, on the other hand, are rigid and need high mechanical resistance to be able to penetrate plants. An important aspect in implantable sensors is to study whether the implantation affects the detection and the measuring of the targeted molecules (e.g., the concentration of reactive oxygen species or glucose, which may or may not rise upon physical distress to the plant). The substrate material for sensor development is usually metal-based or carbon fiber-based.

The strongest sensing category is the class of reactive oxygen species (ROS), because it is a direct indicator for early stress determination during an oxidative burst. All of the sensors described in this category are implantable and detect hydrogen peroxide (with the exception of Nafion/Pt, which also detects NO [130]. Carbon fiber ultramicroelectrode + hemoglobin + single-wall carbon nanotubes (Hb/SWCNTs/CFUME, ⌀ = 7 µm) were used in Aloe leaves during saline stress (with 0.3 M, NaCl) [131], with a Moderate–High field relevance, as the method targets early salt-stress detection in living leaves, but in an invasive manner, although physiologically informative. A Nafion/Pt sensor was used on oilseed rape leaves (*Brassica napus*) under drought stress, with High field relevance, because of the long-term *in vivo* monitoring under real drought stress. Gold nanoparticles deposited on indium tin oxide AuNPs/ITO were used in tomato leaves infected with *Botrytis cinerea* [132], within 6 and 24 h post inoculation, with High field relevance, as detection of biotic stress *in situ* is performed during real pathogen infection. Lastly, polyurethane-based microneedles covered by Au + modification with HRP/Cs-rGO biohydrogel (⌀ = 2 µm) were used on tobacco and soybean leaves inoculated with fresh Pto DC3000 culture, 24 h post inoculation [133], and no electrolyte was used for plant measurement. The work has High relevance, being minimally invasiveness, not requiring an external electrolyte, and presenting strong potential for field deployment. Oxidative stress can also be created by mechanical stress applied to plants (such as disruption of the leaves by the implantable needles). Even though this approach is much more accurate than the plant extract methods (where the methods are totally destructive), it still raises the question of whether the detected hydrogen peroxide concentration arises from abiotic or biotic stressors or from the mechanical needle-induced stress. On a positive note, the sap itself becomes the electrolyte of the three-electrode electrochemical system (similar to sweat in humans). Is implantability a design defect for electrochemical ROS sensors applied to plants? This question still needs answering.

Implantable organic electrochemical transistor (OECT) sensors are described for the real-time and *in vivo* detection of glucose and sucrose in xylem sap [134]. The bioelectronic device, which converts biochemical signals to electronic signals, is made of enzymatic biosensors based on organic electrochemical transistors (OECTs). The device was fabricated on 125 µm thick polyethylene naphthalate (PEN), with a Ti/Au source, drain, gate electrodes and wiring. The gate (forming the channels) is modified with poly(3,4-ethyl-enedioxythiophene): poly(styrenesulfonate) (PEDOT:PSS), coated with PtNPs and an enzyme (e.g., glucose oxidase, GOx in a chitosan matrix). PEDOT:PSS increases and modulates capacitance of the gate, PtNPs oxidizes H_2_O_2_ originating from the oxidation of glucose and the enzymes convert sucrose into glucose. Monitoring of sucrose was performed over 24 and 48 h on 8-week-old hybrid aspen trees using the implanted sensors, which were connected to a portable small device (that they designed). They discovered that sucrose concentration in the xylem sap increases during darkness in the vascular tissue of trees (a more qualitative approach, rather than a quantitative measurement). The measurements are more likely qualitative, and not quantitative, but the sensors have long-term stability (>48 h), track diurnal physiological fluctuations, operate inside living plants and are temperature-tolerant. With further modification, these OECT technologies can be implanted to detect other molecules. The authors want to investigate the sensor design further to first enable quantitative *in vivo* sensing and secondly to minimize cork tissue formation for extending the duration of real-time monitoring to several days. A path toward transferable PEDOT:PSS-based capacitive sensors is also described by reference [135].

Salicylic acid (SA) as a plant hormone plays an important role in the induction of plant defense mechanisms against abiotic and biotic stress. Paper-based electroanalytical devices for *in situ* determination of salicylic acid in living tomato leaves were applied using carbon tape coated with a mixture of multi-walled carbon nanotubes and Nafion [136]; these were limited to short-term measurements only and exhibited paper dehydration issues. Other implantable sensors for detection of SA are performed in plant tissue *in vivo*/*in vitro* detection, using self-supporting nitrogen-doped graphene microelectrodes for salicylic acid (SA) [30]. Even though the sensor has demonstrated *in vivo* capability, it also has limited environmental robustness testing. SA was detected on leaves of cucumber seedlings for *in vivo* monitoring, using ratiometric oxidation currents with a Cu metal–organic framework (Cu-MOF) and carbon black (CB)-Nafion composite [10]. The ratiometric method consists of two electroactive molecules: one molecule is the reference molecule (correcting for environment or other factors, in this case Cu-MOFs) and the second one is the one to analyze/detect (in this case, SA). The cucumber seedlings were grown in a salt stressed environment and compared to unstressed plants. For *in vivo* determination of SA, a puncture needle was used to obtain a hole for SA release. The WE of the modified SPE was attached using adhesive tape (or a clip) and TRIS-HCl buffer was added dropwise into the created hole. The levels of SA, using EC sensors, were detected to be 140.55 ± 2.74 µM and 174.06 ± 4.35 µM for unstressed and stressed plants, respectively. The results were compared to UPLC-MS and the RSDs were between 5 and 8%. This ratiometric sensor has only a moderate impact, as even if it were a leaf-mounted sensor, it has a buffer reservoir; it is semi-suitable for field monitoring. Abscisic acid was also studied, being one of the main hormones involved in plant stress responses [137]. Naphthalene was also studied using implantable sensors [123].

Microneedle-based sensors are a particular type of implantable sensor, being less invasive than the usual implantable sensors. State-of-the-art, minimally invasive, multi-mode hormone detection was used to detect salicylic acid (SA) in real-time, in tobacco 5 min post-inoculation with *Botrytis cinerea*, using microneedle-based electrodes with magnetic molecularly imprinted polymers (MIPs) [138]. The sensor can be applied between different species. One important limitation is a complex specialized fabrication of the sensor. Similarly, a minimally invasive amperometric foliar sensor has been developed [139]. The interrogated leaf was sandwiched between the working electrode on one side and the counter and reference electrodes on the other. An implantable and disposable stainless steel (SS)-based electrochemical microsensor modified with Au and Pt nanostructures, reduced graphene oxide (ERGO) and polymerized ST film (PST/Pt-ERGO/Au/a-SS) was used to detect indole-3-acetic acid (IA) in soybean seedlings [140], demonstrating real-time hormone monitoring in living crop plants with minimal invasiveness; direct hormone tracking was linked to growth regulation.

A low-cost electrochemical analysis of foliage stress was performed with the LEAFS platform, by detecting SA hormone as a marker of stress [141]. Porous laser-induced graphene (LIG) material was engraved on Kapton polyimide and the working electrode was further modified with Nafion (sulfonic based ionomer, known for its cation exchange capabilities). Nafion is known for concentrating positive ions and repelling negative ions from the surface of the electrode, restricting the adsorption of interfering molecules, and protecting the surface of the electrode. As LEAFS has an integrated gel electrolyte and a low-cost field analyzer and works without added buffer, it is considered a High-relevance field-deployable sensor. Nafion increased the reproductivity and stability of the electrode [142]. The engraving process was also used to increase electro-catalytic activity for the oxidation of SA. The process is scalable and simple. The LEAFS sensor is different than the other implantable electrodes because it is made on a flexible semi-transparent substrate.

Abscisic acid was detected *in situ* in cucumber plants by direct insertion using an Au@SnO_2_–vertical graphene microneedle array [143], and has High relevance due to the stress-responsive hormone being measured *in situ*, using plant-compatible microneedles. Magnetized microneedles coated with superparamagnetic Fe_3_O_4_ intercalated into a scaffold of multi-walled carbon nanotubes (MWCNTs) were used to detect IAA and SA in tobacco (*N. benthamiana*) and *Arabidopsis thaliana* leaves [139], with High relevance due to its minimally invasive approach, hormone-specificity and plant species independence. Other phytohormones were detected using implantable sensors, including salicylic acid (SA) [144] and salicylic acid (SA) [145].

**Figure 7:**
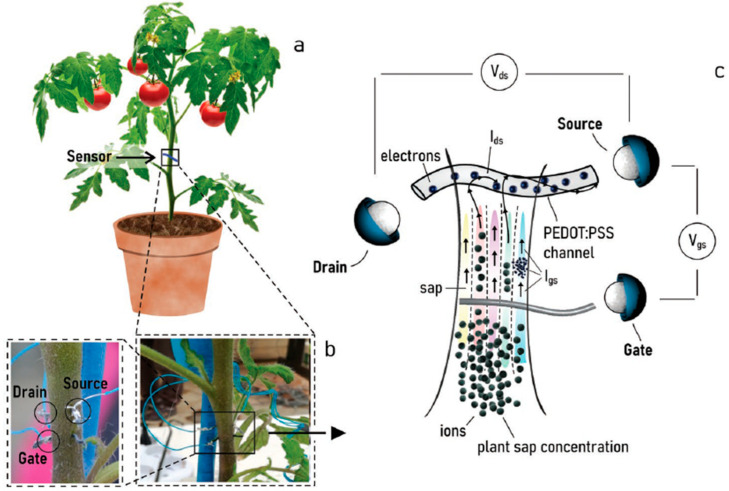
(**a**) Illustration of a tomato plant with the device integrated into the plant stem. (**b**) Detail of the stem showing the biosensor embedded in the plant and the wires connecting the sensor device to an external probe station for acquisition and analysis. (**c**) Schematics of the sensor device: in the plant vasculature, upon application of an external voltage, ions are driven towards the PEDOT:PSS channel, and this generates a measurable current. Reprinted from reference Gentile, F.; Vurro, F.; Janni, M.; Manfredi, R.; Cellini, F.; Petrozza, A.; Zappettini, A.; Coppede, N. A Biomimetic, Biocompatible OECT Sensor for the Real-Time Measurement of Concentration and Saturation of Ions in Plant Sap. Adv. Electron. Mater. 2022, 8, 2200092 [4], with kind permission from WILEY. Copyright © 2022 the authors, under Creative Commons CC BY license, https://creativecommons.org/licenses/. Published by WILEY-VCH Verlag GmbH & Co. KGaA, Weinheim. All rights reserved, including rights for text and data mining and training of artificial intelligence technologies or similar technologies.

The concentration of ions (C) and the ion saturation (S) in plant sap were determined in real-time and simultaneously with an organic electrochemical transistor (OECT), referred to by the authors as a *bioristor* (biosensor + transistor, represented in Figure 7) [4]. A mathematical model was used to correlate system variables with measured currents and with C and S. Two textile electrodes were introduced into the sap of the plant: one electrode acts as the transistor channel (drain-source, metal wire covered with conductive poly(3,4-ethyl-enedioxythiophene): poly(styrenesulfonate), PEDOT: PSS) and the other serves as the gate, with the electrodes bridged by the sap. Upon applying a positive voltage at the gate, cations from the sap are pushed toward the transistor channel (PEDOT^+^:PSS^−^, a macromolecular salt). PEDOT plays a remarkable role when de-doped by the cations (Na^+^, K^+^, Mg^+^, Zn^+^, etc.), as PEDOT^+^ is reduced to its neutral state, removing charge carriers from the matrix and decreasing the conductivity of the transistor channel in a reversible manner. Thus, the electron flow from drain to source is proportional to the cation flow running from the plant to the channel. Tomato plants were subjected to vapor pressure deficit (for 16 days) and drought and salt stress during (for 25 days). This represents one of the strongest examples of a field-oriented electrochemical plant sensor, even though the studies were performed under controlled conditions. With a continuous monitoring design, it captures the dynamic ionic fluctuations directly in living plants, from the xylem sap. Nutrient uptake efficiency (NUE) can be inferred from this type of sensor, together with transpiration-driven transport and early indirect detection of stress (through salinity changes).

Several implantable organic electrochemical transistors (OECT sensors, *bioristors*) have been described in the literature for the determination of hydration and ionic strength, including an *in vivo* sensor to monitor the effect of vapor pressure deficit (VPD) changes in the plant [146], sensors for ionic changes in plant sap with excellent early warning capability even after six weeks of continuous operation [147], sensors for early drought stress detection [148,149], sensors for crop water management [150], and sensors for plant health monitoring and energy harvesting [151]. All of these developed methods use commercial textile threads modified with PEDOT:PSS, and dodecyl benzene sulfonic acid, with or without ethylene glycol. A more complex method was used create a capacitive sensor directly printed on leaves (tattoo-like), enabling multiplex plant health monitoring and energy harvesting, by measuring impedance changes associated with ionic strength and water loss.

Nitrate was detected using photosensitive epoxy bioresin for direct *in planta* sensing by embedding the sensor into plant tissues, operating under natural transpiration and nutrient flow and claiming field durability due to the mechanically robust epoxy resin [34]. The bioresin is composed of an artificial enzyme (vitamin B12), graphene oxide (GO), and a photoresist (SU8). The catalytic analytical activity is performed with the help of the cobalt metal in the vitamin B_12_. The bioresin is also pyrolyzed using laser and patterned via photolithography.

A combination of wearable and implantable sensors describes the measurement of temperature, humidity, pressure and strain (all measured from resistance changes), together with salicylic acid and ethylene sensors, and is installed on the leaves of living plants [152]. All of the sensors exhibited stability exceeding 60 days; however, the system was classified as Moderate–High relevance, because it is lacking molecular specificity and relies on indirect stress inference.

**Table 1:** Wearable and implantable plant smart electrochemical sensors—an extensive literature study.

Analyte Category	Nanomaterials/ Nanotechnology— Year of Publication— Wearable/Implantable	Analyte/ Characteristic	Plants	Method	Analytical Parameters (LD, LOD, S)	Field Relevance
**Pesticides and** **fungicides**	PDMS laser-induced graphene (LIG) modified with Nafion-organophosphorus hydrolase on AuNPs [119] using semi-solid electrolyte—2020—wearable	Organophosphorus pesticide: methyl parathion	Crop surfaces: spinach and apple contaminated surfaces	SWV (−0.3 V to 0.3 V) 8 Hz frequency, eq. time 30 s, 0.1 M PBS pH 6 /gelatin semi-solid + 0.1 M PBS pH 6.	LD = 0–500 µM LOD = 0.01 µM S = 2.13 * lg (µM)	**High** Direct on-leaf and fruit-surface measurements without extraction; tolerant to surface irregularities and ambient humidity; suitable for in-field residue screening; no need for buffered solutions.
	Screen-printed electrodes made of PE (polyester) and PLA (lactic acid) [121]—2022—wearable	Carbendazim (1) diquat (2)	Apple and cabbage samples	DPV (0.1 to 0.8 V) 50 mV pulse amplitude, 1 mV step potential, 50 s modulation time. SWV (−1.1 to −0.4 V), 10 Hz frequency, eq. time 5 s, 3 mV step potential, 50 mV pulse amplitude. In 0.1 mM PB solution (pH = 7.0).	LD_1_ = 0–1.4 µM LOD_1_ = 0.094 µPE) LOD_1_ = 0.043 µM (PLA) LD_2_ = 0–1.4 µM LOD_2_ = 0.28 µM (PE) LOD_2_ = 0.057 µM (PLA)	**Moderate–High** Real food matrices, flexible and biodegradable substrates; suitable for on-site crop screening but short-term use (work with drop-casted buffered solutions).
	Cellulose acetate biopolymeric film screen-printed with carbon inks [120]—2023—wearable	Carbendazim (1) paraquat (2)	Lettuce and tomato skins	DPV (0.1 to 0.8 V), 50 mV pulse amplitude, 50 s modulation time, 1 mV step potential; SWV (−1.1 V to −0.4 V), 3 mV step potential, 10 Hz frequency, 50 mV pulse amplitude	LD = 0.1–1.0 μM LOD_1_ = 54.9 nM LOD_2_ = 19.8 nM S_1_ = 0.87 μA/μM S_2_ = 10.08 μA/μM	**Moderate–High** Disposable, sustainable sensors tested directly on crop skins under ambient conditions (work with drop-casted buffered solutions).
	Screen-printed carbon electrodes on kraft paper (SPCE/K-n and SPCE/K-a) in neutral and acidic medium and parchment paper (SPCE/P) [122]—2023—wearable	Carbendazim	Cabbage and skins of apple	DPV (0.4 to 0.75 V), 25 mV modulation amplitude, 0.05 s modulation time, 5 mV step potential, 0.5 s interval time, 0.1 M PB (pH 7)	LD = 0.5–10 μM LOD = 0.17 μM (SPCE/K-n) LOD 0.06 μM (SPCE/K-a) S_1_ = 0.076 μA/μM (SPCE/K-n) S2 = 0.095 μA/μM (SPCE/K-a)	**Moderate** Paper substrates tolerate humidity and handling; suitable for rapid field diagnostics but limited lifetime.
**Markers of abiotic stress** **(saccharides)**	PEDOT:PSS coated with PtNPs and glucose oxidase (GOX) on chitosan matrix [134]—2021—implantable	Glucose and sucrose from xylem sap	Greenhouse grown 8-week-old hybrid aspen trees (xylem sap)	OECT V_GD_ = +0.5 V V_DS_ = −0.4 V (source grounded)	LD = 100 µM–1 mM qualitative	**High** Continuous *in vivo* monitoring of xylem sap over diurnal cycles; strong relevance for real plant physiological tracking.
	Barium ferrite magnetic double-sandwich sensor with agarose, medical tape and screen-printed electrode modified with carbon Prussian blue glucose oxidase + bovine serum albumin Nafion gluteradehyde (CPB/Gox/BSA/Nafion/GA/SPE) [124]—2023—wearable	Glucose	Sweet pepper, gerbera, and romaine lettuce	CA at −0.15 V (vs. Ag/AgCl) for 60 s, 0.1 M, PBS pH 7.4	LD = 20–80 µM LOD = 9.4 µM S = 22.7 nA/(μM⋅cm^2^)	**High** Leaf-attached, non-invasive, multispecies validation; compatible with real-time stress monitoring.
**Ions**	PEDOT:PSS modified Organic Electrochemical Transistors (OECT) [4]—2022—implantable	Ion concentration and saturation in plant sap	Tomato plants	V_ds,o_ = −0.1 V V_gs,o_ = 0.8 V T = 200 s	LD = 0–100 mM	**High** *In vivo* ion and saturation monitoring linked to transpiration and nutrient status; stable operation over hours.
	Photosensitive epoxy bioresin composed of (vitamin B12), graphene oxide (GO), and a photoresist (SU8), followed by laser pyrolysis [34]	Nitrate (NO_3_)	Living maize plants	CV (−0.6 V to 1.2 V) Scan rate = 50 mV/s	LOD = 10–50 µM LD = 0.1–20 mM S = 4.046 μA/ppm	**High** Direct *in planta* sensing, within physiological range; field durability and operates under natural transpiration and nutrient flow.
**Heavy metals**	Bismuth/Nafion-coated carbon working electrode transducer covered with a polyvinyl alcohol (PVA) membrane [126]—2025—wearable	Atmospheric Pb^2+^	Atmosphere of self-adhesive screen-printed sensors attached to plant leaves.	Square wave anodic stripping voltammetry (SWASV)	LOD = ppb range	**High** Real-time airborne heavy metal exposure monitoring directly on plants; self-adhesive.
**Reactive oxygen species (ROS)**	Carbon fiber ultramicroelectrode + hemoglobin + single-wall carbon nanotubes (Hb/SWCNTs/CFUME, ⌀ = 7 μm) [131]—2013—implantable	H_2_O_2_	Aloe leaves, salt stress (0.3 M, NaCl)	CA at −0.1 V, after 12.5 h of treatment	LD = 4.90–405 μM, LOD = 4 µM	**Moderate–High** Early salt-stress detection in living leaves; invasive but physiologically informative.
	Nafion/Pt [130]—2015—implantable	H_2_O_2_ and NO	Oilseed rape leaves (*Brassica napus*) under drought stress	CA at +0.4 V on WE for H_2_O_2_; CA at +0.8 V on WE for NO; 0.01 M pH 7.0 PBS, vs. Ag/AgCl, 20–45 h for H_2_O_2_, 11 and 22 h for NO.	LOD H_2_O_2_ = 1.2 μM, LOD NO = 1.4 μM	**High** Long-term *in vivo* monitoring under real drought stress.
	Gold nanoparticles deposited on indium tin oxide AuNPs/ITO [132]—2020—wearable	H_2_O_2_	Tomato leaves infected with *Botrytis cinerea*	DPV −1.2 V to 0 V, peak increase at cca. −1.0 V, PBS pH 7.4, within 6 and 24 h post inoculation	LD = 0–1 mM LOD = 1 µM	**High** Detection of biotic stress *in situ* during real pathogen infection.
	Polyurethane-based microneedles covered by Au + modification with HRP/Cs-rGO biohydrogel (⌀ = 2 µm) [133]—2025—implantable	H_2_O_2_	Tobacco and soybean leaves, inoculated with fresh *Pto DC3000 culture*	CA at 0.5 V (vs. Ag/AgCl) for 65 s, 0.01 M, PBS pH 7.4 and no electrolyte for plant measurement. 24 h post inoculation	LD = 0.1–4500 μM, LOD = 0.06 μM	**High** Minimal invasiveness, no external electrolyte; strong potential for field deployment.
**Plant hormones**	Pt nanoflowers/electrochemically reduced graphene oxide/electrochemically reduced graphene oxide (PtNF/ERGO/Pt microelectrodes) [145]—2018—implantable	Salicylic acid (SA)	Sunflower seedlings under salt stress	DPV (0.6 V to 1.4 V, 0.02 V increasing potential, 0.02 s pulse width, 0.05 V amplitude, 1 s pulse period, 0.02 s of sampling width)	LD = 100 pM–1 mM LOD = 48.11 pM.	**Moderate–High** *In vivo* stress monitoring but limited to controlled environments
	Cu metal–organic framework (Cu-MOF) and carbon black (CB)–Nafion composite on SPE [10]—2020—wearable with a punching hole and buffer	Salicylic acid (SA)	Leaves of cucumber seedlings	DPV (−1.0 V to 1.5 V, scan rate 0.1 V/s, 0.02 s pulse width, 0.02 V increasing potential, 1 s pulse period, 0.02 s sampling width, 0.05 V amplitude), in 0.05 M Tris–HCl, pH 7.	LOD = 12.5 µM LD = 100–900 µM	**Moderate–High** Leaf-mounted sensor with buffer reservoir; suitable for semi-field monitoring.
	Self-supporting nitrogen-doped graphene microelectrodes [30]—2021—implantable	Salicylic acid (SA)	Plant tissue both *in vivo*/*in vitro*	DPV (0.6 V to 1.2 V) pH 4.5	LD = 1–500 µM S = 0.32–0.14 µA/µM^−1^	**Moderate** *In vivo* capability demonstrated; limited environmental robustness testing.
	Nitrogen-doped carbon nanotubes/core-shell Au@Cu_2_O nanoparticles/carbon fiber electrochemical microsensor [128]—2022—implantable	Indole-3-acetic acid (IAA)	Living cabbage stem	DPV (0.2 V to 1.0 V)	LD = 1–10,000 ng/mL LOD = 10.8–57.8 pg/mL (pH 4–8)	**High** Real-time hormone monitoring in living plants; strong physiological relevance.
	Microneedle-based electrodes magnetic molecularly imprinted polymers (MIPs) [138]—2022—implantable	Salicylic acid (SA)	Tobacco 5 min post-inoculation with *Botrytis cinerea*	CA at 1.1 V, time interval 0.1 s, SA template was incubated on the sensor for 15 min prior to the electrochemical test	LD = 2.74–150 μM LOD = 2.74 μM	**High** Early pathogen-response monitoring *in vivo*
	Core-shell Au@Cu_2_O-graphene-polydopamine (PDA) interdigitated microelectrode array sensor [129]—2021—wearable	Salicylic acid (SA)	Cucumber leaves	DPV (0.0 V to 1.0 V, amplitude 0.05 V, pulse width 0.05 s, pulse period 0.03 s)	LD = 0.01–100 μM LOD = 1.16 nM	**High** Spatially resolved, *in situ* hormone sensing on crop leaves; non-destructive, spatially resolved leaf monitoring.
	Disposable stainless steel (SS)-based electrochemical microsensor modified with Au and Pt nanostructures, reduced graphene oxide (ERGO) and polymerized ST film, PST/Pt-ERGO/Au/a-SS [140]—2019—implantable	Indole-3-acetic acid (IA)	Soybean seedlings	DPV (0.0 V to 1.0 V)	LD = 0.1–100,000 ng mL^−1^ LOD = 43 pg mL^−1^	**High** Demonstrated real-time hormone monitoring in living crop plants with minimal invasiveness; direct hormone tracking linked to growth regulation.
	Au@SnO_2_–vertical graphene microneedle array [143]—2021—implantable microneedle	Abscisic acid (ABA)	Model plants	EIS (frequency 1–50 Hz),	LOD = 0.002 and 0.005 μM LD = 0.012 (or 0.024)–495.2 μM	**High** Stress-responsive hormone measured *in situ* using plant-compatible microneedles.
	Porous laser-induced graphene material engraved on Kapton polyimide and modified with Nafion—LEAFS [141]—2024—wearable	Salicylic acid (SA)	Philodendron brasil leaves and aloe vera	SWV (frequency 4 Hz, Britton–Robinson buffer with a pH of 2.4,	LOD = 6.6–200 µM S = 144.28 μA mM^−1^ LOD = 1.44 µM	**Moderate–High** Flexible, plant-mounted sensor under ambient conditions.
	Sandwich-like laser-induced graphene electrode, Agarose hydrogel [125]—2024—wearable	Salicylic acid (SA)	Avocado plant leaves	CA at 0.8 V applied for 30 s, a sampling time interval of 0.2 s, and an equilibration time of 1 s.	S = 82.3 nA/μmol L^−1^⋅cm^−2^ LOD = 8.2 μmol/L	**Moderate–High** Leaf-mounted, hydration-stabilized wearable platform.
	Copper metal–organic framework-carbon black-Nafion [152]—2023—wearable	Salicylic acid (SA)	Cabbage plants, bell pepper plants for 40 days	DPV (−1.0 V to 1.5 V), 0.01 V step, scan rate of 10 mV/s. Epulse and tpulse were 0.3 V and 0.1 s	LD = 0.1–1000 µM LOD = 0.644 µM	**Moderate–High** Field-tested & continuous, but indirect and non-plant-integrated.
	Copper complex (I)-single-walled carbon nanotube coating [152]—2023—wearable	Ethylene	Cabbage plants, bell pepper plants for 40 days	CV (−0.2 V to 0.5 V), scan rate 50 mV/s, potential step 0.01 V	LD = 0.1–115 ppm LOD = 0.6089 ppm	**Moderate–High** Field-tested & continuous, but indirect and non-plant-integrated.
	Magnetized microneedles coated with superparamagnetic Fe_3_O_4_ intercalated into a scaffold of multi-walled carbon nanotubes (MWCNTs) [139]—2025—implantable	Indole-3-acetic acid (IAA), Salicylic acid (SA)	Tobacco (*N. benthamiana*), *Arabidopsis thaliana* leaves	SWV (0.3 to 1.3 V) with a step size of 5 mV, amplitude of 1 mV, and frequency of 25 Hz. in 0.1 M PBS. Amperometry for	LOD_IAA_ = 1.41 µM LOD_SA_ = 1.15 µM	**High** Minimally invasive, hormone-specific, species-independent.
**Hydration and ionic strength**	Biomimetic organic electrochemical transistor (OECT)—Commercial cotton fibers modified with PEDOT:PSS, ethylene glycol and dodecyl benzene sulfonic acid [147]—2017—implantable	Ionic changes in plant sap; physiology signals	Tomato plants	OECT V_DS_ = 0 to 1 V V_G_ = +1.0 V	No calibration; qualitative; comparison with 0.1 mM NaCl solution; relative response	**Moderate–High** Non-specific but physiologically meaningful signal; scalable device concept; demonstration of plant-integrated electrochemical transistor sensing.
	Commercial textile threads modified with PEDOT:PSS, ethylene glycol and dodecyl benzene sulfonic acid [146]—2019—implantable	Vapor pressure deficit	Tomato plants	OECT Vds = constant V_G_ = +1.0 V	Relative response	**High** Direct linkage between plant electrical signals and atmospheric water demand.
	Organic electrochemical transistor OECT—textile fiber functionalized with PEDOT:PSS) [148]—2019—implantable	Plant’s physiological status; drought stress	Tomato plants	OECT Vds = constant V_G_ = +1.0 V	Qualitative/relative	**High** Early drought stress detection before visual symptoms.
	Two functionalized textile fibers (polypropylene) modified with PEDOT:PSS and dodecyl benzene sulfonic acid ([150]—2023—implantable	Crop water management	Tomato plants	OECT Vds = −0.1 V V_G_ = +0.5 or +0.6 V or 1.0 V	Field-calibrated	**High** Validated under real field conditions for irrigation optimization.
	Interdigited PEDOT:PSS/PDMS hybrid films directlydeposited onto leaves [151]—2025—wearable	Ionic strength and water loss	Tomato plants	Capacitive/impedance hydration sensors	Relative, micromolar LOD by comparison with NaCl solution.	**High** Transparent, conformal, multiplexed sensing with self-powering potential.
**Synthetic plant hormone**	Phosphorene/Ti_3_C_2_-MXene nanohybrid with high ambient stability on laser-induced porous graphene as nanozyme flexible electrode [123]—2021—wearable	α-naphthalene acetic acid (NAA)	Tea, rice, wheat, corn	LSV (0.6 to 1.3 V), 0.1 M PBS (pH 4.0), scan rate 50 mV/s	LD = 0.02–40 μM LOD = 1.6 nM Recoveries 96.66–99.14% RSDs 1.56–4.70%	**High** Multi-crop validation; suitable for on-site agrochemical management.
**Volatile organic compounds**	PtNPs/poly(ATD)/carbon [153]—2022—wearable	Methanol	Maize plants	CA at 0.58 V	LD = 0.5–500 ppm LOD = 0.5 ppm	**High** Non-invasive VOC monitoring directly from plants; strong relevance for early stress diagnostics.
	Au@AgNWs interconnections and multi-walled carbon nanotubes (MWCNTs) embedded in a hydrophobic sol–gel layer made of methyltrimethoxysilane (MTMS) and tetramethyl orthosilicate (TMOS) [7]—2023—wearable	VOC, leaf surface temperature, relative humidity, leaf condensation, leaf strain.	Healthy and pathogen infected tomato plants	Chemoresistive sensors	Relative	**High** Environmental robustness, continuous monitoring, and system integration

Sensitivity (S, µA/(cm^−2^ µM), linear domain (LD, also called LR—linear range or LRD—linear dynamic range, µM—if not stated otherwise) and limit of detection (LoD, µM—if not stated otherwise).

## 7. Commercialization of Electrochemical Sensors for Plants

Even though the scientific community has developed a variety of electrochemical sensors to be used for and on plants, there are still important drawbacks that interfere with their commercialization. Here, we describe some scientific and technological challenges limiting industrialization and commercialization of electrochemical sensors for plants.

**External conditions:** Plants grow and exist over a wide range of pH, humidity, extreme temperatures (especially in this climate change context), UV radiation, etc. Developing electrochemical sensors in these conditions might be difficult when applying them for *in vivo* monitoring [154]. Windy weather needs very good adhesion of the sensors on plants [155]. Leaves can be hairy and covered with wax (in order to protect plants)—this can affect the adhesion of sensors or obtaining the read-out signal, especially with epicuticular wax, which is destined to protect plants from abiotic and biotic stress. It is composed of hydrophobic organic compounds that are also create a non-conductive layer of non-polar hydrocarbons (with very low charge carrier density).

**An integrated electrochemical wearable or implantable device for plants** includes at least the following setup: the sensor itself, the potentiostat and the reading device with an integrated software that can translate the data to the farmer/end user. The integrated device also needs to be energy independent. The power sources of the integrated potentiostat need to be autonomous, because nowadays the smallest potentiostats can only function for around 12 h using their internal battery. Integrating solar batteries into the hardware structure of potentiostats is one of the possible solutions. Another technological challenge is represented by the wireless data transmission (Bluetooth/LoRa/Wi-Fi). Most of the devices use Bluetooth, which can only be used over small distances (usually 10 m, maximum 100 m). This means the implementation of wireless routers in the crop fields. These reading devices should be able to transmit the acquired data into networks similar to a cloud-based network, like the Internet of Things (IoT). This elicits the question of whether farmers (or other stakeholders) are capable of managing this type and large amount of complex data, with proper data analytics. User-friendly interfaces and effective data management are required, in order to get the correct feedback and to implement the correct actions from farmers [155].

**Integration of artificial intelligence (AI) and on-board machine learning (ML):** The integration of the device into a cloud-based network could definitely be improved by the simultaneous integration of artificial intelligence (AI) and on-board machine learning (ML)-based systems with electrochemical sensors to fuse and mine complex electrochemical sensor array data patterns, ultimately enabling the evaluation of overall plant health and suggesting suitable actionable measures to users on their smart devices. This will solve the problem of farmers being capable of processing metadata from the field measurements. This type of approach can transform simple data loggers into intelligent, context-aware plant health advisors. The actionable measures derived from the multiple and complex electrochemical signals can be transferred to a trained, specialized AI-ML system; however, the costs of this approach should be carefully evaluated, as they are currently probably too high to be realistic, given that even the production costs of the hardware assembly components may still be high (including the sensor itself, the potentiostat and the reading device with integrated software). Certainly, integrative AI-ML electrochemical systems will represent an important step toward achieving the integration and autonomous multiplexed operation of high-density redundant or multimodal electrochemical sensor arrays on plants and plant-derived samples, providing real-time remote health insights and recommendations to users in an approachable, user-friendly manner. The redundancy of high-density data can be easily translated into actionable measures using integrative specialized AI, supported by ML to improve with each iteration, creating more accurate model predictions and better recommendations. Ultimately, integrative AI-ML electrochemical devices will bridge the gap between raw sensor signals and meaningful, sustainable plant care decisions. Early disease detection before visual signs, reduced fertilizers and water usage, scalable monitoring of individual plants or entire crops and democratization of expert-level plant diagnostics, are only few of the possible outcomes [156].

**Multiplexing of the electrochemical sensors** can lead to sensor fusion and pattern mining. This involves both intra-sensor fusion with multiple electrochemical channels and inter-sensor fusion by combining electrochemical sensors data with environmental sensor data (such as humidity, temperature, light). As most sensors presented in this review are primarily focused on a single analyte, the possibilities for combining these sensors into multiplexed electrochemical platforms are limited to the designer’s knowledge, creativity and the expected benefits of the final product. Pattern fusing can be performed to adapt sensor multiplexing to real environmental conditions and to replace or reconfigure certain electrochemical sensor arrays for improved symbiosis of the entire device with the specific needs of plants and their environment.

**Scaling-up sensor-based technologies** is also an issue in this stage of development of electrochemical sensors. First of all, the production costs must be reduced using low-cost and scalable mass manufacturing procedures [157]. Even though this issue might be solved, it is also hard to have small reading devices (e.g., the smallest hand-held potentiostats from Palm Sense) that can monitor one plant per crop, as for statistical and practical reasons this is inefficient. Even though statistics may be covered with a significant number of sensors, the cost of this number of sensors has to be reasonable in order to be adapted and implemented by farmers. Depending on the crop type and landscape morphology and topography, as well as other external factors (such as abiotic and biotic stress factors) the number of reading devices may vary a lot, meaning that so does the cost (that it is still too high for end-user acceptance).

**Wearability:** Most of the electrochemical sensors that reached the real application on plants for *in vivo* and *in situ* real-time monitoring were wearable sensors (usually screen-printed carbon electrodes made on flexible adhesive substrates), implantable sensors (usually high mechanical resistance electrodes made of metals that can penetrate the exterior layers of plants such as microneedles) or magnetic electrodes (that could be attached to thin parts of plants, such as leaves). Making them useful for plants directly requires miniaturization and portability of disposable systems. Being disposable requires degradable, eco-friendly and sustainable materials. Even though miniaturization of the sensor itself is possible, the read-out electronic devices are another story.

**Analytical performances of the sensors:** Another gap that hinders commercialization of the electrochemical sensors for agricultural applications is related to the sensitivity and selectivity and the validation of these analytical performances with gold standard measures to attest for their precision and accuracy. Advanced materials (such as graphene and MXene-related materials, phosphorene, etc.) still suffer from reduced reproducibility, have a limited shelf life and encounter difficulties in commercialization, because they oxidize in humid air and they lose conductivity.

**Some existing solutions:** Nevertheless, as far as the authors are aware, commercial electrochemical sensors are quite rare, despite the well-known classical insulin electrochemical-based device for diabetic control in humans. Most of the commercial electrochemical sensors are limited to measuring the characteristics of soil (that is not the purpose of our review). There are some EIS (electrochemical impedance spectroscopy)-based electrochemical sensors (like Cybres, https://cybertronica.biz/) that detect electrophysiological signals in plants. To our knowledge, these sensors measure sap flow measurements using electrochemical electrodes. These kinds of sensors could be complementary to other possible future sensors presented in this review, and could extract even more information regarding important molecules to be used for plant health monitoring in plants.

Some of the commercially available electronic noses are based on electrochemical sensors (like Cyranose 320, https://www.sensigent.com/cyranose-320.html, accessed on 3 December 2025). WOLF 4.1 is a model of e-nose that utilizes commercial electrochemical sensors from AlphaSense Ltd. (Great Notley, Braintree, UK) for the detection of pathogens, such as *Pectobacterium carotovorum* soft-rot infection in potatoes, after intentional inoculation of the pathogen. These sensors are designed for outdoor air quality monitoring and are able to detect markers of biotic stress such as carbon monoxide, ethylene, oxide and nitric oxide. Alphasense LTD electrodes were also used for the TOMATO-NOSE device [53] for the detection of *Botrytis cinerea* in infected tomatoes.

**Some proposed solutions:** The first remark, that authors of the present review have, is that once the sensing scientific community focuses more on “interpreting plant states” than on simply “measuring molecules”, true field-ready systems will emerge. This means a paradigm shift could leads us to tracking physiological trajectories and really solving real environmental issues in smart and precision agriculture.

Even so, some specific materials seem to have reach a High-level of field-relevance. Graphene-based materials are found in most of the High-relevance scored field-deployable sensors, followed by the PEDOT:PSS copolymer (especially in the OECT-based sensors). As graphene-based materials offer a robust conductive backbone, PEDOT:PSS offers transduction and signal amplification. Nafion is the next most frequent material used, offering protection and selectivity. Laser-induced graphene (LIG) is found in at least four of the High-relevance papers and it is a form of 3D graphene that appeared in 2014 and can be created directly on flexible substrates, such as polymers. This way, the materials of the sensor are the same as the substrate of the sensors, and there is less chance of delamination of different material layers. Carbon fibers seem to be the solution for implantable sensors, but not for wearable sensors, as it is difficult to produce them in a large area.

When it comes to the substrate of the sensors, different polymers are used as solutions: cellulose acetate—ecofriendly, paper—disposable, PLA—sustainable, and PDMS—stretchable. However, their role is supporting, not dominant in the sensor design.

Choosing the electrolyte is one of the bottlenecks of wearable and implantable sensor development. The most used electrolytes in the High-relevance sensors are the plant endogenous fluids: xylem sap/apoplastic fluid or leaf moisture. Of course, the first limitation of this solution is the access to the plant endogenous fluids, as this usually works better for implantable sensors. Some of the analytes may not be reachable through the plant fluids. The second-best solution when it comes to electrolyte choice is a hydrogel-integrated-based electrolyte (such as agarose, PVA or biohydrogels—such as chitosan, alginate + Ca^2+^), but they also have a limited lifetime and gradually degrade. The third-best solution includes the solid-state ionomers such as Nafion or PEDOT:PSS, but they have a long-term drift in terms of hydration and an ion selectivity bias. For sure, one of the worse solutions is to drop-cast buffered solutions onto the sensor attached to the leaf, because this buffer will evaporate over the long term, needs constant refilling and changes performance under external conditions (thus, failing under wind, heat or leaf motion). The High-relevance papers use methods that “borrow” or “store” electrolytes, not ones that are “add” electrolytes.

When it comes to chemoresistive approaches, the electrical resistance of MWCNTs varies upon the attachment of VOC molecules on their surface, and it is a feasible choice for this purpose, but chemical specificity should be conferred using other materials.

Multimodal design is one of the high-end approaches in developing wearable sensors. Combining environmental sensors (such as the ones that measure external conditions) with plant physiology sensors (with specific biochemistry) offers a complementarity that none of the described sensors really captured. Most designs envision either one (measuring external conditions) or the other (biochemical specificity), even when it comes to multimodal designs. But proof-of-concept exists, as already discussed above [7]. This last paper also provides machine learning integration, already described as a solution of wearable and implantable electrochemical sensors for smart and precise agriculture.

Combining electrochemical multimodal sensors with other techniques such as Raman of infrared sensing could exponentially improve the analytical power of the sensor.

Last, but not least, the Design of Experiments (DOE) could be useful, as one could really study if specific variables are statistically significant for the developed sensor, and if components or concentrations have positive, negative or negligible effects on the sensor design. This would lead to more solid designs, one of the most important characteristics for field measurements.

## 8. Conclusions: Are Electrochemical Sensors Ready for Plant Health Monitoring?

Despite recent impressive analytical advances in wearable and implantable electrochemical sensors for plant health monitoring, most plant electrochemical sensors still face systemic limitations that restrict real agricultural deployment. Most of the presented papers should be interpreted with caution, as most of them derive from controlled buffer experiments, which are not really suitable for field-deployable sensors. Long-term stability, calibration drift, biofouling and environmental robustness remain insufficiently addressed in most studies. The described technologies should not be seen as fully matured solutions, but more likely as components for next-generation plant health monitoring platforms. The potential general impact of the presented work is bridging the laboratory-based analytical performances to deployable agricultural systems.

Specifically, the examples presented in the work related to *bioristors* [4] could have a real impact on water–nutrient coupling analysis, stress onset detection before visible symptoms, and decision-support systems for irrigation and fertilization, as this biomimetic and biocompatible OECT sensor enables ionic concentration and saturation monitoring in plant sap. Nitrate sensors have the potential to enable real-time fertilization optimization and nutrient stress detection [34]. Nevertheless, this still needs to be demonstrated and implemented. Fundamental limitations of the DNA amplification-based plant-pathogen sensors, such as their reliance on enzyme amplification, which is usually performed in the laboratory under controllable parameters in terms of temperature, humidity, UV, etc. and using multistep workflows, likely makes these sensors more important for confirmative diagnostic work (performed in the lab or on portable platforms), rather than for use as field-deployable tools for real-time plant health monitoring in smart and precise agriculture. Other factors that are opposed to the final goal include high energy dependency, non-continuous systems, reagent-intensive operation and high human-dependent maintenance.

While electrochemical sensors for antioxidants and phenolic compounds demonstrate excellent analytical sensitivity and selectivity, the majority of published platforms rely on extract-based measurements and laboratory-controlled conditions. Consequently, their direct applicability to real-time, in-field plant health monitoring remains limited. These sensors are best positioned as complementary tools for stress severity assessment, crop quality evaluation, and phenotyping rather than primary early warning systems in smart and precision agriculture.

While many of the presented studies regarding pesticides/fungicides have applications in environmental control and food safety, most of them are based on extracted samples of exogeneous agrochemicals or secondary metabolites, limiting their use for real-time plant monitoring. Only a few of them have High and Moderate–High relevance for field measurements, because they were developed into wearable devices that were applied under field-realistic conditions, but only one paper describes a semi-solid-state electrolyte integrated into the sensor design [119].

Early stress detection becomes one of the most important must-haves in smart and precision agriculture, as symptoms of stress usually become visible when it is too late to save plants or crops. Most methods detect the response of plants to stress, not the stress directly induced in plants. This eventually means that the best way to detect early signs of stress in plants is to directly detect the molecules that induce the stress, namely the reactive oxygen and nitrogen species (ROS/RNS), rather than the biomarkers that appear as a consequence of these stress-inducing molecules (such as malondialdehyde—MDA, quinoline, phytohormones, antioxidants, etc.). ROS are one of the most important molecule categories to be detected when it comes to plant health monitoring, as they can offer a temporal scale of stress detection. Very few papers describe fast-acquiring signals for early stress detection using electrochemical sensors. This implies the need of further knowledge of plant physiology among researchers developing sensors for plants. From a sensing point of view, directly detecting plant stress is more relevant than detecting the plant’s response to stress itself. Nevertheless, the electrochemical sensors for ROS are quite well represented, but the diversity of targeted molecules is limited to hydrogen peroxide. More disruptive thinking should address additional ROS species, or at least better distinguish which ROS are most relevant to detect in plants and at which moment after external stress occurs. No ROS sensor has demonstrated weeks-long, drift-corrected, species-independent, environmentally robust in-field operation with validated physiological interpretation.

Even minimally invasive sensors could trigger wound responses, thus inducing ROS burst, salicylic acid (SA) or jasmonic acid (JA) signaling, altering local metabolite concentration and composition and disrupting pressure gradients in the xylem, etc. In this way, sensor measurements may reflect *sensor-induced stress*, rather than native physiology. This type of tissue damage is more likely observed in implantable sensors. On the other hand, wearable sensors suffer from other limitations, such as surface-only information and poor long-term adhesion, especially under rain or wind conditions. However, this approach remains more relevant for early stress monitoring.

Phytohormones are the second-best represented analyte category. Even though this category includes a higher number of High-relevance papers, phytohormones exhibit more challenging electrochemistry and lower stability in terms of developed sensing approaches.

Taking into account all of the presented data, we can conclude that electrochemical (bio)sensors based on nanotechnologies for the detection of important biomolecules in plants and plant-related samples could represent the future of smart and precision agriculture. However, several challenges remain before they become attractive for commercialization and public acceptance, as well as more convincing, including the need for more solid field testing and validation of the proposed sensors (considering proposed solutions in the present review).

## Data Availability

No new data were created or analyzed in this study. Data sharing is not applicable to this article.

## Supplementary Materials

The following supporting information can be downloaded at: https://www.mdpi.com/article/10.3390/bios16020107/s1, Figure S1. (A) Article selection process flowchart. Publications over time of the selected articles: (B) Web Of Science (WOS) and (C) Scopus; Table S1. Definition of PICO strategy applied for the present work; Table S2. Electrochemical detection of different categories of biomolecules detected in plant-related samples, with the corresponding materials and performance electrochemical characteristics of the described methods. An extensive literature study; all the references included in this extensive literature study are cited in the main text; Table S3. Future perspectives.

## References

[1] Food and Agriculture Organization of the United Nations About FAO’s Work on Plant Production and Protection. https://www.fao.org/plant-production-protection/about/en.

[2] Kalia A., Abd-Elsalam K.A., Kuca K. (2020). Zinc-Based Nanomaterials for Diagnosis and Management of Plant Diseases: Ecological Safety and Future Prospects. J. Fungi.

[3] Kim M.-Y., Lee K.H. (2022). Electrochemical Sensors for Sustainable Precision Agriculture—A Review. Front. Chem..

[4] Gentile F., Vurro F., Janni M., Manfredi R., Cellini F., Petrozza A., Zappettini A., Coppede N. (2022). A Biomimetic, Biocompatible OECT Sensor for the Real-Time Measurement of Concentration and Saturation of Ions in Plant Sap. Adv. Electron. Mater..

[5] Seddaoui N., Arduini F. (2025). Recent advances in wearable and implantable electrochemical (bio)sensors for plant health monitoring. TrAC Trends Anal. Chem..

[6] Fang Y., Umasankar Y., Ramasamy R.P. (2016). A novel bi-enzyme electrochemical biosensor for selective and sensitive determination of methyl salicylate. Biosens. Bioelectron..

[7] Lee G., Hossain O., Jamalzadegan S., Liu Y., Wang H., Saville A.C., Shymanovich T., Paul R., Rotenberg D., Whitfield A.E. (2023). Abaxial leaf surface-mounted multimodal wearable sensor for continuous plant physiology monitoring. Sci. Adv..

[8] Giraldo J.P., Wu H., Newkirk G.M., Kruss S. (2019). Nanobiotechnology approaches for engineering smart plant sensors. Nat. Nanotechnol..

[9] Bard A.J., Faulkner L.R., White H.S. (2022). Electrochemical Methods: Fundamentals and Applications.

[10] Yang L., Chen D., Wang X., Luo B., Wang C., Gao G., Li H., Li A., Chen L. (2020). Ratiometric electrochemical sensor for accurate detection of salicylic acid in leaves of living plants. RSC Adv..

[11] Madadelahi M., Romero-Soto F.O., Kumar R., Tlaxcala U.B., Madou M.J. (2025). Electrochemical sensors: Types, applications, and the novel impacts of vibration and fluid flow for microfluidic integration. Biosens. Bioelectron..

[12] Saxena S., Shrivastava R., Satsangee S.P. (2015). Voltammetric determination of wedelolactone, an anti-HIV herbal drug, at boron-doped diamond electrode. J. Chem. Sci..

[13] Singh S., Kumar N., Kumar M., Agarwal A., Mizaikoff B. (2017). Electrochemical sensing and remediation of 4-nitrophenol using bio-synthesized copper oxide nanoparticles. Chem. Eng. J..

[14] Zhang Z., Song M., Hao J., Wu K., Li C., Hu C. (2018). Visible light laser-induced graphene from phenolic resin: A new approach for directly writing graphene-based electrochemical devices on various substrates. Carbon.

[15] Zhang J., Lu M., Zhou H., Du X., Du X. (2022). Assessment of Salt Stress to Arabidopsis Based on the Detection of Hydrogen Peroxide Released by Leaves Using an Electrochemical Sensor. Int. J. Mol. Sci..

[16] Salam U., Ullah S., Tang Z.H., Elateeq A.A., Khan Y., Khan J., Khan A., Ali S. (2023). Plant Metabolomics: An Overview of the Role of Primary and Secondary Metabolites against Different Environmental Stress Factors. Life.

[17] Fang Y., Bullock H., Lee S.A., Sekar N., Eiteman M.A., Whitman W.B., Ramasamy R.P. (2016). Detection of methyl salicylate using bi-enzyme electrochemical sensor consisting salicylate hydroxylase and tyrosinase. Biosens. Bioelectron..

[18] Liu K., Wang X.D., Luo B., Wang C., Hou P.C., Dong H.T., Li A.X., Zhao C.J. (2022). Enzyme-Free Electrochemical Sensors for in situ Quantification of Reducing Sugars Based on Carboxylated Graphene-Carboxylated Multiwalled Carbon Nanotubes-Gold Nanoparticle-Modified Electrode. Front. Plant Sci..

[19] Wilson A.D. (2018). Applications of Electronic-Nose Technologies for Noninvasive Early Detection of Plant, Animal and Human Diseases. Chemosensors.

[20] Schlagmann M., Balendonck J., Otto T., de Assis M.B.S., Mertig M., Hess S. (2023). Development of sensor nodes and sensors for smart farming. J. Electrochem. Sci. Eng..

[21] Umasankar Y., Rains G.C., Ramasamy R.P. (2012). Electroanalytical studies on green leaf volatiles for potential sensor development. Analyst.

[22] Velasco-Medina C., Espinoza-Montero P.J., Montero-Jimenez M., Alvarado J., Jadán M., Carrera P., Fernandez L. (2019). Development and Evaluation of Copper Electrodes, Modified with Bimetallic Nanoparticles, to be Used as Sensors of Cysteine-Rich Peptides Synthesized by Tobacco Cells Exposed to Cytotoxic Levels of Cadmium. Molecules.

[23] Dago A., Navarro J., Arino C., Diaz-Cruz J.M., Esteban M. (2015). Carbon nanotubes and graphene modified screen-printed carbon electrodes as sensitive sensors for the determination of phytochelatins in plants using liquid chromatography with amperometric detection. J. Chromatogr. A.

[24] Akanbi F.S., Yusof N.A., Abdullah J., Sulaiman Y., Hushiarian R. (2017). Detection of Quinoline in *G. boninense*-Infected Plants Using Functionalized Multi-Walled Carbon Nanotubes: A Field Study. Sensors.

[25] Li Z.L., Zhou J.P., Dong T., Xu Y., Shang Y.K. (2021). Application of electrochemical methods for the detection of abiotic stress biomarkers in plants. Biosens. Bioelectron..

[26] Sun L.-J., Xie Y., Yan Y.-F., Yang H., Gu H.-Y., Bao N. (2017). Paper-based analytical devices for direct electrochemical detection of free IAA and SA in plant samples with the weight of several milligrams. Sens. Actuators B Chem..

[27] Janssen S., Schmitt K., Blanke M., Bauersfeld M.L., Wöllenstein J., Lang W. (2014). Ethylene detection in fruit supply chains. Philos. Trans. R. Soc. A Math. Phys. Eng. Sci..

[28] Liu J.T., Hu L.S., Liu Y.L., Chen R.S., Cheng Z., Chen S.J., Amatore C., Huang W.H., Huo K.F. (2014). Real-time monitoring of auxin vesicular exocytotic efflux from single plant protoplasts by amperometry at microelectrodes decorated with nanowires. Angew. Chem. Int. Ed..

[29] You Y., Luo B., Wang C., Dong H.T., Wang X.D., Hou P.C., Sun L.J., Li A.X. (2023). An ultrasensitive probe-free electrochemical immunosensor for gibberellins employing polydopamine-antibody nanoparticles modified electrode. Bioelectrochemistry.

[30] Zhang F., Li M.J., Li H.J., Wang G.L., Long Y.B., Li P.H., Li C.P., Yang B.H. (2021). Fabrication of self-supporting nitrogen-doped graphene microelectrodes for in situ analysis of salicylic acid in plants. Carbon.

[31] Xing G.Q., Wang C., Liu K., Luo B., Hou P.C., Wang X.D., Dong H.T., Wang J.S., Li A.X. (2022). A probe-free electrochemical immunosensor for methyl jasmonate based on a Cu-MOF-carboxylated graphene oxide platform. RSC Adv..

[32] Sha R., Kadu A., Matsumoto K., Uno S., Badhulika S. (2020). Ultra-low cost, smart sensor based on pyrite FeS2 on cellulose paper for the determination of vital plant hormone methyl jasmonate. Eng. Res. Express.

[33] Fang Y., Zhou Y., Ramasamy R.P. (2018). Communication-Direct Detection of Methyl Salicylate Using Tri-Enzyme Based Electrochemical Sensor. J. Electrochem. Soc..

[34] Ibrahim H., Yin S., Moru S., Zhu Y., Castellano M.J., Dong L. (2022). In Planta Nitrate Sensor Using a Photosensitive Epoxy Bioresin. ACS Appl. Mater. Interfaces.

[35] Xie Q.J., He W.Y., Yu S., Chen X.Y., Zhang X., Shen Y.H. (2014). Sensitive sensors for amperometric detection of nitrite based on carbon-supported PdNi and PdCo bimetallic nanoparticles. Anal. Methods.

[36] Sekhon B.S. (2014). Nanotechnology in agri-food production: An overview. Nanotechnol. Sci. Appl..

[37] Su H.C., Zhang M., Bosze W., Lim J.H., Myung N.V. (2013). Metal nanoparticles and DNA co-functionalized single-walled carbon nanotube gas sensors. Nanotechnology.

[38] Nagamine K., Kudo N., Sasaki H., Asano A., Iwasa S. (2023). Continuous Extraction and Electrochemical Monitoring of Potassium Ions in a Plant Leaf Using a Wearable Ion Sensor. Sens. Mater..

[39] Guo L.H., Liu Y.C., Liu L., Yin P.H., Liu C., Li J.M. (2023). Study of the mechanism of embolism removal in xylem vessels by using microfluidic devices. Lab Chip.

[40] Roy E., Patra S., Madhuri R., Sharma P.K. (2014). Simultaneous determination of heavy metals in biological samples by a multiple-template imprinting technique: An electrochemical study. RSC Adv..

[41] Mitra S., Purkait T., Pramanik K., Maiti T.K., Dey R.S. (2019). Three-dimensional graphene for electrochemical detection of Cadmium in *Klebsiella michiganensis* to study the influence of Cadmium uptake in rice plant. Mater. Sci. Eng. C Mater. Biol. Appl..

[42] Liendo F., de la Vega A.P., Aguirre M.J., Godoy F., Martí A.A., Flores E., Pizarro J., Segura R. (2022). A simple graphene modified electrode for the determination of antimony (III) in edible plants and beverage. Food Chem..

[43] Lau H.Y., Wu H., Wee E.J.H., Trau M., Wang Y., Botella J.R. (2017). Specific and Sensitive Isothermal Electrochemical Biosensor for Plant Pathogen DNA Detection with Colloidal Gold Nanoparticles as Probes. Sci. Rep..

[44] Fang Y., Umasankar Y., Ramasamy R.P. (2014). Electrochemical detection of p-ethylguaiacol, a fungi infected fruit volatile using metal oxide nanoparticles. Analyst.

[45] Nalini S., Nandini S., Reddy M.B.M., Suresh G.S., Melo J.S., Neelagund S.E., NaveenKumar H.N., Shanmugam S. (2016). A novel bioassay based gold nanoribbon biosensor to aid the preclinical evaluation of anticancer properties. RSC Adv..

[46] Wang Y., Li B., Liu J., Zhou H. (2019). T4 DNA polymerase-assisted upgrade of a nicking/polymerization amplification strategy for ultrasensitive electrochemical detection of Watermelon mosaic virus. Anal. Bioanal. Chem..

[47] Tahir M.A., Bajwa S.Z., Mansoor S., Briddon R.W., Khan W.S., Scheffler B.E., Amin I. (2018). Evaluation of carbon nanotube based copper nanoparticle composite for the efficient detection of agroviruses. J. Hazard. Mater..

[48] Hu Y.W., Yang T., Li Q.H., Guan Q., Jiao K. (2013). Conjugated self-doped polyaniline-DNA hybrid as trigger for highly sensitive reagentless and electrochemical self-signal amplifying DNA hybridization sensing. Analyst.

[49] Khater M., de la Escosura-Muñiz A., Quesada-González D., Merkoçi A. (2019). Electrochemical detection of plant virus using gold nanoparticle-modified electrodes. Anal. Chim. Acta.

[50] Isha A., Akanbi F.S., Yusof N.A., Osman R., Mui-Yun W., Abdullah S.N.A. (2019). An NMR Metabolomics Approach and Detection of *Ganoderma boninense*-Infected Oil Palm Leaves Using MWCNT-Based Electrochemical Sensor. J. Nanomater..

[51] Davis D., Guo X., Musavi L., Lin C.-S., Chen S.-H., Wu V.C.H. (2013). Gold Nanoparticle-Modified Carbon Electrode Biosensor for the Detection of Listeria monocytogenes. Ind. Biotechnol..

[52] Vatankhah A., Reezi S., Izadi Z., Ghasemi-Varnamkhasti M., Motamedi A. (2022). Development of an ultrasensitive electrochemical biosensor for detection of *Agrobacterium tumefaciens* in *Rosa hybrida* L.. Measurement.

[53] Meléndez F., Sánchez R., Fernández J.A., Belacortu Y., Bermúdez F., Arroyo P., Martín-Vertedor D., Lozano J. (2023). Design of a Multisensory Device for Tomato Volatile Compound Detection Based on a Mixed Metal Oxide-Electrochemical Sensor Array and Optical Reader. Micromachines.

[54] Patel R., Vinchurkar M., Mohin Shaikh A., Patkar R., Adami A., Giacomozzi F., Ramesh R., Pramanick B., Lorenzelli L., Shojaei Baghini M. (2023). Part I: Non-faradaic electrochemical impedance-based DNA biosensor for detecting phytopathogen—*Ralstonia solanacearum*. Bioelectrochemistry.

[55] Patel R., Vinchurkar M., Shaikh A.M., Patkar R., Adami A., Giacomozzi F., Ramesh R., Pramanick B., Lorenzelli L., Baghini M.S. (2023). Part II: Impedance-based DNA biosensor for detection of isolated strains of phytopathogen *Ralstonia solanacearum*. Bioelectrochemistry.

[56] Singh N., Khan R.R., Xu W.H., Whitham S.A., Dong L. (2023). Plant Virus Sensor for the Rapid Detection of *Bean pod mottle* Virus Using Virus-Specific Nanocavities. ACS Sensors.

[57] Wendlandt T., Koch C., Britz B., Liedek A., Schmidt N., Werner S., Gleba Y., Vahidpour F., Welden M., Poghossian A. (2023). Facile Purification and Use of Tobamoviral Nanocarriers for Antibody-Mediated Display of a Two-Enzyme System. Viruses.

[58] Rashid J.I.A., Kannan V., Ahmad M.H., Mon A.A., Taufik S., Miskon A., Ong K.K., Yusof N.A. (2021). An electrochemical sensor based on gold nanoparticles-functionalized reduced graphene oxide screen printed electrode for the detection of pyocyanin biomarker in *Pseudomonas aeruginosa* infection. Mater. Sci. Eng. C.

[59] Chen J., Wu C., Zhao Z., Xue R. (2025). An Electrochemical Aptamer Sensor with ZIF-8 Loaded CuNPs Composites for Aflatoxin B1 Determination. Chemosensors.

[60] Ansari A.A., Kaushik A., Solanki P.R., Malhotra B.D. (2010). Nanostructured zinc oxide platform for mycotoxin detection. Bioelectrochemistry.

[61] Zhao S., Zang G., Zhang Y., Liu H., Wang N., Cai S., Durkan C., Xie G., Wang G. (2021). Recent advances of electrochemical sensors for detecting and monitoring ROS/RNS. Biosens. Bioelectron..

[62] Akter S., Khan M.S., Smith E.N., Flashman E. (2021). Measuring ROS and redox markers in plant cells. RSC Chem. Biol..

[63] Wen W., Chen W., Ren Q.-Q., Hu X.-Y., Xiong H.-Y., Zhang X.-H., Wang S.-F., Zhao Y.-D. (2012). A highly sensitive nitric oxide biosensor based on hemoglobin–chitosan/graphene–hexadecyltrimethylammonium bromide nanomatrix. Sens. Actuators B Chem..

[64] Yao Y., Liu X., Shao Y., Ying Y., Ping J. (2020). Noble metal alloy nanoparticles coated flexible MoS_2_ paper for the determination of reactive oxygen species. Biosens. Bioelectron..

[65] Lima A.S., Prieto K.R., Santos C.S., Paula Valerio H., Garcia-Ochoa E.Y., Huerta-Robles A., Beltran-Garcia M.J., Di Mascio P., Bertotti M. (2018). In-vivo electrochemical monitoring of H_2_O_2_ production induced by root-inoculated endophytic bacteria in Agave tequilana leaves. Biosens. Bioelectron..

[66] Hosu I.S., Constantinescu-Aruxandei D., Oancea F., Doni M. (2021). The Scavenging Effect of Myoglobin from Meat Extracts toward Peroxynitrite Studied with a Flow Injection System Based on Electrochemical Reduction over a Screen-Printed Carbon Electrode Modified with Cobalt Phthalocyanine: Quantification and Kinetics. Biosensors.

[67] Ribeiro C.M., Miguel E.M., Silva J.d.S., da Silva C.B., Goulart M.O.F., Kubota L.T., Gonzaga F.B., Santos W.J.R., Lima P.R. (2016). Application of a nanostructured platform and imprinted sol-gel film for determination of chlorogenic acid in food samples. Talanta.

[68] Fu L., Liu Z., Huang Y., Lai G., Zhang H., Su W., Yu J., Wang A., Lin C.-T., Yu A. (2018). Square wave voltammetric quantitative determination of flavonoid luteolin in peanut hulls and *Perilla* based on Au NPs loaded boron nitride nanosheets. J. Electroanal. Chem..

[69] Gopal P., Reddy T.M., Palakollu V.N. (2017). Development, Characterization and Application of a Carbon-Based Nanomaterial Composite as an Electrochemical Sensor for Monitoring Natural Antioxidant (Gallic Acid) in Beverages. Chemistryselect.

[70] El Jaouhari A., Yan L.Y., Zhu J.H., Zhao D.B., Khan M.Z.H., Liu X.H. (2020). Enhanced molecular imprinted electrochemical sensor based on zeolitic imidazolate framework/reduced graphene oxide for highly recognition of rutin. Anal. Chim. Acta.

[71] Gomes dos Santos Neto A., de Matos Morawski F., Caroline Ferreira Santos A., Quintino da Rocha C., Batista de Lima R., Oliveira Fonseca Goulart M., Costa dos Santos C., Colmati F., Euzébio Goulart Santana A., Aurélio Suller Garcia M. (2023). Host-guest Assembly Based on γ-Cyclodextrin-functionalized Multiwalled Carbon Nanotubes for Rutin Electrochemical Sensing. Electroanalysis.

[72] Pliuta K., Chebotarev A., Koicheva A., Bevziuk K., Snigur D. (2018). Development of a novel voltammetric sensor for the determination of quercetin on an electrochemically pretreated carbon-paste electrode. Anal. Methods.

[73] Sun B.L., Yang Y.M., Sun Y.L., Wu D., Kan L., Gao C.Y., Shi H.X., Sang C.Y., Zhao T.K., Yang L. (2023). Evaluating the antioxidant activity of secondary metabolites of endophytic fungi from *Hypericum perforatum* L. by an electrochemical biosensor based on AuNPs/AC@CS composite. Bioelectrochemistry.

[74] Li J.D., Wang C.X., Chen X.L., Huang M.H., Fu Q., Li R.J., Wang Y.L., Li C.Y., Zhao P.C., Xie Y.X. (2022). A non-enzymatic photoelectrochemical sensor based on g-C_3_N_4_@CNT heterojunction for sensitive detection of antioxidant gallic acid in food. Food Chem..

[75] Sarafraz S., Rafiee-Pour H.-A., Khayatkashani M., Ebrahimi A. (2019). Electrochemical determination of gallic acid in *Camellia sinensis*, *Viola odorata*, *Commiphora mukul*, and *Vitex agnus*-castus by MWCNTs-COOH modified CPE. J. Nanostruct..

[76] Chokkareddy R., Redhi G.G. (2020). Ionic Liquid and f-MWCNTs Fabricated Glassy Carbon Electrode for Determination of Amygdalin in Apple Seeds. Electroanalysis.

[77] Xia J., Liu F., Yan S., Suo H., Qian J., Zou B. (2023). Simultaneous determination of tert-butylhydroquinone, butylated hydroxyanisole and phenol in plant oil by metalloporphyrin-based covalent organic framework electrochemical sensor. J. Food Compos. Anal..

[78] Wang Y.L., Ni M.J., Chen J., Wang C.X., Yang Y.Q., Xie Y.X., Zhao P.C., Fei J.J. (2023). An ultra-sensitive luteolin sensor based on Co-doped nitrogen-containing carbon framework/MoS_2_-MWCNTs composite for natural sample detection. Electrochim. Acta.

[79] Gomez F.J.V., Espino M., de los Angeles Fernandez M., Raba J., Silva M.F. (2016). Enhanced electrochemical detection of quercetin by Natural Deep Eutectic Solvents. Anal. Chim. Acta.

[80] Pei F.B., Wu Y., Feng S.S., Wang H.L., He G.Y., Hao Q.L., Lei W. (2022). Palladium Nanoparticle-Modified Carbon Spheres @ Molybdenum Disulfide Core-Shell Composite for Electrochemically Detecting Quercetin. Chemosensors.

[81] Pwavodi P.C., Ozyurt V.H., Asir S., Ozsoz M. (2021). Electrochemical Sensor for Determination of Various Phenolic Compounds in Wine Samples Using Fe_3_O_4_ Nanoparticles Modified Carbon Paste Electrode. Micromachines.

[82] Liang Y., Zhang L.Y., Wang H.M., Cai X.R., Zhang L., Xu Y.X., Yao C.X., Si W.S., Huang Z.P., Shi G.Y. (2023). Fabrication of a novel electrochemical sensor based on tin disulfide/multi-walled carbon nanotunbes-modified electrode for rutin determination in natural vegetation. Arab. J. Chem..

[83] Temerk Y., Ibrahim H. (2016). Fabrication of a novel electrochemical sensor based on Zn-In_2_O_3_ nanorods coated glassy carbon microspheres paste electrode for square wave voltammetric determination of neuroprotective hibifolin in biological fluids and in the flowers of hibiscus vitifolius. J. Electroanal. Chem..

[84] Zhang B.W., El Jaouhari A., Wu X.R., Liu W., Zhu J.H., Liu X.H. (2020). Synthesis and characterization of PEDOT-MC decorated AgNPs for voltammetric detection of rutin in real samples. J. Electroanal. Chem..

[85] Xing Y.F., Zhang C., Chen X.Y., Zhao H.M., Guo Z.J. (2021). Highly sensitive detection of salvianic acid a drug by a novel electrochemical sensor based on HKUST-1 loaded on three-dimensional graphene-MWCNT composite. J. Pharm. Biomed. Anal..

[86] Lima F.M.D., Freires A.D., Pereira N.D., Silva G.G., da Rocha C.Q., Damos F.S., Luz R.D.S. (2018). Photoelectrochemical sensing of tannic acid based on the use of TiO_2_ sensitized with 5-methylphenazinium methosulfate and carboxy-functionalized CdTe quantum dots. Microchim. Acta.

[87] Monari A., Cantalù S., Zanfrognini B., Brighenti V., Verri P., Zanardi C., Pellati F., Pigani L. (2023). An electrochemical approach for the prediction of Δ9-tetrahydrocannabinolic acid and total cannabinoid content in *Cannabis sativa* L.. Analyst.

[88] Rupasinghe H.P.V., Davis A., Kumar S.K., Murray B., Zheljazkov V.D. (2020). Industrial Hemp (*Cannabis sativa* subsp. *sativa*) as an Emerging Source for Value-Added Functional Food Ingredients and Nutraceuticals. Molecules.

[89] Peng Y.Q., Zhang W.J., Chang J., Huang Y.P., Chen L., Deng H., Huang Z., Wen Y.P. (2017). A Simple and Sensitive Method for the Voltammetric Analysis of Theobromine in Food Samples Using Nanobiocomposite Sensor. Food Anal. Methods.

[90] Gao J., Li H., Li M., Wang G., Long Y., Li P., Li C., Yang B. (2021). Polydopamine/graphene/MnO_2_ composite-based electrochemical sensor for in situ determination of free tryptophan in plants. Anal. Chim. Acta.

[91] Wang Y., Mamat X., Li Y.T., Hu X., Wang P., Dong Y.M., Hu G.Z. (2019). Glassy Carbon Electrode Modified via Molybdenum Disulfide Decorated Multiwalled Carbon Nanotubes for Sensitive Voltammetric Detection of Aristolochic Acids. Electroanalysis.

[92] Zhou M., Tang T., Deng X., Li Q., Zuo Z., Hu G. (2023). MoS_2_ nanosheets grown on bowl-shaped hollow carbon spheres as an efficient electrochemical sensor for ultrasensitive determination of nephrotoxic aristolochic acids in Chinese traditional herbs. Anal. Methods.

[93] El Jaouhari A., Wang Y., Zhang B.W., Liu X.H., Zhu J.H. (2020). Effect of surface properties on the electrochemical response of cynarin by electro-synthesized functionalized-polybithiophene/MWCNT/GNP. Mater. Sci. Eng. C Mater. Biol. Appl..

[94] Ajermoun N., Hrioua A., Chhaibi B., Laghrib F., Farahi A., Lahrich S., Bakasse M., Saqrane S., El Mhammedi M.A. (2023). Electrochemical monitoring of thiamethoxam in *Zea mays* and *Phaseolus vulgaris* L. plants using chitosan stabilized silver nanoparticles electrode. Food Chem. Adv..

[95] Pandey A., Sharma S., Jain R. (2019). Voltammetric sensor for the monitoring of hazardous herbicide triclopyr (TCP). J. Hazard. Mater..

[96] Gerbreders V., Krasovska M., Mihailova I., Ogurcovs A., Sledevskis E., Gerbreders A., Tamanis E., Kokina I., Plaksenkova I. (2021). Nanostructure-based electrochemical sensor: Glyphosate detection and the analysis of genetic changes in rye DNA. Surf. Interfaces.

[97] Karuppaiah B., Jeyaraman A., Chen S.-M., Chavan P.R., Karthik R., Hasan M., Shim J.-J. (2023). Effect of bismuth doping on zircon-type gadolinium vanadate: Effective electrocatalyst for determination of hazardous herbicide mesotrione. Chemosphere.

[98] Zhao Y., Zheng X., Wang Q., Zhe T., Bai Y., Bu T., Zhang M., Wang L. (2020). Electrochemical behavior of reduced graphene oxide/cyclodextrins sensors for ultrasensitive detection of imidacloprid in brown rice. Food Chem..

[99] Elbaz G.A., Zaazaa H.E., Monir H.H., Abd El Halim L.M., Atty S.A. (2022). Nano eco-friendly voltammetric determination of pesticide, imidacloprid and its residues in thyme and guava leaves. Sustain. Chem. Pharm..

[100] zahirifar F., Rahimnejad M., Abdulkareem R.A., Najafpour G. (2019). Determination of Diazinon in fruit samples using electrochemical sensor based on carbon nanotubes modified carbon paste electrode. Biocatal. Agric. Biotechnol..

[101] Zhang C.X., Huang H., Zhou J., Hu C.C., Li S., Wei D., Tan Y.M., Deng Y. (2022). A Sensitive Electrochemical Aptasensor Based on Black Phosphorus Nanosheet for Carbaryl Detection. Sci. Adv. Mater..

[102] Ayhan E.A., Inam R. (2020). Square wave stripping voltammetric determination of cyprodinil fungicide in food samples by nanostructured multi walled carbon nanotube paste electrode. J. Food Meas. Charact..

[103] Caratelli V., Fegatelli G., Moscone D., Arduini F. (2022). A paper-based electrochemical device for the detection of pesticides in aerosol phase inspired by nature: A flower-like origami biosensor for precision agriculture. Biosens. Bioelectron..

[104] Wang M., Yang Z.Q., Guo Y.L., Wang X.X., Yin H.S., Ai S.Y. (2015). Visible-light induced photoelectrochemical biosensor for the detection of microRNA based on Bi_2_S_3_ nanorods and streptavidin on an ITO electrode. Microchim. Acta.

[105] Zhou Y., Wang M., Xu Z., Ni C., Yin H., Ai S. (2014). Investigation of the effect of phytohormone on the expression of microRNA-159a in *Arabidopsis thaliana* seedlings based on mimic enzyme catalysis systematic electrochemical biosensor. Biosens. Bioelectron..

[106] Mohiuddin M., Arbain D., Islam A., Ahmad M.S., Ahmad M.N. (2016). Alpha-Glucosidase Enzyme Biosensor for the Electrochemical Measurement of Antidiabetic Potential of Medicinal Plants. Nanoscale Res. Lett..

[107] Mohiuddin M., Arbain D., Shafiqul Islam A.K.M., Rahman M., Ahmad M.S., Ahmad M.N. (2015). Electrochemical measurement of the antidiabetic potential of medicinal plants using multi-walled carbon nanotubes paste electrode. Russ. J. Electrochem..

[108] Yang J., Wang X., Shi H. (2012). An electrochemical DNA biosensor for highly sensitive detection of phosphinothricin acetyltransferase gene sequence based on polyaniline-(mesoporous nanozirconia)/poly-tyrosine film. Sens. Actuators B Chem..

[109] Lin Y., Yang L.F., Ma Y., Ye J.S. (2023). Construction of minitype glutamate sensor for in vivo monitoring of l-glutamate in plant. Microchem. J..

[110] Wang D., Li D., Fu L., Zheng Y., Gu Y., Chen F., Zhao S. (2021). Can Electrochemical Sensors Be Used for Identification and Phylogenetic Studies in Lamiaceae?. Sensors.

[111] Fu L., Zheng Y., Zhang P., Zhang H., Xu Y., Zhou J., Zhang H., Karimi-Maleh H., Lai G., Zhao S. (2020). Development of an electrochemical biosensor for phylogenetic analysis of Amaryllidaceae based on the enhanced electrochemical fingerprint recorded from plant tissue. Biosens. Bioelectron..

[112] Wang Q., Ye W., Li D., Zhu J., Liu C., Lin C., Fu L., Xu Z. (2023). Analysis of Electrochemically Active Substances in Malvaceae Leaves via Electroanalytical Sensing Technology for Species Identification. Micromachines.

[113] Yang R.T., Fan B.Y., Wang S.A., Li L.F., Li Y., Li S.M., Zheng Y.H., Fu L., Lin C.T. (2020). Electrochemical Voltammogram Recording for Identifying Varieties of Ornamental Plants. Micromachines.

[114] Pei C.L., Lu D.Q., Liu D.Y., Pang G.C. (2022). Development of a nanozyme-based electrochemical sensor for detection of stringent response. Anal. Chim. Acta.

[115] Hu J., Shen Y., Zheng Y.H., Zhou W., Karimi-maleh H., Liu Q., Fu L. (2022). Electrochemical fingerprinting sensor for plant phylogenetic investigation: A case of sclerophyllous oak. Front. Plant Sci..

[116] Gandhi M., Amreen K. (2022). Electrochemical Profiling of Plants. Electrochem.

[117] Ciui B., Martin A., Mishra R.K., Nakagawa T., Dawkins T.J., Lyu M., Cristea C., Sandulescu R., Wang J. (2018). Chemical Sensing at the Robot Fingertips: Toward Automated Taste Discrimination in Food Samples. ACS Sens..

[118] Yue X., Luo X., Zhou Z., Bai Y. (2019). Selective electrochemical determination of tertiary butylhydroquinone in edible oils based on an in-situ assembly molecularly imprinted polymer sensor. Food Chem..

[119] Zhao F., He J., Li X., Bai Y., Ying Y., Ping J. (2020). Smart plant-wearable biosensor for in-situ pesticide analysis. Biosens. Bioelectron..

[120] Teixeira S.C., Gomes N.O., Calegaro M.L., Machado S.A.S., de Oliveira T.V., de Fátima Ferreira Soares N., Raymundo-Pereira P.A. (2023). Sustainable plant-wearable sensors for on-site, rapid decentralized detection of pesticides toward precision agriculture and food safety. Biomater. Adv..

[121] Paschoalin R.T., Gomes N.O., Almeida G.F., Bilatto S., Farinas C.S., Machado S.A.S., Mattoso L.H.C., Oliveira O.N., Raymundo-Pereira P.A. (2022). Wearable sensors made with solution-blow spinning poly(lactic acid) for non-enzymatic pesticide detection in agriculture and food safety. Biosens. Bioelectron..

[122] Martins T.S., Machado S.A.S., Oliveira O.N., Bott-Neto J.L. (2023). Optimized paper-based electrochemical sensors treated in acidic media to detect carbendazim on the skin of apple and cabbage. Food Chem..

[123] Zhu X., Lin L., Wu R., Zhu Y., Sheng Y., Nie P., Liu P., Xu L., Wen Y. (2021). Portable wireless intelligent sensing of ultra-trace phytoregulator α-naphthalene acetic acid using self-assembled phosphorene/Ti_3_C_2_-MXene nanohybrid with high ambient stability on laser induced porous graphene as nanozyme flexible electrode. Biosens. Bioelectron..

[124] Perdomo S.A., De la Paz E., Del Caño R., Seker S., Saha T., Wang J., Jaramillo-Botero A. (2023). Non-invasive in-vivo glucose-based stress monitoring in plants. Biosens. Bioelectron..

[125] Perdomo S.A., Valencia D.P., Velez G.E., Jaramillo-Botero A. (2024). Advancing abiotic stress monitoring in plants with a wearable non-destructive real-time salicylic acid laser-induced-graphene sensor. Biosens. Bioelectron..

[126] Seker S., Surucu O., Economou A., Wang J. (2025). “On-plant” wearable electrochemical sensor for atmospheric lead monitoring. Talanta.

[127] Banna G.M.H.U., Siegenthaler J., Benedict A., Allen B., Martinez R.M., Zhang W., Li W. (2024). Heavy metal sensing in plant and soil solutions using carbon fiber electrode. Sens. Actuators A Phys..

[128] Wu B., Xu H., Shi Y., Zhou H., Li Y., Deng H.-d., Ye J., Long Y., Lan Y. (2022). Online Monitoring of Indole-3-acetic Acid in Living Plants Based on Nitrogen-Doped Carbon Nanotubes/Core–shell Au@Cu_2_O Nanoparticles/Carbon Fiber Electrochemical Microsensor. ACS Sustain. Chem. Eng..

[129] Liu D., Li M., Li H., Li C., Wang G., Li P., Yang B. (2021). Core-shell Au@Cu_2_O-graphene-polydopamine interdigitated microelectrode array sensor for in situ determination of salicylic acid in cucumber leaves. Sens. Actuators B Chem..

[130] Ren Q.-Q., Huang X.-R., Liu G.-C., Ou-Yang J., Li M.-T., Chen H., Zhao Y.-D., Chen W. (2015). A field-compatible technique using an electrochemical sensing microbundle for real-time and simultaneous in vivo measurement of hydrogen peroxide, nitric oxide, and pH under drought stress. Sens. Actuators B Chem..

[131] Ren Q.-Q., Yuan X.-J., Huang X.-R., Wen W., Zhao Y.-D., Chen W. (2013). In vivo monitoring of oxidative burst on aloe under salinity stress using hemoglobin and single-walled carbon nanotubes modified carbon fiber ultramicroelectrode. Biosens. Bioelectron..

[132] Sun L., Pan Y., Wu J., Zhao D., Hui M., Zhu S., Zhu X., Li D., Song F., Zhang C. (2020). Paper-Based Analytical Devices for the Rapid and Direct Electrochemical Detection of Hydrogen Peroxide in Tomato Leaves Inoculated with *Botrytis cinerea*. Sensors.

[133] Singh N., Zhang Q., Xu W., Whitham S.A., Dong L. (2025). A Biohydrogel-Enabled Microneedle Sensor for In Situ Monitoring of Reactive Oxygen Species in Plants. ACS Sens..

[134] Diacci C., Abedi T., Lee J.W., Gabrielsson E.O., Berggren M., Simon D.T., Niittylä T., Stavrinidou E. (2021). Diurnal in vivo xylem sap glucose and sucrose monitoring using implantable organic electrochemical transistor sensors. iScience.

[135] Inácio P.M.C., Guerra R., Stallinga P. (2025). A path toward transferable PEDOT:PSS-based capacitive sensors: Electrical modeling and fabrication. Sens. Actuators A Phys..

[136] Sun L.-J., Feng Q.-M., Yan Y.-F., Pan Z.-Q., Li X.-H., Song F.-M., Yang H., Xu J.-J., Bao N., Gu H.-Y. (2014). Paper-based electroanalytical devices for in situ determination of salicylic acid in living tomato leaves. Biosens. Bioelectron..

[137] Bernacka-Wojcik I., Huerta M., Tybrandt K., Karady M., Mulla M.Y., Poxson D.J., Gabrielsson E.O., Ljung K., Simon D.T., Berggren M. (2019). Implantable Organic Electronic Ion Pump Enables ABA Hormone Delivery for Control of Stomata in an Intact Tobacco Plant. Small.

[138] Bukhamsin A., Ait Lahcen A., Filho J.D.O., Shetty S., Blilou I., Kosel J., Salama K.N. (2022). Minimally-invasive, real-time, non-destructive, species-independent phytohormone biosensor for precision farming. Biosens. Bioelectron..

[139] Bukhamsin A.H., Shetty S.S., Fakeih E., Martinez M.S., Lerma C., Mundummal M., Wang J.Y., Kosel J., Al-Babili S., Blilou I. (2025). In vivo dynamics of indole- and phenol-derived plant hormones: Long-term, continuous, and minimally invasive phytohormone sensor. Sci. Adv..

[140] Li H., Wang C., Wang X., Hou P., Luo B., Song P., Pan D., Li A., Chen L. (2019). Disposable stainless steel-based electrochemical microsensor for in vivo determination of indole-3-acetic acid in soybean seedlings. Biosens. Bioelectron..

[141] Khazaee Nejad S., Ma H., Al-Shami A., Soleimani A., Mohamed M.A., Dankwah P., Lee H.J., Mousavi M.P.S. (2024). Sustainable agriculture with LEAFS: A low-cost electrochemical analyzer of foliage stress. Sens. Diagn..

[142] Cancelliere R., Di Tinno A., Cataldo A., Bellucci S., Kumbhat S., Micheli L. (2023). Nafion-based label-free immunosensor as a reliable warning system: The case of AFB1 detection in cattle feed. Microchem. J..

[143] Wang Z., Xue L.F., Li M.J., Li C.P., Li P.H., Li H.J. (2021). Au@SnO_2_-vertical graphene-based microneedle sensor for in-situ determination of abscisic acid in plants. Mater. Sci. Eng. C-Mater. Biol. Appl..

[144] Wang H.R., Bi X.M., Fang Z.J., Yang H.B., Gu H.Y., Sun L.J., Bao N. (2019). Real time sensing of salicylic acid in infected tomato leaves using carbon tape electrodes modified with handed pencil trace. Sens. Actuators B Chem..

[145] Hu Y., Zhao J., Li H., Wang X., Hou P., Wang C., Li A., Chen L. (2018). In vivo detection of salicylic acid in sunflower seedlings under salt stress. RSC Adv..

[146] Vurro F., Janni M., Coppedè N., Gentile F., Manfredi R., Bettelli M., Zappettini A. (2019). Development of an In Vivo Sensor to Monitor the Effects of Vapour Pressure Deficit (VPD) Changes to Improve Water Productivity in Agriculture. Sensors.

[147] Coppedè N., Janni M., Bettelli M., Maida C.L., Gentile F., Villani M., Ruotolo R., Iannotta S., Marmiroli N., Marmiroli M. (2017). An in vivo biosensing, biomimetic electrochemical transistor with applications in plant science and precision farming. Sci. Rep..

[148] Janni M., Coppede N., Bettelli M., Briglia N., Petrozza A., Summerer S., Vurro F., Danzi D., Cellini F., Marmiroli N. (2019). In Vivo Phenotyping for the Early Detection of Drought Stress in Tomato. Plant Phenomics.

[149] Desagani D., Jog A., Teig-Sussholz O., Avni A., Shacham-Diamand Y. (2022). Drought monitoring in tobacco plants by in-vivo electrochemical biosensor. Sens. Actuators B Chem..

[150] Vurro F., Manfredi R., Bettelli M., Bocci G., Cologni A.L., Cornali S., Reggiani R., Marchetti E., Coppedè N., Caselli S. (2023). In vivo sensing to monitor tomato plants in field conditions and optimize crop water management. Precis. Agric..

[151] Ruiz-Gonzalez A., Kempson H., Haseloff J. (2025). Transparent PEDOT:PSS/PDMS Leaf Tattoos for Multiplexed Plant Health Monitoring and Energy Harvesting. Biosensors.

[152] Hossain N.I., Tabassum S. (2023). A hybrid multifunctional physicochemical sensor suite for continuous monitoring of crop health. Sci. Rep..

[153] Ibrahim H., Moru S., Schnable P., Dong L. (2022). Wearable Plant Sensor for In Situ Monitoring of Volatile Organic Compound Emissions from Crops. ACS Sens..

[154] Liu Y., Li M.J., Li H.J., Wang G.L., Long Y.B., Li A.X., Yang B.H. (2019). In Situ Detection of Melatonin and Pyridoxine in Plants Using a CuO-Poly(L-lysine)/Graphene-Based Electrochemical Sensor. ACS Sustain. Chem. Eng..

[155] Camargo J.R., Orzari L.O., Rodrigues J.D., de Lima L.F., Paixao T., Fraceto L.F., Janegitz B.C. (2024). Advancements in disposable electrochemical systems for sustainable agriculture monitoring: Trends, gaps, and applied examples. TrAC Trends Anal. Chem..

[156] Cernat A., Groza A., Tertis M., Feier B., Hosu-Stancioiu O., Cristea C. (2024). Where artificial intelligence stands in the development of electrochemical sensors for healthcare applications—A review. TrAC Trends Anal. Chem..

[157] Matzeu G., Mogas-Soldevila L., Li W., Naidu A., Turner T.H., Gu R., Blumeris P.R., Song P., Pascal D.G., Guidetti G. (2020). Large-Scale Patterning of Reactive Surfaces for Wearable and Environmentally Deployable Sensors. Adv. Mater..

