# Supplementary material for: Electrochemical (Bio)Sensors Based on Nanotechnologies for the Detection of Important Biomolecules in Plants and Plant-Related Samples: The Future of Smart and Precision Agriculture
Source: Biosensors (Basel). 2026 Feb 6;16(2):107. doi: 10.3390/bios16020107 (PMC12938242; doi:10.3390/bios16020107)
Supplement: Supplementary file 1 [file biosensors-16-00107-s001.zip › biosensors-4054858-supplementary.pdf]

Supplementary Materials

Electrochemical (Bio)Sensors Based on Nanotechnologies for the Detection of Important Biomolecules in Plants and Plant-Related Samples: The Future of Smart and Precision Agriculture

Ioana Silvia Hosu [1,*], Radu-Claudiu Fierăscu [1,2,3] and Irina Fierăscu [1,4]

[1] National Institute for Research & Development in Chemistry and Petrochemistry-ICECHIM Bucharest, 202 Spl. Independentei, 6th District, 060021 Bucharest, Romania; fierascu.radu@icechim.ro (R.C.F.); irina.fierascu@icechim.ro (I.F.)
[2] Faculty of Chemical Engineering and Biotechnologies, University "Politehnica" of Bucharest, 313 Splaiul Independentei Str., 060042 Bucharest, Romania
[3] Academy of Romanian Scientists, 3 Ilfov, 050044 Bucharest, Romania
[4] Faculty of Horticulture, University of Agronomic Sciences and Veterinary Medicine of Bucharest, 59 Marasti Blvd., 011464 Bucharest, Romania
* Correspondence: ioana.hosu@icechim.ro or ioana.shosu@yahoo.com

1. Methodology of the review writing

This review is based on extensive research containing literature from the last 14 years, using the following PICO (Problem, Intervention, Comparison, Outcome) strategy.

Table S1. Definition of PICO strategy applied for the present work

P (Problem)	The problem is related to a lack of appropriate chemical (bio)sensors for rapid, accurate real-time quantification of important biomolecules in plants or samples related to plants, *in situ* and *in vivo* (for smart and precision agriculture).
I (Intervention)	The implementation of different nanomaterials/nanotechnologies for electrochemical sensors in plants for real-time monitoring of analytes, using portable devices with smart plant wearable sensors.
C (Comparison)	Gold analytical standards for detection of biomolecules in plants.
O (Outcome)	Improved strategies for *in situ* monitoring plant health and plant treatment screenings, with rapid decision making in farming operations.

The research was conducted based on the PICO question: "What are the nanomaterial-based electrochemical sensors developed and applied for detection of important molecules in plants?" As such, the following inclusion/exclusion criteria were defined:

Inclusion criteria:
1. Research articles published in the time interval 2013–present, full text;
2. Articles published or available in English;
3. Incorporation of nanomaterials (either commercial or obtained in the laboratory);
4. Implementation in plant-based samples (plants/plant extracts/plant tissues);
5. Relevance for the review topic (new information provided);
6. Initial keywords "electrochemical", "sensor", "plant;
7. Within the search, keywords "nano*", "plants", "wearable".

Exclusion criteria:
1. Articles published before 2012;
2. Book chapters or book;

3. Review or systematic review articles;
4. Conference paper, note, letter, short survey, erratum or conference review;
5. Articles published in languages other than English;
6. The keywords "water treatment", "waste water", "water plant" were not included.

The literature search was conducted using the databases SCOPUS and Web Of Science using "electrochemical sensors for plants" as the primary search term. Further selection of the articles was performed automatically, using the inclusion/exclusion criteria defined above, while inclusion in the present review was decided after a full reading of the manuscript.

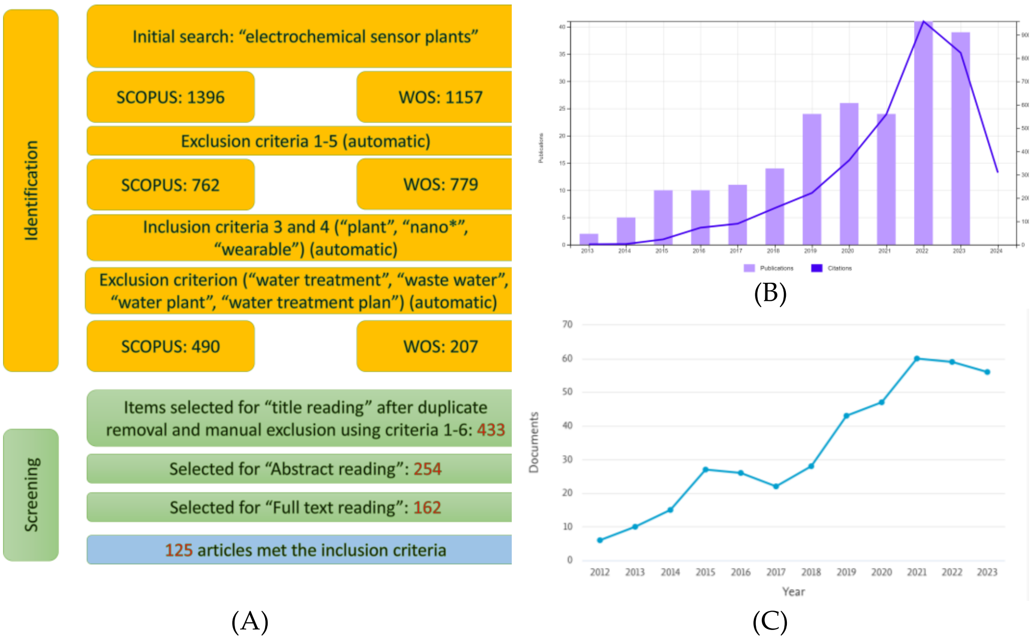

Figure S1. (A) Article selection process flowchart. Publications over time of the selected articles: (B) Web Of Science (WOS) and (C) Scopus.

2. Important molecules in plants and their laboratory-based electrochemical detection

Table S2. Electrochemical detection of different categories of biomolecules detected in plant-related samples, with the corresponding materials and performance electrochemical characteristics of the described methods. An extensive literature study.

Analyte category	Nanomaterials/ Nanotechnology	Analyte/ characteristic	Plant	Method	Analytical parameters (S, LD, LOD)
**Reactive Oxygen Species (ROS)**	Multi-walled carbon nanotube-titanium carbide–palladium, MWCNT-Ti3C2Tx-Pd [1]	H_2O_2	Arabidopsis Thaliana	CA (red. at 0.0 V) M PBS, pH 7.4 DPV (-0.3 V to 0.5 V)	LD= 0.05–18 mM LOD=3.83 μM S = 293.85 μA/ mM^{-1} *cm^{-2}
	AuNPs/MoS2 paper and PtNPs/MoS2 paper [2]	H_2O_2	Aloe	CA (red. at -0.15 V)	LD1= 50-1000 μM LD2= 1000-3000 μM LOD = 10 μM
	Hemoglobin–chitosan/graphene–hexadecyltrimethylammonium bromide [3]	NO	Oilseed rape leaf homogenate samples	CA at -0.7 V	LD= 0.0225 - 2.64 μM LOD= 0.0075 μM

	Double-sided conductive carbon tape modified with multi-walled carbon nanotubes (MWCNTs) [4]	Indole-3-acetic acid (IAA, auxin, wu); salicylic acid (SA)	Pea seedlings	DPV (0.2–1.2 V), step 1 mV, amplitude 5 mV, pulse width 0.02 s, sampling width 0.0067 s, pulse period 0.1 s, quite time 20 s, PBS	NA
	Stainless steel-based PST/Pt-ERGO/Au/a-SS microsensor [5]	Indole-3-acetic acid (IAA)	Soybean seedlings	DPV (0.0 to 1.5 V)	LOD = 43 pg/ml<br>LD = 0.1– 100,000 ng/ml
	TiC@C/Pt-QANFA Microelectrode [6]	Indole-3-acetic acid (IAA)	Single plant protoplast by exocytosis	CA at 0.6 V (vs. Ag/AgCl), PBS	LD = 16 nM - 1.0 μM<br>LOD = 1.0 nM
	Carbon tape electrodes modified with handed pencil trace [7]	Salicylic acid	Infected tomato leaves	SWV (0.2 to 1.3 V) amplitude 25.0 mV, potential step 5.0 mV, frequency 20.0 Hz, quite time 50.0 s.	LD = 1.0 μM – 100 μM<br>LOD = 0.1 μM
**Hormone/ Phyto-hormones**	Core-shell Au@Cu2O-graphene-polydopamine interdigitated microelectrode array sensor [8]	Salicylic acid	Cucumber leaves	DPV	LD = 0.01–100 μM<br>LOD = 1.16 nM
	Copper metal-organic frameworks carboxylated graphene oxide platform (Cu-MOFs–COOH-GO) [9]	Methyl jasmonate	Grape fruit	DPV (-0.8 V to 0.6 V) pulse amplitude 50 mV, pulse period 0.2 s, pulse width of 50 ms, in 0.1 M PBS, pH 7.4	LD = 10 pM to 100 μM<br>LOD = 0.35 μM
	Carboxylated graphene oxide-carboxylated multi-walled carbon nanotubes-Fc and PDANPs-antibody (GO-MWNT-Fc/PDANPs-Ab/SPE) [10]	Gibberellins	Root tips of soybean seedlings	DPV (-0.4 V to 0.6 V, with 0.2 s of pulse period, 50 mV of pulse amplitude, and 50 ms of pulse width) PBS pH 7.4	LD = 100 aM-1 mM<br>LOD = 17.4 aM
	Chemoresistive sensor based on mixture of a copper complex and single-walled carbon nanotubes is placed between two gold electrodes [11]	Ethylene	Ripening of different fruits, before harvest, in a gas chamber	NA	LD = 0.5-50 ppm
**Alkaloids**	Carbon black-modified screen-printed electrode (SPE-CB) [12]	Δ9-tetrahydrocannabinolic acid (Δ9-THCA) and cannabidiolic acid (CBDA)	Cannabis sativa L. plant material (inflorescences)	DPV (0.0 to 1.1 V), 50 mV pulse, 6 mV step, 0.1 s pulse time, 0.4 s interval time (15 mV s−1 scan rate), Britton–Robinson buffer (BRB, pH = 7.0) + EtOH,	Δ9-THCA<br>S_{CB} = (1.22 ± 0.09) × 10^{-1} μA*$μM^{-1}$*cm^{-2}<br>LOD_{CB} = 38.5 μM<br>CBDA<br>S_{CB} = (2.38 ± 0.38) × 10^{-1} μA*$μM^{-1}$*cm^{-2}<br>LOD_{CB} = 29.2 μM<br>RSD_{CB} = 1.6%

	PEDOT-modified screen-printed electrodes (SPE-PEDOT) [12]			0.1 M KCl,	Δ9-THCA LOD_{PEDOT} = 28.2 μM CBDA S_{PEDOT} = (6.3 ± 1.3) × 10^{-2} μA*$μM^{-1}$*cm^{-2} LOD_{PEDOT} = 45.6 μM RSD_{PEDOT} = 4.9%
	Carboxyl-functionalized multi-walled carbon nanotubes (fMWCNTs) + soluble carboxymethylcellulose (CMC), on (GCE) [13]	Theobromine	Green tea, chocolate, and coffee extracts	CV and LSV (0.9 to 1.4 V, 50 mV/s) phosphate buffer solution (pH 7.0)	LD = 0.5 to 80 μM LOD = 0.21 μM
**Proteins/ Amino Acids**	Carbon nanotubes and graphene-modified screen-printed carbon electrodes [14]	Phytochelatins	Hordeum vulgare and Glycine max treated with Hg(II) or Cd(II)	HPLC and CA at 1.0 V vs. Ag/AgCl.	LD = 10^{-6} to 10^{-3} mol L−1 LOD = 0.37-4.38 μmol L^{-1}
	L-glutamate oxidase on m-phenylenediamine + platinum nanoparticles on graphite rod electrode (GluOx/PMPD/Pt/GRE) [15]	Glutamate	Cucumber juice and fruit	CA (+0.4 V vs Ag/AgCl) in 0.1 M PBS pH = 7.4.	LOD = 0.536 μM LD= 2–550 μM RT = 5s
	Polydopamine/graphene/MnO_2 modified glassy carbon electrode PDA/ RGO-MnO_2/GCE [16]	Tryptophan	Tomato fruit and juice	CA at 0.75 V (vs. SCE), 0.1 M PBS, pH 4.0-7.0	LD = 1 - 300 μM S1 = 0.39 μA mM-1 S2= 1.66 μA mM-1, LOD1 = 0.22 μM LOD2 = 0.39 μM
**Other Phyto-chemicals [17]**	Glassy carbon electrode modified via molybdenum disulfide decorated multi-walled carbon nanotubes [18]	Aristolochic acids	Chinese herbs	LSV (-1.0 V to -0.2 V)	LD = 0.2 to 10 μmol/L and 10 to 100 μmol/L, LOD = 0.06 μmol/L S = 3.10 μA/(μmol/L) and -0.91 μA/(μmol/L)
	MoS_2 nanosheets grown on bowl-shaped hollow carbon spheres (MoS_2–BHCs) [19]	Aristolochic acids	*Aristolochia* and *Asarum sieboldii*	SWV (−0.3 to −0.9 V, amplitude 25 mV, frequency 25 Hz) in 0.1 M PBS (pH: 7).	LD_{BHCs} = 0.05–10 μM LOD_{BHCs} = 0.01 μM S_{BHCs} = 1.31 μM RSD = 1.7-3.3%
	MoS_2 on glassy carbon electrode (MoS_2-GCE) [19]	Aristolochic acids	*Aristolochia* and *Asarum sieboldii* sap extracted from cotton leaves	SWV (−0.3 to −0.9 V, amplitude 25 mV, frequency 25 Hz) in 0.1 M PBS (pH: 7).	LD_{GCE} = 10–80 μM LOD_{GCE} = 0.01 μM S_{GCE} = 0.35 μM
**Pathogens**	Target pathogen DNA sequence amplified with RPA/Streptavidin magnetic beads and colloidal gold nanoparticles on SPCE [20]	Pseudomonas syringae	*Arabidopsis T.*	DPV (0.45V to 0.2 V, step 4 mV, amplitude 50 mV, pulse period 0.2 s)	LOD = 214 pM

Gold nanoparticle-functionalized MWCNTs and chitosan-functionalized MWCNT [21]	Ganoderma boninense	Oil palm leaves	LSV 9(-0.6V to +0.6V, potential step of 0.002V, equilibrium time of 10 s, -0.52V potential, 180 s accumulation time, scan rate of 0.06 V/s)	LD = 0.1 to 0.5 mg/L LOD = 0.0414 mg/L
Specific antibodies and AuNPs [22]	Listeria monocytogenes	Wild blueberry samples	CV (4 scans at 100 mV/s) and CA (50 seconds at 300 mV)	LOD = log CFU/mL (or CFU/g)
Pencil graphite electrode (PGE) modified with reduced graphene oxide (rGO) and AuNPs ([23]	*A*grobacterium tumefaciens	Rosa hybrida L.	EIS (impedance, frequency range from 100 kHz to 10 mHz with a potential of 150 mV)	LOD = 0.87×10^{-13} M
Mixed metal oxide-based lateral flow test device [24]	Volatile organic compounds for Botrytis cinerea	Tomato-nose for tomatoes	NA	NA
Non-Faradaic electrochemical impedance spectroscopy [25,26]	Ralstonia solanacearum	Eggplant, potato, tomato, chili, ginger	EIS, sweeping frequency 0.1 Hz - 1 MHz, amplitude of 5 mV and 0 DC bias, 10 mM PBS.	LOD = 0.1 ng/μL
HP2 recognized and nicked by exonuclease and polymerase activity of T4 DNA polymerase and Mg2+-dependent DNAzyme-assisted and hemin/G-quadruplex DNAzyme-assisted cascade amplification strategies, with no disulfide bonds in the glassy electrode (GE) [27]	Watermelon mosaic virus	Seedling of watermelon cultivar Zaochun-hongyu	DPV (-0.65 V to -0.05 V, pulse amplitude 50 mV, pulse width 0.04 s, pulse period of 0.2 s, potential step of 4 mV.	LD = 50 fM to 1 nM LOD = 50 fM
Carbon nanotube (MWCNT)-based copper nanoparticles (CuNPs) on GCE [28]	Agroviruses	Target DNA of agroviruses in sap extracted form cotton leaves	DPV (−0.5 V to 1.2 V, pulse amplitude 50 mV, period 0.2 s, equilibration time 60 s)	LOD = 0.01 ng μL^{-1}
Self-doped polyaniline-DNA hybrid [29]	Gene fragment related to cauliflower mosaic virus 35S gene	Genetically modified plants	CV (+0.8 to -0.6 V, scan rate 100 mV s^{-1}), 0.3M PBS pH 7.0	LD = 1.0 $*10^{-14}$ M - $1.0*10^{-8}$ M LOD = 2.3 -10 $^{-15}$ M
Molecularly imprinted polymer (MIP) in porous polypyrrole [30]	Bean pod mottle virus (BPMV)	Soybean plants	DPV (-0.2 V to 0.5 V)	LD = 0.01−100,000 ng/mL, LOD = 41 pg/mL,

					S= 143 μA ng^{-1} mL cm^{-2}
	Ta_2O_5 sensor chip surfaces coated with a polyelectrolyte interlayer via modularly assembled IgG sensor enzymes [31]	Tobamoviral	N. tabacum L. 'Samsun' nn, N. benthamiana DOMIN	NA	NA
	Screen-printed carbon electrode (SPCE) + electrodeposited gold nanoparticles (AuNPs), immobilizing thiolated ssDNA probes [32]	Citrus tristeza virus	Spikes in leaf extracts from healthy citrus plants	EIS Nyquist plot, PBS pH 7.4	LD= 0.1-10 μM S= 1.4199*ln (μM) LOD=100 nM
**Pesticides/ Herbicide/ Fungicides/ Insecticides/ Pollutants**	Carbon nanotube-modified carbon paste electrode [33]	Diazinon	Food samples related to plants	DPV (-0.3 V to 0.3 V)	LD= 1 x $10^{(-10)}$ to 6 x $10^{(-8)}$ M LOD = 4.5 x $10^{(-10)}$
	PANI/C70/GC [34]	Herbicide triclopyr	Tomatoes plant extracts	SWV (-0.6 to 0.6 V)	LD = 10 $ngmL^{-1}$ - 100 $ngmL^{-1}$ LOD = 1.9 $ngmL^{-1}$
	Nanostructured CuO coatings [35]	Glyphosate	Untreated rye juice	DPV (-1.0 to 1.2 V, 50 mV amplitude, 3 mV step of potential, 50 ms interval time, 25 ms modulation time)	NA
	Chitosan nanoparticles and aluminum silicate-modified carbon paste electrode (CsNP/ALS/MCPE) [36]	Imidacloprid (IDP)	Thyme and guava leaves	Scan rate 100 mV/s (0.0 to 1.3 V), Britton-Robinson buffer, pH 8	LD = 0.02-100 μM LOD = 0.0015 μM
	Bismuth doping on zircon-type gadolinium vanadate [37]	Mesotrione	Extraction from corn	LSV (0 V to - 1.0 V)	LD = 0.1 to 261 μM LOD = 0.1 μM
	Nanostructured multi-walled carbon nanotube paste electrode [38]	Cyprodinil	Apple juice	SWV (0.0 V to 1.0 V, accumulation time 60s, pulse amplitude 40s, frequency 300 s^{-1}, step potential 10 mV) pH 2.0 B-R buffer solution	LD = 0.25–4.0 mg/L LOD = 0.076 mg/L
	Chitosan-stabilized silver nanoparticles electrode (CHI-AgNPs/CPE) [39]	Thiamethoxam	Zea mays and Phaseolus Vulgaris L.	SWV (0.40 V to − 1.40 V, scan rate, pulse amplitude, step, duration: 10 mV s^{-1}, 50 mV, 10 mV and 1 s) in Britton-Robinson buffer (RB) pH 10.4	LD= 0.004 -1.0 mM LOD = 9.32 × 10 -7 M Recoveries 50.4-80.33 %
	Reduced graphene oxide/cyclodextrin-modified glassy carbon electrode [40]	Imidacloprid (IDP)	Extracts of brown rice redissolved in	LSV, -1.0 V to 0.6 V, PBS pH 6.8,	LOD = 0.023 μM LD= 0.5–40 μM S= 1.849 μA/μM/

			0.1 M PBS, pH 6.8		cm^{-2}<br>RSD =92-99%
	A biomimetic device from polydimethylsiloxane (PDMS) [41]	Copper and mechanism of embolism removal in xylem vessels	*Platanus acerifolia*	Differential pulse anodic stripping voltammetry (DPASV) - −0.1 V to 0.4 V, amplitude 25 mV, pulse width 0.02 s,	LD = 10 µg L^{-1} to 100 µg L^{-1}<br>LOD = 0.214 µA µg−1
**Heavy Metals**	Three-dimensional graphene [42]	Cadmium	Rice plants (Klebsiella michiganensis)—bacterial cell and rice tissues	DPV<br>(-1.2 V to 0.4 V)	LD = 0 – 3000 ppm
	Imprinted nanowire-modified PGE [43]	Copper, cadmium, lead	Edible plant products (mushrooms, soybeans, etc.), plant leaves (mango, tomato, etc.), fruits and vegetables	DPSV<br>(+0.4 to -1.2 V, with a scan rate of 10 mV s_1, pulse amplitude of 25 mV, and pulse width of 50 ms)	LD = 4.16 to 205.92 mg L_{-1}<br>LOD = 1.03 mg L_{-1}
**Ions**	Noble metal (palladium, platinum, and gold) ion-chelated DNA/single-walled carbon nanotubes (SWCNTs) [44,45]	H_2, H_2S, NH_3, NO_2 and VOCs	Wild blueberry	Resistance changes before and after exposure to the analyte	Different sensitivities for different analytes
	Carbon-supported PdNi and PdCo bimetallic nanoparticles [46]	Nitrite (NO_2)	Pickled cabbage and bamboo shoot sour juice	Amperometry at 0.9 V, in 0.1 M PBS	LOD = 0.5 µM<br>LD = 10 mM to 1.8 mM<br>S= 5.23 and 5.52 mA mM_1 cm^{-2}
	MWCNTs-COOH Modified CPE [47]	Gallic acid		NA	NA
	MWCNT/graphene/GCE [48]	Gallic acid	Green tea, wine, fruit juice	DPV (-0.3 V to 0.4 V), in PBS pH 6.	LD = 1 - 350<br>LOD = 0.44<br>S = 0.27/0.0704 cm^2
	g-C_3N_4@CNT hetero-junction [49]	Gallic acid	Black tea samples		
	AuNPs on boron nitride nanosheets on GCE (BNNS-AuNPs/GCE) [50]	Luteolin	Peanut hulls and Perilla	SWV (0.1 to 0.3 V) accumulation time 25 min, in PBS pH 6	5-12*10^{-6}<br>0.02^{-10}<br>LOD = 1.7 * 10^{-6}
**Antioxidants**	[51]	Amygdalin	Apple seeds		
	COF-366-Zn/MWCNTs/Nafion/GCE [52]	Tert-butylhydroquinone, butylated hydroxyanisole and phenol	Soybean oil	DPV (-0.4V to 1.0V) in 0.1 M PBS (pH 7.0)	LOD = 0.025 µM, 0.056 µM and 0.051 µM<br>LD = 0.01 to 800 µM for TBHQ, 15–1500

					μM for BHA and 5–500 μM<br>S = 0.5201, 0.1925, 0.213 uM/uA
	Co-doped nitrogen-containing carbon framework/MoS2 [53]	Luteolin	Spiked chrysanthemum, peanut shells and honeysuckle		
	dsDNA/AuNPs/AC@CS/GCE [54]	Antioxidant activity of metabolites	Endophytic fungi *Hypericum perforatum* L.		
	Natural deep eutectic solvents (NADES) composed of glucose, fructose, citric acid and lactic acid [55]	Quercetin	Onion (yellow, red and green) samples	DPV (0.5 to +1.0 V, with 5 mV step potential,<br>25 mV pulse potential, 20 mV s^{-1} scan rate, 0.01 s pulse time, 3 s equilibration time.	LD= 0.026-17<br>LOD = 0.00797
Flavonoids	Pretreated carbon-paste electrode [56]	Quercetin	Extracts of green and black tea	SWV (0.2 V to 0.6 V)	LD = 0.06-2 M<br>LOD = 1.6 x 10($^{-3}$) M<br>S = 19.9 A M^{-1}
	Palladium nanoparticles loaded on carbon sphere @ molybdenum disulfide nanosheet core-shell composites, Cs@MoS2-Pd NPs [57]	Quercetin	Apple juice and green tea	SWV (-0.2 to +0.5 V), PBS pH 6	LD = 0.5 to 12 μM<br>LOD = 0.02 μM<br>S = 0.453 μM/ μA
	Tin disulfide/multi-walled carbon nanotube-modified electrode (SnS_2/CNT/GCE [58]	Rutin	Ethanol extracts of red date, black fruit wolfberry, asparagus, wolfberry, grape peel, tangerine, eggplant peel, buckwheat and apples	DPV (0.3 to 0.7 V, scan rate 100 mV/s) in 0.05 PBS pH 3.0.	LOD = 0.22 nM<br>S = -37.85 μM/μA<br>RSD < 4.18<br>LD = 0.005 - 0.05 μM
	Cyclodextrin-modified MWCNTs (γCD and βCD/MWCNTs) [59]	Rutin	*Arrabidaea brachypoda extract*	SWV (-*0.2 to 1.0 V, 100 mV pulse amplitude, 15 Hz frequency) in PB pH 2.2 at 50 mV/s	LD = 39–975 nM<br>S= 0.469 μA/nM<br>LOD = 7 nM<br>RSD <10%<br>Recovery 96.20%
	TiO_2 or SnO_2 NPs on screen-printed carbon electrodes [60]	*p*-ethylguaiacol	Simulated fruit and leaves volatile system	DPV (-0.1 to 0.7 V, 4 mV step potential, 50 mV amplitude, 0.2 s pulse width, 0.5 s pulse	LD = 0.2–100<br>LOD = 35–62

				period), 0.1 M potassium hydrogen phthalate (KHP) electrolyte	
	Zn-In_2O_3 nanorod-coated glassy carbon microspheres paste electrode [61]	Neuroprotective hibifolin	Flowers of hibiscus vitifolius	SWV	LD = 1.95×10^{-8} to 3.25×10^{-6}<br>LOD = 3.10 × 10^{-10} M
	HKUST-1 loaded on three-dimensional graphene-MWCNT/GCE [62]	Salvianic acid A	Salvia extract or medicinal liquid	DPV (0.0 V to 0.8 V, Increasing Potential: 4 mV; Amplitude: 50 mV; Pulse Width: 50 mV; Pulse Period: 0.5 s) PBS pH 4	LD = 2-4600 μM<br>LOD = 0.081 μM,<br>S = 509.6 μA/mM
	Vinyltrimethoxysilane (VTMS) in multi-walled carbon nanotubes (MWCNTs) and covered by a molecularly imprinted siloxane on GCE (MIS) (MIS/MWCNTs-VTMS/GCE) [63]	Chlorogenic acid	Coffee, tomato,<br><br>and apple samples	DPV (-0.3 V to 0.7V), a pulse amplitude of 50 mV, a scan rate of 20 mV/s), incubation time 15 min.	S = 0.054 μA/ μM<br>LD = 0.08-100<br>LOD = 0.032
	Fe3O4 nanoparticle-modified carbon paste electrode [64]	Sinapic acid, syringic acid, rutin	Wine samples	DPV (0.0 to 1.0 V)	LOD = 2.2 10^{-7} M (sinapic acid)
	TiO2 sensitized with 5-methylphenazinium methosulfate and carboxy-functionalized CdTe quantum dots [65]	Tannic acid	TA in spiked extracts from three medicinal plants	CA at +0.4 V vs Ag/AgCl	LD = 0.2 -200 μmol L^{-1}<br>LOD = 60 nmol L^{-1}
**Markers for Biotic Stress**	Carboxylated graphene–carboxylated multi-walled carbon nanotube–gold nanoparticle–modified electrode [66]	Sugars (glucose, fructose, arabinose, mannose, xylose, and galactose)	Apple juice	CA at 0.3V, in 0.1 M NaOH solution	LD_{GLC} = 5-10 mM<br>LOD_{GLC} = 0.537 μM
	Bi-enzyme electrode: alcohol oxidase and horseradish peroxidase enzymes immobilized onto a multi-walled carbon nanotube (MWCNT), on RDE [67]	Methyl salicylate (VOC)	Wintergreen oil	CA at 0.45 V, with 0.1 s interval	LD = 0-100<br>LOD = 0.98
	Bi-enzyme electrode salicylate hydroxylase and tyrosinase enzymes on a multi-walled carbon nanotube (MWCNTs) matrix through a molecular tethering method, on SPCE [68]	Methyl salicylate (VOC)	Simulated uninfected healthy plant and soybean-aphid-infected plants, in aqueous liquid phase	Constant potential amperometry (CPA) at 0.025 V, with 0.1 s interval, stabilization 2 min	S = 30.67±2.7 μA/(cm^{-2} · μM)<br>LD = 0-27.8<br>LOD = 0.013
**Markers for Abiotic Stress**	Carbon nanotubes (CNTs) reinforced with gold nanoparticles (AuNPs) and chitosan	Quinoline	Infected or healthy leaves extracted	LSV<br>(-0.6 V to 0.6 V)	LD= 0.0004 - 1.0 μM<br>LOD = 3.75 nM

	nanoparticle (CTSNP) multi-walled carbon nanotubes [69]		from oil palms		
DNA/ microRNA	AuNP-coated Bi_2S_3 nanorods on indium tin oxide slides (streptavidin/ microRNA/probe/AuNPs/Bi_2S_3/ITO) [70]	microRNA for the epigenetic regulation of flowering plants.	microRNA-159a in seeds of *Arabidopsis thaliana*	Exposure to 450 nm and measuring current for the 0 V applied voltage, 0.1 M PBS (pH 7.4) containing 0.1M AA	LD = 10 fM to 10 pM<br>LOD = 3.5 fM
	AuNP-coated GCE, with further hybridization of ssDNA with microRNA and immobilization of carboxylic acid graphene–hemin complex (graphene–hemin/bar-code/microRNA/ssDNA/AuNPs/GCE) [71]	microRNA	microRNA-159a in seeds of *Arabidopsis thaliana*	DPV (0.5V to - 0.2 V) step potential 4 mV, amplitude, 50 mV, pulse width 0.05 s, pulse period 0.2 s, quiet time, 2 s.	LD = 0.5 pM - 1.0 nM<br>LOD = 0.17 pM
	Alpha-glucosidase enzyme on amine-functionalized multi-walled carbon nanotubes (MWCNTs-NH_2)/SPCE [72]	Anti-diabetic potential of medicinal plants	Tebengau, Cemumar Kedondong	CV	LD = 0.423-8.29 muA<br>LOD= 0.253 muA<br>S = 0.422 mu A/mg
	Para_nitrophenyl_α_D_glucopyranoside (PNPG)-modified multi-walled carbon nanotube paste electrode [73]	Anti-diabetic potential of medicinal plants	Tebengau Cemumar Kedondong	CV (-0.6 V to 0.6 V)	NA
**Anti-diabetic Potential**	Polydopamine-functionalized graphene sheets [74]	Electrochemical fingerprint-based phylogeny	19 species of Amaryllidaceae	DPV (-0.1 to 1.5 V), positive scans, in ABS, PBS and Tris buffers.	NA
**Anti-cancer Potential**	Unmodified GCE [75]	Identifying Varieties	10 ornamental plants	DPV (-0.1 V to 1.5 V) 0.1 M PBS and 0.1 M ABS	NA
**Anti-HIV Potential**	Unmodified GCE [76]	Identifying species	16 species of Malvaceae group	DPV (-0.1 V to 1.5V) 0.1 M PBS and 0.1 M ABS	NA
	Boron-doped diamond electrode [77]	Wedelolactone (anti-HIV)	Real plant samples	SWV (amplitude: 20 mV, frequency: 25 Hz, step potential: 5 mV, scan rate: 125 mV/s), Britton-Robinson buffer (pH 2.5)	LD = 50-700 ng/mL<br>LOD = 43.87 ng/mL
**Study of Plants**	Unmodified GCE [78]	Phylogenetic investigation	Sclerophyllous oak	DPV (-0.1 V to 1.3 V)	NA
	Nanozyme-based electrochemical [79]	Stringent response	Arabidopsis thaliana/ Escherichia coli	CV (-0.2 to 0.4 V, 50 mV s^{-1} scan rate)	ppGpp ($1.498 * 10^{-12}$ mol/L) NADPH ($7.489 * 10^{-13}$ mol/L)

	extracts				
	Screen-printed carbon-based electrodes on a robotic glove for wearable sensors [80]	Capsaicin for spicy, glucose (GLC) for sweet, ascorbic acid (AA) for sour	Study of taste green pepper, cherry juice, orange juice	SWV, 25 mV amplitude, 15 Hz frequency, −0.2 and +0.8 V (AA), (+0.4 to +1.2 V, caffeine), (−0.2 to +0.8 V, capsaicin). CA at −0.1 V (GLC) 0.1 M PBS (pH 7.4),	S = 4.25 μA/mM (ascorbic acid) S = 4.94 μA/mM (glucose), S = 0.102 μA/ppm (capsaicin)
**Plant Hydration**	Graphite, silver, PEDOT:PSS, gold, and indium tin oxide (ITO)—onto cellulose acetate (CA) [81]	Hydration	No plants, but designed for plant management	Capacitive hydration sensors	Relative

Sensitivity (S, μA/(cm^{-2} · μM), linear domain (LD, μM) and limit of detection (LoD, μM).
GCE= glassy carbon electrode, CA= amperometry, QDs= quantum dots, DPV=differential pulse voltammetry, SWV= square wave voltammetry, RT = response time (s).

3. Future perspectives

In this section we consider describing both technologies that have potential to be applied to plants (but were not) and strategies that were applied to plants, but the technologies and the sensors could benefit from more specificity and tuning for the envisioned target (as most of the sensors presented are not chemically modified for the targeted analyte, but have unique approaches).

3.1. Electrochemical technologies that could be applied to plants or plant related samples

The mycotoxin ochratoxin-A (OTA) was detected using co-immobilized rabbit-immunoglobin antibodies (r-IgGs) and bovine serum albumin (BSA) on nanostructured zinc oxide (Nano-ZnO) film, deposited onto indium–tin–oxide (ITO) glass plate [82]. The BSA/r-IgGs/Nano-ZnO/ITO sensor was calibrated for the mycotoxin using electrochemical impedance spectroscopy (EIS) [82]. ZnO are used for immunosensor applications due to high isoelectric point (IEP~9.5) and biocompatibility, being able to immobilize enzyme with low IEP (r-IgGs; IEP~5.5) through electrostatic interactions. Also, ZnO accelerate electron transfer communication between protein and the electrode, having great potential as a nanomaterial in electrochemistry for plant monitoring or plant related samples analysis.

The epigenetic regulation of flowering plants or the implication plant hormone signaling in the regulation of microRNA gene expression (thus influencing important steps in plant growth), is a future perspective using the streptavidin/microRNA/probe/AuNPs/Bi2S3/ITO method already described in this review [70].

Quercetin was detected in onion samples with the use of Natural Deep Eutectic Solvents (NADES, different combinations of glucose, fructose, citric acid and lactic acid) and screen-printed carbon electrodes (SPCE) [55]. Different percentages of NADES were used to enhance the background electrolyte (phosphate buffer 5 mM, pH 7.6). Different concentrations of quercetin were used to calibrate the method (0.026-17 μM), and the LOD was 7.97 nM, with RSDs of 7.49%. The method was used in onion samples and recoveries differed with <10% when compared with HPLC results, but there were no nanomaterial/nanoparticles involved. Nevertheless, using NADES could be useful for sensors to be used in plants, as interaction of the flavonoid with NADES reduces both charge transfer and reaction resistance of QR. The electronic exchange rate is increased, so the peak potential should be shifted negatively.

Rutin was detected in real samples (rutin tablets) using poly-(3,4-ethylene-dioxythiophene) (PEDOT)- mesoporous carbon (MC) on a GCE electropolymerized in-situ to form AgNPs on the sensor, but the method was not applied to plants [83].

3.1. Unmodified electrochemical sensors

A bio-electrochemical sensor was used to detect GUS enzyme (β-glucuronidase) and gene expression for drought on leaves of *Nicotiana tabacum plants* [84]. Higher quantities of GUS are detected when the plant suffers from drought. The GUS enzyme substrate (4-nitrophenyl β-D-glucuronide, PNPG in PBS) is injected underside of the leaf and it enters the leaf through stomata, where it reacts with GUS enzyme, generating and electroactive products (4-nitrophenol). With the help of an electrochemical chip, the electro-oxidation of 4-nitrophenol appears at 0.4 V, and under drought stress conditions, the current measured at 0.4 V decreases overtime, because of the stomata closing in underwatered plants, even though GUS enzyme concentration increases in stress conditions. The electrochemical chip was fabricated on a silicon wafer (p-Si[100]), followed by a thin 15 n layer of titanium and addition of 200 nm of gold (for both working and auxiliary electrode) and Ag covered with Ag/AgCl for the quasi-reference electrode. The system is validated by measuring the difference of open circuit potential of a fabricated reference electrode and a commercial Ag/AgCl reference electrode [85]. The working electrode was not chemically modified with any nanomaterial, but this kind of modification could be developed in the future.

Reactive oxygen/nitrogen species (ROS/RNS mentioned in the previous section) are formed under drastic environmental conditions (both abiotic or biotic stresses), according to mechanisms involving endogenous signaling pathways. These species are very important for signaling the absence or presence of future or present damage of plants and can be used as an approach for studying and *in situ* monitoring plant health. Needle insertion for creating a cavity in the upper epidermis of *Agave tequilana* leaves, followed by electrode insertion, was used for the detection of H_2O_2 formed under bacterial biotic stress (using *Enterobacter cloacae*) [86]. The sensor used was a dual-function disk platinum microelectrode (50 μm) coated with silver epoxy (so that the coating acts like a pseudo-reference electrode for single miniaturized sensor device). A gold microelectrode (25 μm) modified with iridium oxide was used to assess the pH. Even though physical distress could also enhance the ROS/RNS appearance, the punched hole created in the leave did not give rise to increase in H_2O_2. The increase of H_2O_2 was assessed after only 3 hours after incubation with the stressor. A classical staining fluorescence method with DAB (3,3-diaminobenzidine) was able to detect increased H_2O_2 levels only after 72 hours, the microelectrode being much more rapid. Unstressed plant had pH value of 8.4 and inoculated plants had a pH of 7.4, as bacteria creates acidification of the plant (consistent with salicylic acid, benzoic acid or phenolic acids occurrence).

Volatile cis-3-hexenol and cis-hexenyl acetate that are also secondary metabolites, were detected using simple gold electrodes in [87]. Other molecules have potential to be detected in plants with electrochemical sensors: methyl jasmonate [88], methyl salicylate [89], transgenic plants [90].

Table S3. Future perspectives.

Analyte category	Nanomaterials	Analyte/ characteristic	Plant	Method	Analytical parameters (S, LD, LOD)
Reactive Oxygen Species (ROS)	Platinum disc microelectrode coated with silver epoxy [86]	Hydrogen peroxide (H_2O_2)	Agave tequilana leaves	CV (0.0 V to -1.0 V) in PBS	LD = 100-1000 μM
	Cobalt phthalocyanine screen-printed	Peroxynitrite	-	CA (0.1V)	LOD = 0.4<br>LD = 3–180 μM

	carbon electrode (CoPc/SPCE) [91]				S = 10.843 nA/µM
**Hormones**	FeS2 on cellulose paper [88]	Methyl jasmonate	-		
**Markers for Abiotic Stresses**	Unmodified Au working electrode with Ag/AgCl as quasi-reference electrode [84]	GUS enzyme gene expression for drought	Leaves of Nicotiana tabacum plants	Bio-electrochemical sensing technique	S = NA<br>LD = NA<br>LOD = NA
	AgHg bimetallic particles Nafion-covered Cu electrodes (AgHgNf/Cu and AgBiNf/Cu) [92]	Cysteine and glutathione	Nicotiana tabacum cells exposed to cytotoxic levels of cadmium	DPV (0.5 V as initial potential and −1.2 V as final potential; a pulse height of 2.5 mV, a step height of −5 mV, and a scan rate of 25 mV s−1) Brdička supporting electrolyte solution	Scys = 0.9477 µA/µM<br>S_{GSH} = 0.0485 µA/µM<br>LD_{cys} =2-10µM<br>LD_{GSH}= 20-100 µM<br>LOD_{cys} = 0.088 µM<br>LOD_{GSH} = 0.139 µM
	AgBi bimetallic particles Nafion-covered Cu electrodes (AgHgNf/Cu and AgBiNf/Cu) [92]				S_{cys} = 0.9677 µA/µM<br>S_{GSH} = 0.0226 µA/µM<br>LD_{cys} =2-10 µM<br>LD_{GSH}= 20-100 µM<br>LOD_{cys} = 0.41<br>LOD_{GSH} = 0.244
**Markers for Biotic Stresses**	Bi-enzyme electrode salicylate hydroxylase and tyrosinase enzymes on multi-walled carbon nanotube (MWCNTs) matrix, on SPCE [68]	Methyl salicylate	Simulated uninfected healthy plant and soybean-aphid-infected plants	Constant potential amperometry (CPA) at 0.025 V, with 0.1 s interval, stabilization 2 min	S = 30.67±2.7<br>LD = 0-27.8<br>LOD = 0.013
	Tri-enzyme-based electrochemical sensor [89]	Methyl salicylate			
	Gold electrodes [87]	cis-3-Hexenol, cis-hexenyl acetate, hexyl acetate	Green leaf volatiles (synthetic samples, which imitate both healthy and infected plants)		
**Anti-oxidants**	Poly-(3,4-ethylene-dioxythiophene) (PEDOT)-mesoporous carbon (MC) decorated AgNPs, (GCE/PEDOT-MC/AgNPs) [83]	Rutin		DPV (0.2 to 0.7 V) PBS pH 3, accumulation time 180s	LD= 0.005-100<br>LOD= 0.0035
	Natural deep eutectic solvents (NADES) composed of glucose, fructose, citric acid and lactic acid [55]	Quercetin	Onion (yellow, red and green) samples	DPV (0.5 to +1.0 V, with 5 mV step potential, 25 mV pulse potential, 20 mV s^{-1} scan rate, 0.01 s pulse time, 3 s equilibration time.	0.026-17<br>(0.00797)

	Screen-printed carbon electrode (SPCE)	Quercetin	Onion	DPV (-0.5 to 1.0 V), 5 mV step potential, 25 mV pulse potential, 20 mV/s scan rate, 0.01 s pulse time, 3 s equilibration time)	
	MIP/PdAuNPs/ERGO /GCE [93]	Tertiary butylhydroquinone (TBHQ)	Spiked edible oils (Arowana blend oil)	DPV Amplitude: 0.05V; pulse Width: 0.2 s; Sample Width: 0.0167 s; Pulse Period: 0.5 s Quiet time: 2 s; B-R buffer (pH 2.00)	
**Mycotoxins**	Nano-ZnO rabbit-immunoglobin antibodies (r-IgGs) + bovine serum albumin (BSA)/ indium tin oxide (ITO) glass [82]	Ochratoxin-A (OTA)	-	-	0.006–0.01 nM/dm^3 (0.006 nM/dm^3)
**Transgenic Plants**	Polyaniline-(mesoporous nanozirconia)/ polytyrosine on GCE (PAN-nanoZrO2/PTyr/GCE) [90]				LD = 1.0×10^{-13} mol L^{-1} to 1.0×10^{-6} mol L^{-1} LOD = 2.68×10^{-14} mol L^{-1}
**Pesticides**	A flower-like origami biosensor— 2022 [94]—wearable	Pesticides in aerosol phase (paraoxon, 2,4-D, Glyphosate)	-		$LD_{paraoxon}$ = 2-20 ppb<br>$LD_{2,4\text{-}D}$ = 50-200 ppb<br>$LD_{glyphosate}$ = until 150 ppb<br>$LOD_{paraoxon}$ = 2 ppb<br>$LOD_{2,4\text{-}D}$ = 50 ppb<br>$LOD_{glyphosate}$ = 10 ppb

Sensitivity (S, μA/(cm^{-2} · μM), linear domain (LD, μM) and limit of detection (LoD, μM).

References

1. Zhang, J.; Lu, M.; Zhou, H.; Du, X.; Du, X. Assessment of Salt Stress to Arabidopsis Based on the Detection of Hydrogen Peroxide Released by Leaves Using an Electrochemical Sensor. *International Journal of Molecular Sciences* **2022**, *23*, 12502.
2. Yao, Y.; Liu, X.; Shao, Y.; Ying, Y.; Ping, J. Noble metal alloy nanoparticles coated flexible MoS2 paper for the determination of reactive oxygen species. *Biosensors and Bioelectronics* **2020**, *166*, 112463, doi:https://doi.org/10.1016/j.bios.2020.112463.
3. Wen, W.; Chen, W.; Ren, Q.-Q.; Hu, X.-Y.; Xiong, H.-Y.; Zhang, X.-H.; Wang, S.-F.; Zhao, Y.-D. A highly sensitive nitric oxide biosensor based on hemoglobin–chitosan/graphene–hexadecyltrimethylammonium bromide nanomatrix. *Sensors and Actuators B: Chemical* **2012**, *166-167*, 444-450, doi:https://doi.org/10.1016/j.snb.2012.02.086.
4. Sun, L.-J.; Xie, Y.; Yan, Y.-F.; Yang, H.; Gu, H.-Y.; Bao, N. Paper-based analytical devices for direct electrochemical detection of free IAA and SA in plant samples with the weight of several milligrams. *Sensors and Actuators B: Chemical* **2017**, *247*, 336-342, doi:https://doi.org/10.1016/j.snb.2017.03.025.
5. Li, H.Y.; Wang, C.; Wang, X.D.; Hou, P.C.; Luo, B.; Song, P.; Pan, D.Y.; Li, A.X.; Chen, L.P. Disposable stainless steel-based electrochemical microsensor for in vivo determination of indole-3-acetic acid in soybean seedlings. *Biosensors & Bioelectronics* **2019**, *126*, 193-199, doi:10.1016/j.bios.2018.10.041.
6. Liu, J.T.; Hu, L.S.; Liu, Y.L.; Chen, R.S.; Cheng, Z.; Chen, S.J.; Amatore, C.; Huang, W.H.; Huo, K.F. Real-time monitoring of auxin vesicular exocytotic efflux from single plant protoplasts by amperometry at microelectrodes decorated with nanowires. *Angewandte Chemie - International Edition* **2014**, *53*, 2643-2647, doi:10.1002/anie.201308972.
7. Wang, H.R.; Bi, X.M.; Fang, Z.J.; Yang, H.B.; Gu, H.Y.; Sun, L.J.; Bao, N. Real time sensing of salicylic acid in infected tomato leaves using carbon tape electrodes modified with handed pencil trace. *Sensors and Actuators B-Chemical* **2019**, *286*, 104-110, doi:10.1016/j.snb.2019.01.119.
8. Liu, D.D.; Li, M.J.; Li, H.J.; Li, C.P.; Wang, G.L.; Li, P.H.; Yang, B.H. Core-shell Au@Cu2O-graphene-polydopamine interdigitated microelectrode array sensor for in situ determination of salicylic acid in cucumber leaves. *Sensors and Actuators B-Chemical* **2021**, *341*, doi:10.1016/j.snb.2021.130027.

9. Xing, G.Q.; Wang, C.; Liu, K.; Luo, B.; Hou, P.C.; Wang, X.D.; Dong, H.T.; Wang, J.S.; Li, A.X. A probe-free electrochemical immunosensor for methyl jasmonate based on a Cu-MOF-carboxylated graphene oxide platform. *Rsc Advances* **2022**, *12*, 16688-16695, doi:10.1039/d1ra07683c.
10. You, Y.; Luo, B.; Wang, C.; Dong, H.T.; Wang, X.D.; Hou, P.C.; Sun, L.J.; Li, A.X. An ultrasensitive probe-free electrochemical immunosensor for gibberellins employing polydopamine-antibody nanoparticles modified electrode. *Bioelectrochemistry* **2023**, *150*, doi:10.1016/j.bioelechem.2022.108331.
11. Janssen, S.; Schmitt, K.; Blanke, M.; Bauersfeld, M.L.; Wöllenstein, J.; Lang, W. Ethylene detection in fruit supply chains. *Philosophical Transactions of the Royal Society A: Mathematical, Physical and Engineering Sciences* **2014**, *372*, doi:10.1098/rsta.2013.0311.
12. Monari, A.; Cantalù, S.; Zanfrognini, B.; Brighenti, V.; Verri, P.; Zanardi, C.; Pellati, F.; Pigani, L. An electrochemical approach for the prediction of Δ9-tetrahydrocannabinolic acid and total cannabinoid content in Cannabis sativa L. *Analyst* **2023**, *148*, 4688-4697, doi:10.1039/d3an01090b.
13. Peng, Y.Q.; Zhang, W.J.; Chang, J.; Huang, Y.P.; Chen, L.; Deng, H.; Huang, Z.; Wen, Y.P. A Simple and Sensitive Method for the Voltammetric Analysis of Theobromine in Food Samples Using Nanobiocomposite Sensor. *Food Analytical Methods* **2017**, *10*, 3375-3384, doi:10.1007/s12161-017-0867-5.
14. Dago, A.; Navarro, J.; Arino, C.; Diaz-Cruz, J.M.; Esteban, M. Carbon nanotubes and graphene modified screen-printed carbon electrodes as sensitive sensors for the determination of phytochelatins in plants using liquid chromatography with amperometric detection. *Journal of Chromatography A* **2015**, *1409*, 210-217, doi:10.1016/j.chroma.2015.07.057.
15. Lin, Y.; Yang, L.F.; Ma, Y.; Ye, J.S. Construction of minitype glutamate sensor for in vivo monitoring of l-glutamate in plant. *Microchemical Journal* **2023**, *188*, doi:10.1016/j.microc.2023.108505.
16. Gao, J.; Li, H.; Li, M.; Wang, G.; Long, Y.; Li, P.; Li, C.; Yang, B. Polydopamine/graphene/MnO2 composite-based electrochemical sensor for in situ determination of free tryptophan in plants. *Analytica Chimica Acta* **2021**, *1145*, 103-113, doi:https://doi.org/10.1016/j.aca.2020.11.008.
17. Gandhi, M.; Amreen, K. Electrochemical Profiling of Plants. *Electrochem* **2022**, *3*, 434-450.
18. Wang, Y.; Mamat, X.; Li, Y.T.; Hu, X.; Wang, P.; Dong, Y.M.; Hu, G.Z. Glassy Carbon Electrode Modified via Molybdenum Disulfide Decorated Multiwalled Carbon Nanotubes for Sensitive Voltammetric Detection of Aristolochic Acids. *Electroanalysis* **2019**, *31*, 1390-1400, doi:10.1002/elan.201800893.
19. Zhou, M.; Tang, T.; Deng, X.; Li, Q.; Zuo, Z.; Hu, G. MoS2 nanosheets grown on bowl-shaped hollow carbon spheres as an efficient electrochemical sensor for ultrasensitive determination of nephrotoxic aristolochic acids in Chinese traditional herbs. *Analytical Methods* **2023**, *15*, 3449-3456, doi:10.1039/d3ay00345k.
20. Lau, H.Y.; Wu, H.; Wee, E.J.H.; Trau, M.; Wang, Y.; Botella, J.R. Specific and Sensitive Isothermal Electrochemical Biosensor for Plant Pathogen DNA Detection with Colloidal Gold Nanoparticles as Probes. *Scientific Reports* **2017**, *7*, 38896, doi:10.1038/srep38896.
21. Isha, A.; Akanbi, F.S.; Yusof, N.A.; Osman, R.; Mui-Yun, W.; Abdullah, S.N.A. An NMR Metabolomics Approach and Detection of <i>Ganoderma boninense</i>-Infected Oil Palm Leaves Using MWCNT-Based Electrochemical Sensor. *Journal of Nanomaterials* **2019**, *2019*, 4729706, doi:10.1155/2019/4729706.
22. Davis, D.; Guo, X.; Musavi, L.; Lin, C.-S.; Chen, S.-H.; Wu, V.C.H. Gold Nanoparticle-Modified Carbon Electrode Biosensor for the Detection of Listeria monocytogenes. *Industrial Biotechnology* **2013**, *9*, 31-36, doi:10.1089/ind.2012.0033.
23. Vatankhah, A.; Reezi, S.; Izadi, Z.; Ghasemi-Varnamkhasti, M.; Motamedi, A. Development of an ultrasensitive electrochemical biosensor for detection of Agrobacterium tumefaciens in Rosa hybrida L. *Measurement* **2022**, *187*, 110320, doi:https://doi.org/10.1016/j.measurement.2021.110320.
24. Meléndez, F.; Sánchez, R.; Fernández, J.A.; Belacortu, Y.; Bermúdez, F.; Arroyo, P.; Martín-Vertedor, D.; Lozano, J. Design of a Multisensory Device for Tomato Volatile Compound Detection Based on a Mixed Metal Oxide-Electrochemical Sensor Array and Optical Reader. *Micromachines* **2023**, *14*, doi:10.3390/mi14091761.
25. Patel, R.; Vinchurkar, M.; Shaikh, A.M.; Patkar, R.; Adami, A.; Giacomozzi, F.; Ramesh, R.; Pramanick, B.; Lorenzelli, L.; Baghini, M.S. Part II: Impedance-based DNA biosensor for detection of isolated strains of phytopathogen Ralstonia solanacearum. *Bioelectrochemistry* **2023**, *153*, 108500, doi:https://doi.org/10.1016/j.bioelechem.2023.108500.
26. Patel, R.; Vinchurkar, M.; Mohin Shaikh, A.; Patkar, R.; Adami, A.; Giacomozzi, F.; Ramesh, R.; Pramanick, B.; Lorenzelli, L.; Shojaei Baghini, M. Part I: Non-faradaic electrochemical impedance-based DNA biosensor for detecting phytopathogen - Ralstonia solanacearum. *Bioelectrochemistry* **2023**, *150*, *7*.
27. Wang, Y.; Li, B.; Liu, J.; Zhou, H. T4 DNA polymerase-assisted upgrade of a nicking/polymerization amplification strategy for ultrasensitive electrochemical detection of Watermelon mosaic virus. *Anal Bioanal Chem* **2019**, *411*, 2915-2924.
28. Tahir, M.A.; Bajwa, S.Z.; Mansoor, S.; Briddon, R.W.; Khan, W.S.; Scheffler, B.E.; Amin, I. Evaluation of carbon nanotube based copper nanoparticle composite for the efficient detection of agroviruses. *Journal of Hazardous Materials* **2018**, *346*, 27-35, doi:10.1016/j.jhazmat.2017.12.007.
29. Hu, Y.W.; Yang, T.; Li, Q.H.; Guan, Q.; Jiao, K. Conjugated self-doped polyaniline-DNA hybrid as trigger for highly sensitive reagentless and electrochemical self-signal amplifying DNA hybridization sensing. *Analyst* **2013**, *138*, 1067-1074, doi:10.1039/c2an36620g.

30. Singh, N.; Khan, R.R.; Xu, W.H.; Whitham, S.A.; Dong, L. Plant Virus Sensor for the Rapid Detection of Bean Pod Mottle Virus Using Virus-Specific Nanocavities. *Acs Sensors* **2023**, doi:10.1021/acssensors.3c01478.
31. Wendlandt, T.; Koch, C.; Britz, B.; Liedek, A.; Schmidt, N.; Werner, S.; Gleba, Y.; Vahidpour, F.; Welden, M.; Poghossian, A.; et al. Facile Purification and Use of Tobamoviral Nanocarriers for Antibody-Mediated Display of a Two-Enzyme System. *Viruses-Basel* **2023**, *15*, doi:10.3390/v15091951.
32. Khater, M.; de la Escosura-Muñiz, A.; Quesada-González, D.; Merkoçi, A. Electrochemical detection of plant virus using gold nanoparticle-modified electrodes. *Analytica Chimica Acta* **2019**, *1046*, 123-131, doi:https://doi.org/10.1016/j.aca.2018.09.031.
33. zahirifar, F.; Rahimnejad, M.; Abdulkareem, R.A.; Najafpour, G. Determination of Diazinon in fruit samples using electrochemical sensor based on carbon nanotubes modified carbon paste electrode. *Biocatalysis and Agricultural Biotechnology* **2019**, *20*, 101245, doi:https://doi.org/10.1016/j.bcab.2019.101245.
34. Pandey, A.; Sharma, S.; Jain, R. Voltammetric sensor for the monitoring of hazardous herbicide triclopyr (TCP). *Journal of Hazardous Materials* **2019**, *367*, 246-255, doi:10.1016/j.jhazmat.2018.12.083.
35. Gerbreders, V.; Krasovska, M.; Mihailova, I.; Ogurcovs, A.; Sledevskis, E.; Gerbreders, A.; Tamanis, E.; Kokina, I.; Plaksenkova, I. Nanostructure-based electrochemical sensor: Glyphosate detection and the analysis of genetic changes in rye DNA. *Surfaces and Interfaces* **2021**, *26*, doi:10.1016/j.surfin.2021.101332.
36. Elbaz, G.A.; Zaazaa, H.E.; Monir, H.H.; Abd El Halim, L.M.; Atty, S.A. Nano eco-friendly voltammetric determination of pesticide, imidacloprid and its residues in thyme and guava leaves. *Sustainable Chemistry and Pharmacy* **2022**, *29*, doi:10.1016/j.scp.2022.100799.
37. Karuppaiah, B.; Jeyaraman, A.; Chen, S.-M.; Chavan, P.R.; Karthik, R.; Hasan, M.; Shim, J.-J. Effect of bismuth doping on zircon-type gadolinium vanadate: Effective electrocatalyst for determination of hazardous herbicide mesotrione. *Chemosphere* **2023**, *313*, 137543, doi:https://doi.org/10.1016/j.chemosphere.2022.137543.
38. Ayhan, E.A.; Inam, R. Square wave stripping voltammetric determination of cyprodinil fungicide in food samples by nanostructured multi walled carbon nanotube paste electrode. *Journal of Food Measurement and Characterization* **2020**, *14*, 1333-1343, doi:10.1007/s11694-020-00381-9.
39. Ajermoun, N.; Hrioua, A.; Chhaibi, B.; Laghrib, F.; Farahi, A.; Lahrich, S.; Bakasse, M.; Saqrane, S.; El Mhammedi, M.A. Electrochemical monitoring of thiamethoxam in Zea mays and Phaseolus Vulgaris L. plants using chitosan stabilized silver nanoparticles electrode. *Food Chemistry Advances* **2023**, *3*, 100362, doi:https://doi.org/10.1016/j.focha.2023.100362.
40. Zhao, Y.; Zheng, X.; Wang, Q.; Zhe, T.; Bai, Y.; Bu, T.; Zhang, M.; Wang, L. Electrochemical behavior of reduced graphene oxide/cyclodextrins sensors for ultrasensitive detection of imidacloprid in brown rice. *Food Chemistry* **2020**, *333*, doi:10.1016/j.foodchem.2020.127495.
41. Guo, L.H.; Liu, Y.C.; Liu, L.; Yin, P.H.; Liu, C.; Li, J.M. Study of the mechanism of embolism removal in xylem vessels by using microfluidic devices. *Lab on a Chip* **2023**, *23*, 737-747, doi:10.1039/d2lc00945e.
42. Mitra, S.; Purkait, T.; Pramanik, K.; Maiti, T.K.; Dey, R.S. Three-dimensional graphene for electrochemical detection of Cadmium in Klebsiella michiganensis to study the influence of Cadmium uptake in rice plant. *Materials Science and Engineering C-Materials for Biological Applications* **2019**, *103*, doi:10.1016/j.msec.2019.109802.
43. Roy, E.; Patra, S.; Madhuri, R.; Sharma, P.K. Simultaneous determination of heavy metals in biological samples by a multiple-template imprinting technique: an electrochemical study. *Rsc Advances* **2014**, *4*, 56690-56700, doi:10.1039/c4ra08875a.
44. Sekhon, B.S. Nanotechnology in agri-food production: an overview. *Nanotechnol Sci Appl* **2014**, *7*, 31-53.
45. Su, H.C.; Zhang, M.; Bosze, W.; Lim, J.H.; Myung, N.V. Metal nanoparticles and DNA co-functionalized single-walled carbon nanotube gas sensors. *Nanotechnology* **2013**, *24*, 0957-4484.
46. Xie, Q.J.; He, W.Y.; Yu, S.; Chen, X.Y.; Zhang, X.; Shen, Y.H. Sensitive sensors for amperometric detection of nitrite based on carbon-supported PdNi and PdCo bimetallic nanoparticles. *Analytical Methods* **2014**, *6*, 7716-7721, doi:10.1039/c4ay01461h.
47. Sarafraz, S.; Rafiee-Pour, H.-A.; Khayatkashani, M.; Ebrahimi, A. Electrochemical determination of gallic acid in Camellia sinensis, Viola odorata, Commiphora mukul, and Vitex agnus-castus by MWCNTs-COOH modified CPE. *Journal of Nanostructures* **2019**, *9*, 384-395, doi:10.22052/jns.2019.02.020.
48. Gopal, P.; Reddy, T.M.; Palakollu, V.N. Development, Characterization and Application of a Carbon-Based Nanomaterial Composite as an Electrochemical Sensor for Monitoring Natural Antioxidant (Gallic Acid) in Beverages. *Chemistryselect* **2017**, *2*, 3804-3811, doi:10.1002/slct.201602053.
49. Li, J.D.; Wang, C.X.; Chen, X.L.; Huang, M.H.; Fu, Q.; Li, R.J.; Wang, Y.L.; Li, C.Y.; Zhao, P.C.; Xie, Y.X.; et al. A non-enzymatic photoelectrochemical sensor based on g-C3N4@CNT heterojunction for sensitive detection of antioxidant gallic acid in food. *Food Chemistry* **2022**, *389*, doi:10.1016/j.foodchem.2022.133086.
50. Fu, L.; Liu, Z.; Huang, Y.; Lai, G.; Zhang, H.; Su, W.; Yu, J.; Wang, A.; Lin, C.-T.; Yu, A. Square wave voltammetric quantitative determination of flavonoid luteolin in peanut hulls and Perilla based on Au NPs loaded boron nitride nanosheets. *Journal of Electroanalytical Chemistry* **2018**, *817*, 128-133, doi:https://doi.org/10.1016/j.jelechem.2018.04.009.
51. Chokkareddy, R.; Redhi, G.G. Ionic Liquid and f-MWCNTs Fabricated Glassy Carbon Electrode for Determination of Amygdalin in Apple Seeds. *Electroanalysis* **2020**, *32*, 3045-3053, doi:10.1002/elan.202060401.

52. Jiaojiao, X.; Feng, L.; Lishi, Y.; Hongbo, S.; Jingya, Q.; Bin, Z. Simultaneous determination of tert-butylhydroquinone, butylated hydroxyanisole and phenol in plant oil by metalloporphyrin-based covalent organic framework electrochemical sensor. *Journal of Food Composition and Analysis* **2023**, *122*, doi:10.1016/j.jfca.2023.105486.
53. Wang, Y.L.; Ni, M.J.; Chen, J.; Wang, C.X.; Yang, Y.Q.; Xie, Y.X.; Zhao, P.C.; Fei, J.J. An ultra-sensitive luteolin sensor based on Co-doped nitrogen-containing carbon framework/MoS2-MWCNTs composite for natural sample detection. *Electrochimica Acta* **2023**, *438*, doi:10.1016/j.electacta.2022.141534.
54. Sun, B.L.; Yang, Y.M.; Sun, Y.L.; Wu, D.; Kan, L.; Gao, C.Y.; Shi, H.X.; Sang, C.Y.; Zhao, T.K.; Yang, L.; et al. Evaluating the antioxidant activity of secondary metabolites of endophytic fungi from<i> Hypericum</i><i> perforatum</i> L. by an electrochemical biosensor based on AuNPs/AC@CS composite. *Bioelectrochemistry* **2023**, *151*, doi:10.1016/j.bioelechem.2023.108400.
55. Gomez, F.J.V.; Espino, M.; de los Angeles Fernandez, M.; Raba, J.; Silva, M.F. Enhanced electrochemical detection of quercetin by Natural Deep Eutectic Solvents. *Analytica Chimica Acta* **2016**, *936*, 91-96, doi:https://doi.org/10.1016/j.aca.2016.07.022.
56. Pliuta, K.; Chebotarev, A.; Koicheva, A.; Bevziuk, K.; Snigur, D. Development of a novel voltammetric sensor for the determination of quercetin on an electrochemically pretreated carbon-paste electrode. *Analytical Methods* **2018**, *10*, 1472-1479, doi:10.1039/c7ay02953e.
57. Pei, F.B.; Wu, Y.; Feng, S.S.; Wang, H.L.; He, G.Y.; Hao, Q.L.; Lei, W. Palladium Nanoparticle-Modified Carbon Spheres @ Molybdenum Disulfide Core-Shell Composite for Electrochemically Detecting Quercetin. *Chemosensors* **2022**, *10*, doi:10.3390/chemosensors10020056.
58. Liang, Y.; Zhang, L.Y.; Wang, H.M.; Cai, X.R.; Zhang, L.; Xu, Y.X.; Yao, C.X.; Si, W.S.; Huang, Z.P.; Shi, G.Y. Fabrication of a novel electrochemical sensor based on tin disulfide/multi-walled carbon nanotunbes-modified electrode for rutin determination in natural vegetation. *Arabian Journal of Chemistry* **2023**, *16*, doi:10.1016/j.arabjc.2023.104613.
59. Gomes dos Santos Neto, A.; de Matos Morawski, F.; Caroline Ferreira Santos, A.; Quintino da Rocha, C.; Batista de Lima, R.; Oliveira Fonseca Goulart, M.; Costa dos Santos, C.; Colmati, F.; Euzébio Goulart Santana, A.; Aurélio Suller Garcia, M.; et al. Host-guest Assembly Based on γ-Cyclodextrin-functionalized Multiwalled Carbon Nanotubes for Rutin Electrochemical Sensing. *Electroanalysis* **2023**, *35*, e202200390, doi:https://doi.org/10.1002/elan.202200390.
60. Fang, Y.; Umasankar, Y.; Ramasamy, R.P. Electrochemical detection of p-ethylguaiacol, a fungi infected fruit volatile using metal oxide nanoparticles. *Analyst* **2014**, *139*, 3804-3810, doi:10.1039/c4an00384e.
61. Temerk, Y.; Ibrahim, H. Fabrication of a novel electrochemical sensor based on Zn-In2O3 nanorods coated glassy carbon microspheres paste electrode for square wave voltammetric determination of neuroprotective hibifolin in biological fluids and in the flowers of hibiscus vitifolius. *Journal of Electroanalytical Chemistry* **2016**, *782*, 9-18, doi:10.1016/j.jelechem.2016.09.042.
62. Xing, Y.F.; Zhang, C.; Chen, X.Y.; Zhao, H.M.; Guo, Z.J. Highly sensitive detection of salvianic acid a drug by a novel electrochemical sensor based on HKUST-1 loaded on three-dimensional graphene-MWCNT composite. *Journal of Pharmaceutical and Biomedical Analysis* **2021**, *206*, doi:10.1016/j.jpba.2021.114389.
63. Ribeiro, C.M.; Miguel, E.M.; Silva, J.d.S.; Silva, C.B.d.; Goulart, M.O.F.; Kubota, L.T.; Gonzaga, F.B.; Santos, W.J.R.; Lima, P.R. Application of a nanostructured platform and imprinted sol-gel film for determination of chlorogenic acid in food samples. *Talanta* **2016**, *156-157*, 119-125, doi:https://doi.org/10.1016/j.talanta.2016.05.020.
64. Pwavodi, P.C.; Ozyurt, V.H.; Asir, S.; Ozsoz, M. Electrochemical Sensor for Determination of Various Phenolic Compounds in Wine Samples Using Fe3O4 Nanoparticles Modified Carbon Paste Electrode. *Micromachines* **2021**, *12*, doi:10.3390/mi12030312.
65. Lima, F.M.D.; Freires, A.D.; Pereira, N.D.; Silva, G.G.; da Rocha, C.Q.; Damos, F.S.; Luz, R.D.S. Photoelectrochemical sensing of tannic acid based on the use of TiO2 sensitized with 5-methylphenazinium methosulfate and carboxy-functionalized CdTe quantum dots. *Microchimica Acta* **2018**, *185*, doi:10.1007/s00604-018-3047-4.
66. Liu, K.; Wang, X.D.; Luo, B.; Wang, C.; Hou, P.C.; Dong, H.T.; Li, A.X.; Zhao, C.J. Enzyme-Free Electrochemical Sensors for in situ Quantification of Reducing Sugars Based on Carboxylated Graphene-Carboxylated Multiwalled Carbon Nanotubes-Gold Nanoparticle-Modified Electrode. *Frontiers in Plant Science* **2022**, *13*, doi:10.3389/fpls.2022.872190.
67. Fang, Y.; Umasankar, Y.; Ramasamy, R.P. A novel bi-enzyme electrochemical biosensor for selective and sensitive determination of methyl salicylate. *Biosensors and Bioelectronics* **2016**, *81*, 39-45, doi:https://doi.org/10.1016/j.bios.2016.01.095.
68. Fang, Y.; Bullock, H.; Lee, S.A.; Sekar, N.; Eiteman, M.A.; Whitman, W.B.; Ramasamy, R.P. Detection of methyl salicylate using bi-enzyme electrochemical sensor consisting salicylate hydroxylase and tyrosinase. *Biosensors and Bioelectronics* **2016**, *85*, 603-610, doi:https://doi.org/10.1016/j.bios.2016.05.060.
69. Akanbi, F.S.; Yusof, N.A.; Abdullah, J.; Sulaiman, Y.; Hushiarian, R. Detection of Quinoline in G. boninense-Infected Plants Using Functionalized Multi-Walled Carbon Nanotubes: A Field Study. *Sensors* **2017**, *17*.
70. Wang, M.; Yang, Z.Q.; Guo, Y.L.; Wang, X.X.; Yin, H.S.; Ai, S.Y. Visible-light induced photoelectrochemical biosensor for the detection of microRNA based on Bi2S3 nanorods and streptavidin on an ITO electrode. *Microchimica Acta* **2015**, *182*, 241-248, doi:10.1007/s00604-014-1324-4.

71. Zhou, Y.; Wang, M.; Xu, Z.; Ni, C.; Yin, H.; Ai, S. Investigation of the effect of phytohormone on the expression of microRNA-159a in Arabidopsis thaliana seedlings based on mimic enzyme catalysis systematic electrochemical biosensor. *Biosensors and Bioelectronics* **2014**, *54*, 244-250, doi:https://doi.org/10.1016/j.bios.2013.11.026.
72. Mohiuddin, M.; Arbain, D.; Islam, A.; Ahmad, M.S.; Ahmad, M.N. Alpha-Glucosidase Enzyme Biosensor for the Electrochemical Measurement of Antidiabetic Potential of Medicinal Plants. *Nanoscale Research Letters* **2016**, *11*, doi:10.1186/s11671-016-1292-1.
73. Mohiuddin, M.; Arbain, D.; Shafiqul Islam, A.K.M.; Rahman, M.; Ahmad, M.S.; Ahmad, M.N. Electrochemical measurement of the antidiabetic potential of medicinal plants using multi-walled carbon nanotubes paste electrode. *Russian Journal of Electrochemistry* **2015**, *51*, 368-375, doi:10.1134/s1023193514120027.
74. Fu, L.; Zheng, Y.; Zhang, P.; Zhang, H.; Xu, Y.; Zhou, J.; Zhang, H.; Karimi-Maleh, H.; Lai, G.; Zhao, S.; et al. Development of an electrochemical biosensor for phylogenetic analysis of Amaryllidaceae based on the enhanced electrochemical fingerprint recorded from plant tissue. *Biosensors and Bioelectronics* **2020**, *159*, 112212, doi:https://doi.org/10.1016/j.bios.2020.112212.
75. Yang, R.T.; Fan, B.Y.; Wang, S.A.; Li, L.F.; Li, Y.; Li, S.M.; Zheng, Y.H.; Fu, L.; Lin, C.T. Electrochemical Voltammogram Recording for Identifying Varieties of Ornamental Plants. *Micromachines* **2020**, *11*, doi:10.3390/mi11110967.
76. Wang, Q.; Ye, W.; Li, D.; Zhu, J.; Liu, C.; Lin, C.; Fu, L.; Xu, Z. Analysis of Electrochemically Active Substances in Malvaceae Leaves via Electroanalytical Sensing Technology for Species Identification. *Micromachines* **2023**, *14*.
77. Saxena, S.; Shrivastava, R.; Satsangee, S.P. Voltammetric determination of wedelolactone, an anti-HIV herbal drug, at boron-doped diamond electrode. *Journal of Chemical Sciences* **2015**, *127*, 959-966, doi:10.1007/s12039-015-0853-7.
78. Hu, J.; Shen, Y.; Zheng, Y.H.; Zhou, W.; Karimi-maleh, H.; Liu, Q.; Fu, L. Electrochemical fingerprinting sensor for plant phylogenetic investigation: A case of sclerophyllous oak. *Frontiers in Plant Science* **2022**, *13*, doi:10.3389/fpls.2022.962301.
79. Pei, C.L.; Lu, D.Q.; Liu, D.Y.; Pang, G.C. Development of a nanozyme-based electrochemical sensor for detection of stringent response. *Analytica Chimica Acta* **2022**, *1201*, doi:10.1016/j.aca.2022.339602.
80. Ciui, B.; Martin, A.; Mishra, R.K.; Nakagawa, T.; Dawkins, T.J.; Lyu, M.; Cristea, C.; Sandulescu, R.; Wang, J. Chemical Sensing at the Robot Fingertips: Toward Automated Taste Discrimination in Food Samples. *Acs Sensors* **2018**, *3*, 2375-2384, doi:10.1021/acssensors.8b00778.
81. Inácio, P.M.C.; Guerra, R.; Stallinga, P. A path toward transferable PEDOT:PSS-based capacitive sensors: Electrical modeling and fabrication. *Sensors and Actuators A: Physical* **2025**, *393*, 116779, doi:https://doi.org/10.1016/j.sna.2025.116779.
82. Ansari, A.A.; Kaushik, A.; Solanki, P.R.; Malhotra, B.D. Nanostructured zinc oxide platform for mycotoxin detection. *Bioelectrochemistry* **2010**, *77*, 75-81.
83. Zhang, B.W.; El Jaouhari, A.; Wu, X.R.; Liu, W.; Zhu, J.H.; Liu, X.H. Synthesis and characterization of PEDOT-MC decorated AgNPs for voltammetric detection of rutin in real samples. *Journal of Electroanalytical Chemistry* **2020**, *877*, doi:10.1016/j.jelechem.2020.114632.
84. Desagani, D.; Jog, A.; Teig-Sussholz, O.; Avni, A.; Shacham-Diamand, Y. Drought monitoring in tobacco plants by in-vivo electrochemical biosensor. *Sensors and Actuators B-Chemical* **2022**, *356*, doi:10.1016/j.snb.2021.131357.
85. Li, Z.L.; Zhou, J.P.; Dong, T.; Xu, Y.; Shang, Y.K. Application of electrochemical methods for the detection of abiotic stress biomarkers in plants. *Biosensors & Bioelectronics* **2021**, *182*, doi:10.1016/j.bios.2021.113105.
86. Lima, A.S.; Prieto, K.R.; Santos, C.S.; Paula Valerio, H.; Garcia-Ochoa, E.Y.; Huerta-Robles, A.; Beltran-Garcia, M.J.; Di Mascio, P.; Bertotti, M. In-vivo electrochemical monitoring of H2O2 production induced by root-inoculated endophytic bacteria in Agave tequilana leaves. *Biosensors and Bioelectronics* **2018**, *99*, 108-114, doi:10.1016/j.bios.2017.07.039.
87. Umasankar, Y.; Rains, G.C.; Ramasamy, R.P. Electroanalytical studies on green leaf volatiles for potential sensor development. *Analyst* **2012**, *137*, 3138-3145, doi:10.1039/c2an35350d.
88. Sha, R.; Kadu, A.; Matsumoto, K.; Uno, S.; Badhulika, S. Ultra-low cost, smart sensor based on pyrite FeS2 on cellulose paper for the determination of vital plant hormone methyl jasmonate. *Engineering Research Express* **2020**, *2*, doi:10.1088/2631-8695/ab8bed.
89. Fang, Y.; Zhou, Y.; Ramasamy, R.P. Communication-Direct Detection of Methyl Salicylate Using Tri-Enzyme Based Electrochemical Sensor. *Journal of the Electrochemical Society* **2018**, *165*, B358-B360, doi:10.1149/2.0541809jes.
90. Yang, J.; Wang, X.; Shi, H. An electrochemical DNA biosensor for highly sensitive detection of phosphinothricin acetyltransferase gene sequence based on polyaniline-(mesoporous nanozirconia)/poly-tyrosine film. *Sensors and Actuators B: Chemical* **2012**, *162*, 178-183, doi:https://doi.org/10.1016/j.snb.2011.12.064.
91. Hosu, I.S.; Constantinescu-Aruxandei, D.; Oancea, F.; Doni, M. The Scavenging Effect of Myoglobin from Meat Extracts toward Peroxynitrite Studied with a Flow Injection System Based on Electrochemical Reduction over a Screen-Printed Carbon Electrode Modified with Cobalt Phthalocyanine: Quantification and Kinetics. *Biosensors* **2021**, *11*, 220.
92. Velasco-Medina, C.; Espinoza-Montero, P.J.; Montero-Jimenez, M.; Alvarado, J.; Jadán, M.; Carrera, P.; Fernandez, L. Development and Evaluation of Copper Electrodes, Modified with Bimetallic Nanoparticles, to be Used as Sensors of Cysteine-Rich Peptides Synthesized by Tobacco Cells Exposed to Cytotoxic Levels of Cadmium. *Molecules* **2019**, *24*, 2200.
93. Yue, X.; Luo, X.; Zhou, Z.; Bai, Y. Selective electrochemical determination of tertiary butylhydroquinone in edible oils based on an in-situ assembly molecularly imprinted polymer sensor. *Food Chemistry* **2019**, *289*, 84-94, doi:10.1016/j.foodchem.2019.03.044.

94. Caratelli, V.; Fegatelli, G.; Moscone, D.; Arduini, F. A paper-based electrochemical device for the detection of pesticides in aerosol phase inspired by nature: A flower-like origami biosensor for precision agriculture. *Biosensors and Bioelectronics* **2022**, *205*, 114119, doi:https://doi.org/10.1016/j.bios.2022.114119.
